# Lipidomics-based plasma signature of alcohol-related hepatitis linked to short-term mortality

**DOI:** 10.1016/j.jhepr.2025.101367

**Published:** 2025-03-01

**Authors:** Florent Artru, Stephen Atkinson, Francesca Trovato, Luke D. Tyson, Vishal C. Patel, Nikhil Vergis, Noora Kano, Robert Goldin, Alberto Quaglia, Alexandros Pechlivanis, Phillip Morgan, Salma Mujib, Anna Cavazza, Ellen Jerome, Marc Zentar, Roosey Sheth, Maura Morrison, Evangelos Triantafyllou, Elaine Holmes, María Gómez-Romero, Mark J. McPhail, Mark Thursz

**Affiliations:** 1 Institute of Liver Studies, King’s College Hospital London, London, UK; 2 Institute of Liver Studies, School of Immunology and Microbial Sciences, Faculty of Life Sciences and Medicine, King’s College London, London, UK; 3 Department of Metabolism, Digestion and Reproduction, Division of Digestive Disease, Liver Unit, St Mary’s and Hammersmith Hospitals, Imperial College London, London, UK Imperial College London, London, UK; 4 University of Rennes and Liver Department of Rennes University Hospital, Rennes, France; 5 Roger Williams Institute of Hepatology London, Foundation for Liver Research, London, UK; 6 Section of Bioanalytical Chemistry, Division of Systems Medicine, Department of Metabolism, Digestion and Reproduction, Imperial College London, London, UK

**Keywords:** Alcohol-related hepatitis, Acute-on-chronic liver failure, Cirrhosis, Lipidomics, Glycerophospholipids, Lysophospholipids, Mitochondrial dysfunction, Acylcarnitines

## Abstract

**Background & Aims:**

Severe alcohol-related hepatitis (sAH) is an inflammatory condition with high short-term mortality. Hypothesis-driven approaches have failed to identify effective treatments. Given the role of lipids as inflammatory mediators, this study aimed to identify lipidomic changes and lipid species associated with sAH and mortality risk.

**Methods:**

Untargeted lipidomics was performed on serum samples from two cohorts of patients with sAH and decompensated cirrhosis (DC). Principal component analysis and orthogonal partial least squares discriminant analysis were used to assess lipidome changes. Correlations were made with lipoproteins, lipid mediators, cytokines, cytokeratin fragments, and histological indices.

**Results:**

In the first part, 78 patients with sAH were matched on bilirubin levels with 23 patients with DC. Lipidomics identified a distinct sAH signature involving glycerophospholipids, including PC(34:2) (odds ratio [OR] 2.18, 95% confidence interval [CI] 1.45–7.05, *p* = 0.01), PC(O–38:5) (OR 3.31, 95% CI 2.23–7.14, *p* = 0.002), PI(38:4) (OR 0.71, 95% CI 0.46–0.88, *p* = 0.02), and LPC(18:1) (OR 0.47, 95% CI 0.32–0.82, *p* = 0.01). These lipids demonstrated excellent discriminatory power between sAH and DC with areas under the receiver operating characteristic curve (AUROCs) between 0.87 and 0.88. In the second part, in 159 sAH patients, specific lipids, including carnitines CAR(2:0) (OR 2.51, 95% CI 1.25–4.96, *p* = 0.008) and CAR(16:1) (OR 2.21, 95% CI 1.09–7.48, *p* = 0.009), were linked to 90-day mortality. Acylcarnitines correlated with disease severity parameters such as model for end-stage liver disease, pro-inflammatory cytokines levels, and hepatocyte ballooning on pathology.

**Conclusions:**

Untargeted lipidomics identified a glycerophospholipid and sphingolipid signature distinguishing sAH from DC, implicating lipid species involved in liver regeneration and immune function. Acylcarnitine accumulation in patients with sAH and poor prognosis suggests mitochondrial dysfunction and warrants further investigation into therapeutic potential.

**Impact and implications:**

Lipids can act as mediators at the interface between the immune system and metabolism, potentially contributing to the pathogenesis and outcomes of patients with severe alcohol-related hepatitis, prompting us to investigate lipidomic changes in this population using untargeted approaches, compared with patients with decompensated cirrhosis. This study highlights a distinct lipidomic signature in patients with severe alcohol-related hepatitis compared with decompensated cirrhosis, primarily involving glycerophospholipids and sphingolipids. Specific lipid classes, such as acylcarnitines, suggest significant mitochondrial dysfunction and are associated with disease severity and short-term mortality in patients with severe alcohol-related hepatitis. These findings underscore the importance of targeted investigations into these lipid species, their pathways, and their links to disease severity and outcomes, particularly in this condition that currently lacks specific treatments.

## Highlights

• Untargeted lipidomics revealed a signature distinguishing severe alcohol-related hepatitis from decompensated cirrhosis.
• Mechanisms involving these lipids in the pathogenesis of severe alcohol-related hepatitis must be explored.
• Acylcarnitine accumulation is a key feature distinguishing between survivors and non-survivors in the short term.
• Several short-chain acylcarnitines could serve as potent biomarkers.

## Graphical abstract

**Figure:**
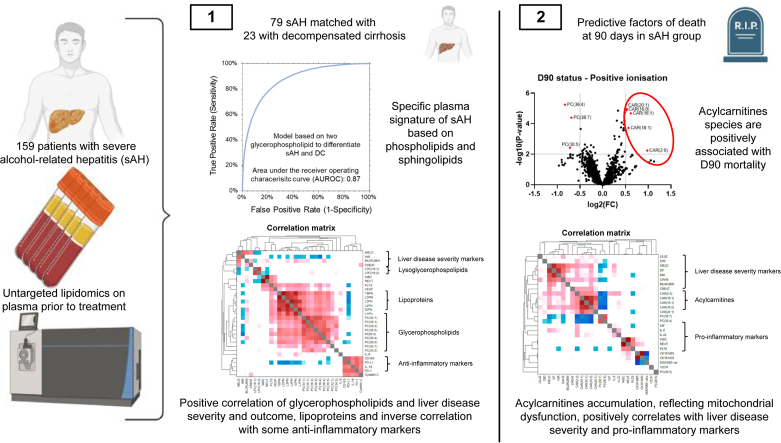

## Introduction

Severe alcohol-associated hepatitis (sAH) affects young patients with a high short-term mortality: 30% of patients will die within the first 90 days.^1^ Liver necroinflammation, defects in hepatic regeneration, gut dysbiosis, systemic inflammation, immune exhaustion, and an increased risk of infection are all contributors to disease pathophysiology and outcome.[2], [3], [4]

These mechanisms have been predominantly investigated using hypothesis-driven approaches, yet research efforts have not resulted in novel therapies that improve outcomes.^2^ Corticosteroids, the only class of drug currently recommended, are associated with a modest reduction in mortality at 28 days but no therapeutic benefit at 3 months or beyond.^5^ To improve therapeutic advances, research in the field of sAH is now applying unbiased approaches.^2^^,^^3^ The use of large multi-omics datasets has allowed researchers to adopt more agnostic approaches and has identified new pivotal mechanisms and new molecular species to target.^3^

Lipids are organic compounds insoluble in water with a variety of metabolic and nonmetabolic functions. They not only represent an efficient energy substrate but can also act as key inflammatory and anti-inflammatory molecules as part of a network of soluble mediators at the interface of metabolism and the immune system.^6^ The role of endogenous bioactive lipid mediators has been demonstrated in several inflammatory diseases (rheumatoid arthritis, inflammatory bowel disease, atherosclerosis, cancer).[6], [7], [8] The liver is unique in providing balanced immunotolerance to the exposure of bacterial components from the gut. Specifically, lipids are involved in the natural course of alcohol-related liver disease. Indeed, alcohol increases the hepatic uptake of fatty acids (FAs), primarily released from adipose tissue, resulting in the intrahepatic accumulation of triglycerides (TGs).[9], [10], [11] Furthermore, alcohol alters FA oxidation and lipid transport, further increasing lipogenesis and steatosis. A recent comprehensive study has revealed that the progression of alcohol-related liver fibrosis is closely linked to significant alterations in the plasma and liver lipidome. These changes predominantly affect sphingolipid (SL) classes, with a marked disruption of their metabolic pathways and a progressive depletion of their components. A depletion in most phosphocholines (PCs) was also observed.^12^ In the setting of decompensated cirrhosis (DC) and acute-on-chronic liver failure (ACLF), untargeted and targeted lipidomics identified a plasma fingerprint of these diseases including eicosanoids, SL, and lysophosphatidylcholines (LPC) that are associated with their dynamic evolution.^13^^,^^14^ In patients with ACLF, the dysregulation of the LPC–autotaxin (ATX)–lysophosphatidic acid (LPA) axis was associated with mortality and inflammation through an LPA-dependent monocyte activation.^15^ Among endogenous bioactive lipids, the role of fatty acyls, including eicosanoids and acylcarnitines, pro-resolving lipid mediators, lysophospholipids, and SL, has been investigated in the setting of sAH. Although recent work has indicated an association between acylcarnitine metabolism and prognosis in sAH, comparisons have not been made with important disease controls such as DC.^16^ Untargeted lipidomics was performed in two large prospectively recruited cohorts of patients with sAH and DC (from the Steroid Or Pentoxifylline for Alcoholic Hepatitis [STOPAH] trial^17^ and the Gut–Liver Axis study) to evaluate whether lipidomics could identify key lipid mediators and their class involved in the pathogenesis of sAH and its complications.

## Materials and methods

### Patients

Patients with sAH were recruited via the STOPAH trial as per the trial protocol.^17^ Briefly, patients were randomised to treatment with prednisolone, pentoxifylline, both, or neither for 28 days using a double-blind, double-dummy, factorial 2 × 2 design. Outcome data were collected for mortality at 28 and 90 days. The Wales Research Ethics Committee (REC 09/MRE09/59) granted ethical approval for this study. To optimise the discovery of metabolites and pathways associated with 90-day mortality and independent of liver disease severity, patients were subselected from the overall cohort based upon (1) the presence of steatohepatitis on biopsy and/or (2) the absence of end-stage liver disease illustrated by model for end-stage liver disease (MELD) score >30. The final cohort included 166 patients with similar liver function assessed using the MELD score. In the sAH cohort, serum samples for all analyses performed in the present study were collected the day of treatment initiation.

Patients with alcohol-related cirrhosis recruited to the prospective longitudinal Compensated Cirrhosis Cohort in Nottingham study (Ref 10/H0403/10; approved by East Midlands Nottingham 1 ethics committee) as well as patients recruited to the Gut–Liver Axis study (London – Westminster Research Ethics Committee No. 12/LO/1417; IRAS No 104301) were used as an additional control group. The study was conducted according to the Declaration of Helsinki (Hong Kong Amendment) and Good Clinical Practice (European guidelines). All participants, or their legally appointed representatives, provided written informed consent. For the purpose of the study, patients with sAH were matched to patients with DC without sAH based on serum bilirubin ±30 μmol/L.

### Lipidomics

Serum lipidomic analyses were performed by ultrahigh performance liquid chromatography coupled to mass spectrometry (UHPLC-MS) after isopropanol protein precipitation (see Supplementary material). MassLynx software 4.1 (Waters, Milford, MA, USA) was used for data acquisition. For the processing of the data, UHPLC-MS raw data files in positive and negative ionisation modes were converted to NetCDF format (using databridge, within MassLynx) and extracted via XCMS (version 1.24.1) package within R (version 2.11; R Foundation for Statistical Computing, Vienna, Austria) software (see Supplementary material). XCMS analysis of these data provided a matrix containing the retention time, *m/z* value, and integrated peak area for aligned features across samples. Every feature of interest identified through supervised multivariable statistical analysis (see below) and volcano plots was subsequently annotated by matching the accurate mass of the molecular ion to reference spectra contained in publicly available databases.

### Lipid mediator-targeted lipidomics

Serum lipid mediators were extracted using solid-phase extraction (SPE) in a mixed-mode plate with a strong anion exchanger (Oasis MAXμElution 96-well plate; Waters). Concentrations were quantified using UHPLC coupled to tandem mass spectrometric detection (MS/MS) using a targeted method^18^ validated to detect up to 48 lipid mediators, of which 26 were quantifiable within this study (see Supplementary material for further details on sample extraction and analysis).

### Liproprotein

Lipoprotein parameters were measured using the B.I. LISA (Bruker Biospin, Billerica, Mass, USA) analysis pipeline for ^1^H-NMR spectroscopy, according to the manufacturer’s instruction.^19^ The spectra generated by standardised sequences can be analysed to quantify a lipoprotein panel of 114 parameters including the main VLDL, intermediate-density lipoprotein (IDL), LDL, and HDL and their subclasses (see Supplementary materials).

### Cytokines, chemokines, and immune and renal markers analyses

Plasma cytokines were measured using Meso Scale Discovery (Meso Scale Diagnostics LLC, Rockville, MD, USA) pro-inflammatory multiplex assay kits, as described in the Supplementary materials. The panel kits included platelet-derived growth factor subunit A (PDGFA); transforming growth factor betas 1, 2, and 3 (TGFβ1, TGFβ2, and TGFβ3); cluster of differentiation 163 (CD163); cystatin C; epidermal growth factor (EGF); hepatocyte growth factor (HGF); interferon (IFN); insulin-like growth factor (IGF); interleukins IL-10, IL-18, IL-1 receptor antagonist (IL-1RA), IL-1α, IL-1β, IL-22, IL-23, IL-6, and IL-8; lipopolysaccharide-binding protein (LBP); neutrophil gelatinase-associated lipocalin (NGAL); programmed death-1 (PD-1); programmed death-ligand 1 (PD-L1); tumour necrosis factor alpha (TNFα); TNF-like weak inducer of apoptosis (TWEAK); and vascular endothelial growth factor (VEGF).

### Serum keratin-18 fragments

The M65 antibody-detected protein reflects total cell death, whereas the M30 antibody-detected fragment is generated when K18 is cleaved during apoptosis. M65/M30 ratio reflects the contribution of apoptosis to cell death pathways.^20^ Quantification of total K18–M65 and caspase-cleaved K18–M30 was performed using ELISA (VLVbio, Stockholm, Sweden).^21^

### Histological analyses

Two experienced histopathologists (RG and AQ), blinded to patient treatment and outcomes, independently assessed the histological features of each biopsy using the alcoholic hepatitis (AH) histological scoring system (AHHSS).^22^ The presence or absence of Mallory–Denk bodies and megamitochondria was also recorded.

### Statistical analyses

Continuous variables were expressed in median and IQR, and categorical variables were expressed in number and percentages. Comparisons between groups were performed using the Mann-Whitney *U* test for quantitative variables or the Chi-square test and Fisher’s exact test for categorical variables, as appropriate. The significance level was set at 0.05 for a two-sided test. Principal components analysis (PCA), orthogonal partial least squares discriminant analysis (OPLS-DA), and the development of scores and volcano plots analyses are detailed in the Supplementary materials. All the analyses were performed through SIMCA version 16.0 (Sartorius Stedim, Aubagne, France), NCSS version 2022 (NCSS, LLC, Kaysville, Utah, USA), and GraphPad Prism version 9.0 (GraphPad Software, San Diego, CA, USA).

## Results

### Exploratory analyses of patients with sAH

Untargeted lipidomics was performed in 166 patients with a clinical diagnosis of sAH according to the STOPAH inclusion criteria.^17^ Among them, 76 were confirmed by liver biopsy, seven patients underwent liver biopsy without any features of the condition after pathology examination, and 83 patients did not undergo liver biopsy. The seven patients with the absence of sAH features on biopsy were excluded from the final analyses. PCA did not demonstrate a visual difference, and OPLS-DA models comparing patients with sAH and a positive biopsy and patients with sAH who were not biopsied (Fig. S1) suggested that from the lipidome side, these patients were not different (comparison of clinical and biological data is provided in Table S1). Consequently, these patients were merged in the same group for final analyses with the final group of sAH (n = 159).

### Patients with sAH have a distinct lipidomic profile compared with patients with DC

In the first part, we aimed to explore the lipidomic signature of patients with sAH compared with matched patients with DC without sAH. To achieve this, we matched up to four patients with sAH to one patient with DC based on serum bilirubin ±30 μmol/L. Hence, 78 of the 159 patients with sAH and 23 of the 74 patients with cirrhosis who underwent untargeted lipidomics were included in these analyses. Comparison between the groups of patients with sAH and cirrhosis after matching on bilirubin level is provided in Table 1. Comparison between the overall sAH cohort (n = 159) and overall patients with cirrhosis (n = 74) is provided in Table S2.

**Table 1:** Characteristics of patients at the time of sampling after matching based on bilirubin level (overall cohort, N = 101; patients with alcohol-related hepatitis, n = 78; patients with cirrhosis, n = 23).

	Population included in the matched analyses (N = 101)	Patients with alcohol-related hepatitis included in the matched analyses (n = 78)	Patients with cirrhosis included in the matched analyses (n = 23)	*p* value
**Characteristics**				
Age (years)	52.0 (45–60.0)	52.0 (44.0–60.0)	52.0 (45.0–61.0)	0.97
Sex (male)	64 (63.4)	46 (61.5)	16 (69.5)	0.48
BMI (kg/m^2^)	24.5 (22.5–29.8)	26.1 (22.5–30.1)	22.9 (22.4–23.8)	0.03
Alcohol-related liver disease	98 (97.0)	78 (100)	20 (86.9)	0.32
Biopsy (yes)	–	38 (48.7)	–	–

**Laboratory**				
Leucocytes (G/L)	8.6 (6.0–11.5)	8.9 (6.2–11.7)	8.0 (5.3–10.9)	0.85
Neutrophils (G/L)	6.0 (3.9–8.9)	6.0 (4.2–8.9)	6.3 (3.6–8.9)	0.42
Haemoglobin (g/L)	104.0 (91.0–116.0)	105.0 (91.0–112.0)	102.0 (89.0–108.0)	0.18
Platelets (G/L)	96.0 (82.0–153.5)	110.0 (75.0–175.0)	66.0 (48.0–116.0)	0.003
INR	1.7 (1.5–2.1)	1.7 (1.5–2.0)	2.0 (1.6–2.8)	0.06
Bilirubin (μmol/L)	253.0 (153.5–359.0)	252.0 (176.0–360.0)	225 (159.0–372.0)	0.29
AST (IU)	120.0 (78.5–155.5)	132.5 (99.5–171.5)	64.0 (49.0–140.0)	0.87
ALP (IU)	170.0 (120.3–254.5)	183.0 (133.0–253.0)	119.0 (82.0–166.0)	0.02
Albumin (g/L)	24.0 (20.5–29.0)	23.0 (20.0–28.0)	26.0 (22.0–33.0)	0.06
Creatinine (μmol/L)	69.0 (55.5–94.0)	64.5 (52.0–80.3)	106 (62.0–167.0)	<0.0001
Urea (mmol/L)	3.4 (2.2–5.7)	3.0 (2.0–4.9)	6.6 (3.3–16.6)	0.0002
Sodium (mmol/L)	134.0 (131.0–137.0)	134.0 (130.0–137.0)	136.0 (133.0–138.0)	0.12

**Scores**				
MELD	22.7 (20.6–26.9)	22.1 (20.5–25.7)	27.6 (22.4–37.6)	0.002
Maddrey’s discriminant function	48.9 (41.1–71.2)	48.9 (41.1–71.2)	–	–
Lille	0.6 (0.3–0.8)	0.6 (0.3–0.8)	–	–

**Outcome**				
Death at 3 months	32 (31.7)	26 (33.3)	6 (26.1)	0.51

Continuous and categorical variables expressed respectively in median (IQR) and n (percentages), respectively. The Mann-Whitney *U* test was used for quantitative variables, and the Chi-square test and Fisher’s exact test were used for categorical variables, as appropriate. ALP, alkaline phosphatase; AST, aspartate aminotransferase; MELD, model for end-stage liver disease.

**Fig. 1:**
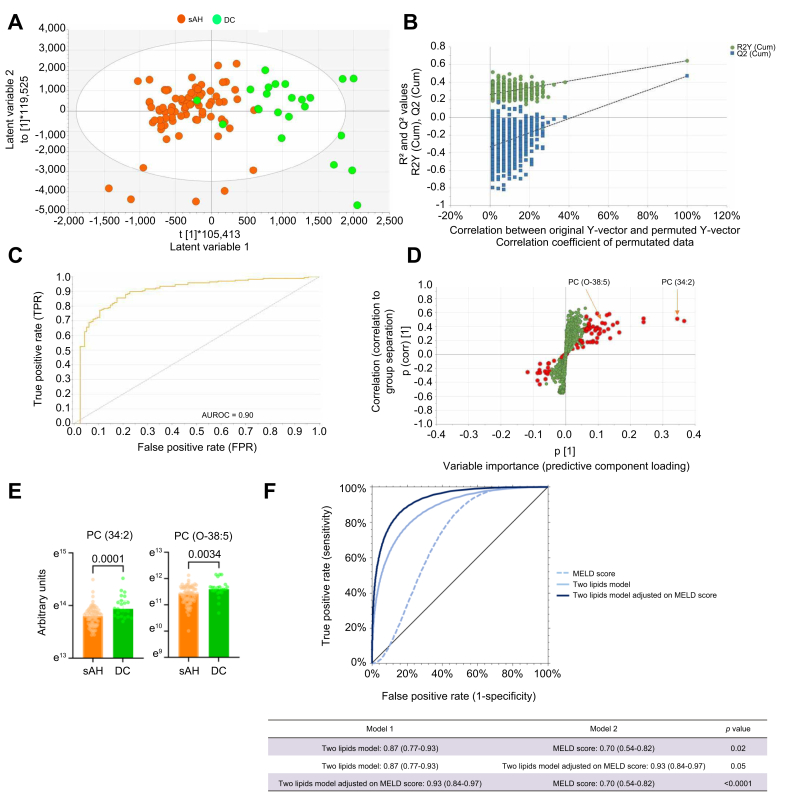
Untargeted lipidomics in positive ionisation mode in patients with sAH (n = 78) matched to patients with DC (n = 23) on bilirubin level. (A) Score plot of the model. Each dot represents the model in one patient: sAH in orange and DC in green. It shows how the samples are grouped based on their lipidome. Two distinct clusters suggest that the two groups are separable from a lipidome perspective. (B) Permutation test demonstrating the validity of the model. A permutation plot tests whether the OPLS-DA model’s ability to separate groups is real or as a result of random chance. A good model shows much higher performance metrics (R^2^ and Q^2^) for the actual data compared with randomised models, with a clear downward trend in performance as the data are permuted. (C) AUROC using the discriminant variables of the OPLS-DA model. (D) S-plot of the model. Each variable is plotted. Variables in red are those with a VIP value ≥2. This plot shows the relationship between the variables’ importance (x-axis) and their correlation with group separation (y-axis). Points far from the centre are the most relevant variables driving the separation. They may correspond to key biomarkers. (E) Univariable analyses of the two lipids identified from multivariable logistic regression as independently differentiating between patients with sAH and DC (PC(34:2), *p* = 0.0001, and PC-O(38:5), *p* = 0.0034, Mann-Whitney *U* test). (F) Performance based on the AUROC of the two-lipid model, two-lipid models adjusted for the MELD score, and the MELD score alone in differentiating patients with sAH from patients with DC. Two-lipid model *vs*. MELD score: *p* = 0.02; two-lipid model *vs*. two-lipid model adjusted for the MELD score: *p* = 0.05; two-lipid model adjusted for the MELD score *vs*. MELD score: *p* <0.0001 (Z test). AUROC, area under the receiver operating characteristic curve; DC, decompensated cirrhosis; MELD, model for end-stage liver disease; OPLS-DA, orthogonal partial least squares discriminant analysis; sAH, severe alcohol-related hepatitis; VIP, variable projection of importance.

In positive ionisation mode, OPLS-DA gave robust differentiation in lipidome between patients with sAH and patients with DC (three-component model 1 + 2 + 0, R^2^ = 0.64, Q^2^ = 0.47, CV-ANOVA *p* <0.0001) (Fig. 1A). A permutations plot confirmed the validity of the model. The area under the receiver operating characteristic curve (AUROC) of the positive ionisation mode model was 0.90 (95% confidence interval [CI] 0.85–0.94, *p* <0.0001) to differentiate between the two conditions (Fig. 1B and C). According to variable projection of importance (VIP) plots, 74 features corresponding to 29 identifiable lipids had a value ≥2 and are illustrated in Fig. 1D and E and listed in Table S3. These lipids mainly belonged to the glycerophospholipid and sphingomyelin subclasses. We inputted these 29 lipids in univariable and multivariable logistic regression analyses. Two lipids, phosphatidylcholine PC(34:2) and PC-O(38:5), independently differentiated between patients with sAH and those with DC even after adjustment for the MELD score (PC(34:2): OR 2.18, 95% CI 1.45–7.05, *p* = 0.01; PC-O(38:5): OR 3.31, 95% CI 2.23–7.4, *p* = 0.002) (Table 2). The model based on these two lipids outperformed the MELD score alone in differentiating sAH from DC with an AUROC of 0.87 (0.77–0.93) *vs*. 0.70 (0.54–0.82) respectively (*p* = 0.02). Adjusting for the MELD score further improved its performance with an AUROC of 0.93 (0.84–0.97, *p* = 0.05) (Fig. 1F).

**Table 2:** Univariable and multivariable logistic regression analyses of lipid species associated with decompensated cirrhosis condition compared with severe alcohol-related hepatitis in positive ionisation mode.

Covariant	Univariable analysis			Multivariable analysis		
	OR	95% CI	*p* value	OR	95% CI	*p* value
PC(34:1)	2.32	1.26–4.28	0.007	1.83	0.50–6.70	0.36
PC(34:2)	2.73	1.54–4.83	0.0006	2.18 2.59∗	1.45–7.05 1.21–7.51∗	0.01 0.02∗
SM(d18:1/16:0)	1.71	1.11–2.65	0.02			
TG(52:2)	1.37	0.88–2.15	0.15			
TG(52:3)	1.45	0.89–2.36	0.13			
PC(36:3)	1.99	1.13–3.50	0.02			
TG(54:3)	0.93	0.58–1.50	0.77			
TG(54:4)	1.43	0.89–2.30	0.14			
TG(54:5)	1.56	0.94–2.56	0.08			
PC(36:1)	0.68	0.43–1.09	0.12			
TG(52:4)	1.27	0.79–2.06	0.32			
PC(36:2)	1.03	0.64–1.05	0.90			
TG(50:2)	0.78	0.92–1.48	0.74			
PC(38:3)	0.57	0.35–0.94	0.03			
TG(50:1)	2.13	1.28–3.56	0.003	1.57	0.64–3.84	0.28
PC(O-38:5)	2.46	1.32–4.57	0.004	3.31 2.82∗	2.23–7.14 1.22–6.54∗	0.002 0.01∗
SM(d18:2/24:0)	0.72	0.47–1.13	0.16			
TG(54:2)	0.68	0.42–1.08	0.11			
TG(50:3)	0.57	0.35–0.91	0.02			
PC(36:5)	1.35	0.82–2.19	0.22			
PC(36:4)	1.54	0.93–2.54	0.09			
TG(48:2)	0.54	0.33–0.87	0.01	0.39	0.15–1.02	0.07
PC(38:4)	0.73	0.45–1.17	0.20			
LPC(16:0)	1.20	0.74–1.97	0.45			
PC(38:5-OH)	2.02	1.14–3.60	0.02			
TG(50:4)	0.53	0.32–0.86	0.01	1.56	0.53–4.57	0.42
TG(56:6)	0.68	0.44–1.06	0.09			
TG(56:8)	0.52	0.32–0.84	0.008	0.67	0.28–1.55	0.45
PC(36:5)	1.70	1.03–2.82	0.04			

Effect size calculated per standard deviation increase. Independent association observed for PC(34:2): *p* = 0.01 and *p* = 0.02 after adjustment for the MELD score; PC(O-38-5): *p* = 0.002 and *p* = 0.01 after adjustment for the MELD score. ∗After adjustment for the MELD score (1.21, 1.08–1.35, *p* = 0.003). CI, confidence interval; MELD, model for end-stage liver disease; OR, odds ratio.

**Fig. 2:**
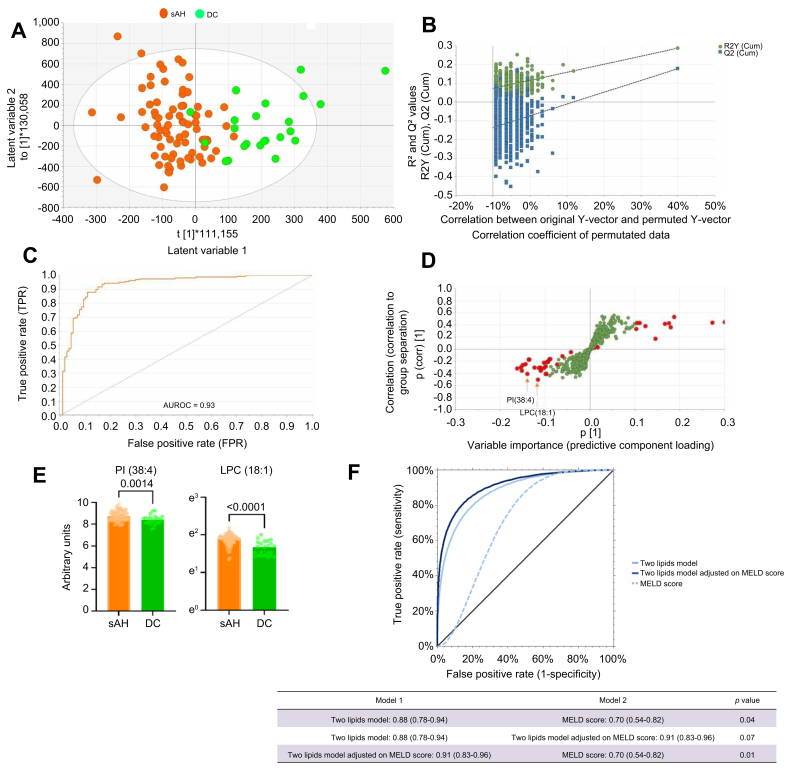
Untargeted lipidomics in negative ionisation mode in patients with sAH (n = 78) matched to patients with DC (n = 23) on bilirubin level. (A) Score plots of the model. Each dot represents the model in one patient: sAH in orange and DC in green. It shows how the samples are grouped based on their lipidome. Two distinct clusters suggest that the two groups are separable from a lipidome perspective. (B) Permutation test demonstrating the validity of the positive ionisation mode. A permutation plot tests whether the OPLS-DA model’s ability to separate groups is real or as a result of random chance. A good model shows much higher performance metrics (R^2^ and Q^2^) for the actual data compared with randomised models, with a clear downward trend in performance as the data are permuted. (C) AUROC using the discriminant variables of the OPLS-DA model. (D) S-plot of the model. Each variable is plotted. Variables in red are those with a VIP value ≥2. This plot shows the relationship between the variables’ importance (x-axis) and their correlation with the group separation (y-axis). Points far from the centre are the most relevant variables driving the separation. They may correspond to key biomarkers. (E) Univariable analyses of the two lipids identified from multivariable logistic regression as independently differentiating between patients with sAH and DC (PI(38:4), *p* = 0.0014, and LPC(18:1), *p* <0.0001, Mann-Whitney *U* test). (F) Performance based on the AUROC of the two-lipid model, two-lipid models adjusted for the MELD score, and MELD score alone in differentiating between patients with sAH and patients with DC. Two-lipid model *vs*. MELD score: *p* = 0.04; two-lipid model *vs*. two-lipid model adjusted for the MELD score: *p* = 0.07; two-lipid model adjusted for the MELD score *vs*. MELD score: *p* = 0.01 (Z test). AUROC, area under the receiver operating characteristic curve; DC, decompensated cirrhosis; MELD, model for end-stage liver disease; OPLS-DA, orthogonal partial least squares discriminant analysis; sAH, severe alcohol-related hepatitis; VIP, variable projection of importance.

In negative ionisation mode, OPLS-DA provided a similarly valid model that was able to differentiate patients with sAH from those with DC (three-component model 1 + 2 + 0, R^2^ = 0.61, Q^2^ = 0.35, CV-ANOVA *p* <0.0001) with an AUROC of 0.93 (95% CI 0.87–0.97, *p* <0.0001) (Fig. 2A–C). Forty features corresponding to 11 identifiable lipids had a value ≥2 in the VIP plot (Fig. 2D and E and Table S4). These were mainly glycerophospholipids and glycophosphoinositols. In univariable and multivariable logistic regression analyses, two lipids were able to independently differentiate between the two conditions even after adjustment for the MELD score: phosphatidylinositol PI(38:4) (OR 0.71, 95%CI 0.46–0.88, *p* = 0.02) and LPC(18:1) (OR 0.47, 95% CI 0.32–0.82, *p* = 0.01) (Fig. 2G and Table 3). The model based on these two lipids outperformed the MELD score alone in differentiating sAH from DC, with an AUROC of 0.88 (0.78–0.94) *vs*. 0.70 (0.54–0.82), respectively (*p* = 0.04). The two-lipid model was not improved after adjustment for the MELD score (0.91, 0.83–0.96, *p* = 0.07) (Figure 2F).

**Table 3:** Univariable and multivariable logistic regression analyses of lipid species associated with decompensated cirrhosis condition compared with severe alcohol-related hepatitis in negative ionisation mode.

Covariant	Univariable analysis			Multivariable analysis		
	OR	95% CI	*p* value	OR	95% CI	*p* value
PC(34:1)	0.53	0.31–0.91	0.02			
PI(38:4)	0.40	0.22–0.71	0.002	0.71 0.75∗	0.46–0.88 0.53–0.98∗	0.02 0.05∗
PC(38:3)	0.53	0.31–0.91	0.02			
PC(38:4)	0.57	0.33–0.98	0.04			
PC(36:1)	0.57	0.32–0.99	0.05			
PC(36:2)	0.80	0.51–1.27	0.35			
PC(36:5)	0.54	0.33–0.91	0.02			
PC(34:2)	1.39	0.86–2.25	0.18			
PC(32:1)	0.72	0.44–1.17	0.18			
LPC(18:1)	0.49	0.32–0.90	0.01	0.47 0.42∗	0.32–0.82 0.27–0.88∗	0.01 0.03∗
LPC(18:2)	0.71	0.46–1.07	0.09			

Effect size calculated per standard deviation increase. Independent association observed for PI(38:4): *p* = 0.02 and *p* = 0.05 after adjustment for the MELD score; LPC(18:1): *p* = 0.01 and *p* = 0.03 after adjustment for the MELD score. ∗After adjustment for the MELD score (1.09, 0.89–1.32, *p* = 0.44). CI, confidence interval; MELD, model for end-stage liver disease; OR, odds ratio.

### Patients with sAH have a distinct lipoprotein and cytokine signature as compared with patients with DC

Lipoproteome of patients with sAH differed from that of patients with DC. Indeed, OPLS-DA of lipoproteomics provided valid models differentiating sAH from DC (Fig. S2A–D). The six lipoprotein parameters with the greatest discriminant abilities were LDL particle number (LDPN), total blood particle number (TBPN), lipoprotein subclass 4 particle number (L4PN), lipoprotein subclass 3 particle number (L3PN), intermediate-density lipoprotein particle number (IDPN), and lipoprotein subclass 1 particle number (L1PN), which were all elevated in sAH compared with DC (Fig. S2G). In addition, among a panel of 27 cytokines, chemokines, and immune and renal markers, eight had different circulating levels between the two conditions: CD163, cystatin C, IL-18, PD-L1, and PD-1 concentrations were decreased in patients with sAH compared with patients with DC; conversely, IL-8, TNFα, and VEGF levels were increased (Fig. S2H). To gain insights into aspects of the clinical phenotype associated with discriminatory lipid species, these lipids were combined with clinical, laboratory, and serum cytokine data, and a bi-clustered cross-correlation matrix was generated and is shown in Fig. S3A (positive ionisation mode lipids) and B (negative ionisation mode lipids). In positive ionisation, we observed co-clustering with positive correlation of the lipoproteins together with several glycerophospholipids and sphingolipids and negative correlation with LPC(16:0) and sphingomyelin SM (d18:1/16:0). LPC(16:0) was strongly negatively correlated with the MELD score (-0.446, *p* <0.0001), bilirubin level (-0.478, *p* <0.0001), IDPN (-0.467, *p* <0.0001), L1PN (-0.347, *p* = 0.002), L3PN (-0.443, *p* <0.0001), L4PN (-0.470, *p* <0.0001), and inflammation markers (white blood cell [WBC] count, neutrophil count, and IL-8) (Fig. S4A). In negative ionisation, we also observed co-clustering of the lipoproteins together with several glycerophospholipids. Among them, PC(36:5) was positively correlated with platelet count (0.42, *p* <0.0001), TBPN (0.502, *p* <0.0001), IDPN (0.401, *p* <0.0001), LDPN (0.508, *p* <0.0001), L3PN (0.422, *p* <0.0001), and L4PN (0.440, *p* <0.0001) and was negatively correlated with CD163 (-0.470, *p* <0.0001), PD-L1 levels (-0.469, *p* <0.0001), and international normalised ratio (INR) (-0.402, *p* <0.0002) (Fig. S4B).

### Exploration of correlation between lipid mediators and key lipids from an untargeted approach

**Fig. 3:**
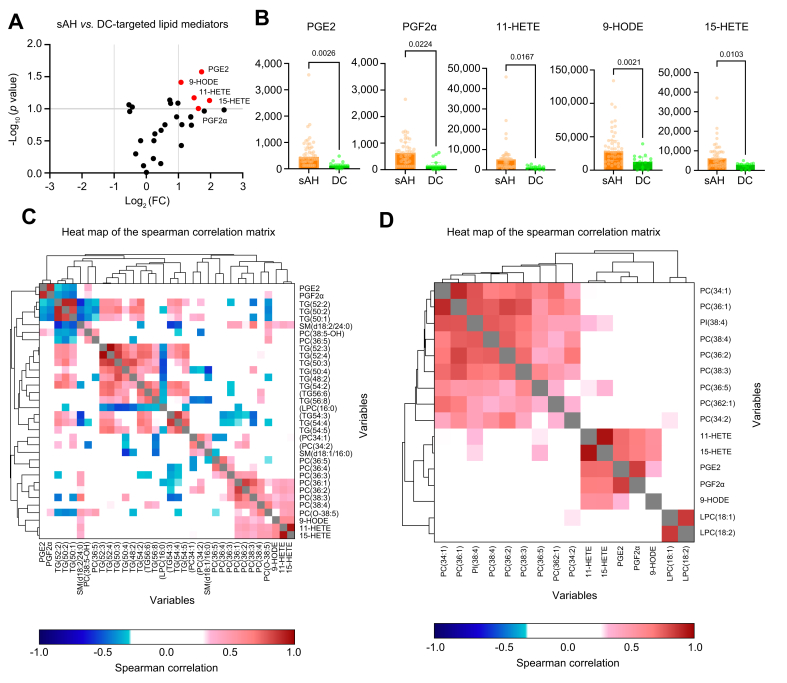
Analyses of lipid mediators in the matched cohort (sAH n = 47; DC n = 15). (A) Volcano plots of each lipid mediators with respect to condition (sAH *vs*. DC). Red plot identifying variables above -1;1 log_2_(FC) threshold and 1 -log_10_(*p* value) threshold. (B) Univariable analysis of the lipid mediators identified in (A) with respect to the condition (sAH *vs*. DC): PGE2, *p* = 0.0026; PG2Fα, *p* = 0.0224; 11-HETE, *p* = 0.0167; 9-HODE, *p* = 0.0021; and 15-HETE, *p* = 0.0103 (Mann-Whitney *U* test). (C) Correlation matrix (Spearman test) including lipid mediators identified in (A) and (B) with lipids with a VIP value ≥2 in positive ionisation mode. (D) Correlation matrix (Spearman test) including lipid mediators identified in (A) and (B) with lipids with a VIP value ≥2 in negative ionisation mode. DC, decompensated cirrhosis; sAH, severe alcohol-related hepatitis; VIP, variable projection of importance.

Forty seven of the 78 patients with sAH (60%) and 15 of the 23 patients with DC (65%) underwent plasma targeted lipid mediators lipidomics. Based on logFCs and *p* values, volcano plots isolated five lipid mediators differentiated between conditions, and all were increased in patients with sAH compared with patients with DC: prostaglandin E2 (PGE2), prostaglandin F2α (PGF2α), 11-hydroxyeicosatetraenoic acid (11-HETE), 9-hydroxyoctadecadienoic acid (9-HODE), and 15-hydroxyeicosatetraenoic acid (15-HETE) (Fig. 3A and B). In positive mode, PC(O-38:5), one of the two lipids included in the positive model, positively correlated with PGF2α (0.403, *p* = 0.0004) 11-HETE (0.405, *p* = 0.0005), and 15-HETE (0.411, *p* = 0.0002) (Fig. 3C). In negative mode, PI(38:4), one of the two lipids included in the negative model, positively correlated with 11-HETE (0.421, *p* = 0.0003) and 15-HETE (0.402, *p* = 0.001) (Fig. 3D).

### Acylcarnitines and phosphatidylcholines are associated with outcome of sAH

**Fig. 4:**
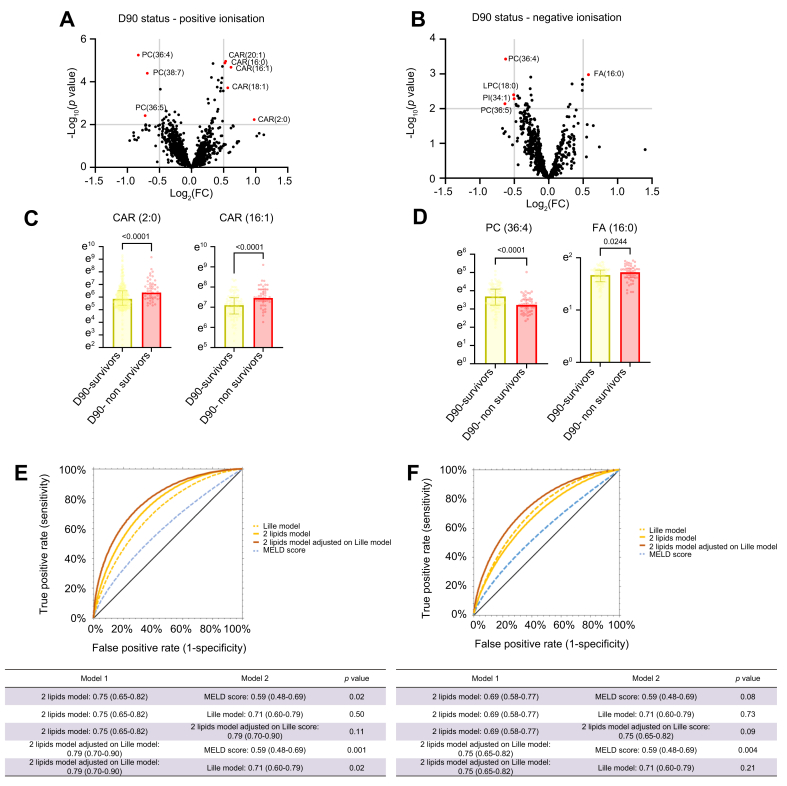
Untargeted lipidomics in restricted to patients with sAH based on status on day 90 (survivors n = 106; non-survivors n = 51). (A) Volcano plots of all features in positive ionisation mode with respect to the D90 status (survivors *vs*. non-survivors). Red plot identifying features above the -0.5;0.5 log_2_(FC) and 2 -log_10_(*p* value according to the Mann-Whitney *U* test) thresholds that were further annotated based on raw chromatograms. (B) Volcano plots of all features of negative ionisation mode with respect to the D90 status (survivors *vs*. non-survivors). Red plot identifying features above the -0.5;0.5 log_2_(FC) and 2 -log_10_(*p* value according to the Mann-Whitney *U* test) thresholds that were further annotated based on raw chromatograms. (C) Univariable analyses of the lipids independently associated with the D90 status in Table 4 in positive ionisation mode: CAR(2:0), *p* <0.0001, and CAR(16:1), *p* <0.0001 (Mann-Whitney *U* test). (D) Univariable analyses of the lipids independently associated with the D90 status in Table S7 in negative ionisation mode: PC(36:4), *p* <0.0001, and FA(16:0), *p* = 0.0244. (E) Performance based on AUROC of the two-lipid model adjusted for the Lille model and MELD score alone, in differentiating D90 survivors from non-survivors in the sAH cohort in positive ionisation mode. Two-lipid model *vs*. MELD score: *p* = 0.02; two-lipid model *vs*. Lille model: *p* = 0.50; two-lipid model *vs*. two-lipid model adjusted for the Lille model: *p* = 0.11; two-lipid model adjusted for the Lille model *vs*. MELD score: *p* = 0.001; two-lipid model adjusted for the Lille model *vs*. Lille model: *p* = 0.02. (F) Performance based on AUROC of the two-lipid model adjusted for the Lille model and MELD score alone in differentiating D90 survivors from non-survivors in the sAH cohort in negative ionisation mode. Two-lipid model *vs*. MELD score: *p* = 0.08; two-lipid model *vs*. Lille model: *p* = 0.73; two-lipid model *vs*. two-lipid model adjusted for the Lille model: *p* = 0.09; two-lipid model adjusted for the Lille model *vs*. MELD score: *p* = 0.004; two-lipid model adjusted for the Lille model *vs*. Lille model: *p* = 0.21 (Z test).

In the cohort of patients with sAH (n = 159), OPLS-DA models in both positive and negative ionisation modes were not robust and/or valid to identify survivors (n = 106) *vs*. non-survivors (n = 51) in the sAH cohort (Fig. S5A–H). Volcano plots isolated eight lipids in positive ionisation mode (Fig. 4A and Table S5) and five lipids in negative ionisation mode (Fig. 4D and Table S6), differentiating survivors from non-survivors at day 90. Univariable and multivariable logistic regression identified two lipids in positive ionisation mode (carnitine CAR(2:0): OR 2.51, 95% CI 1.25–4.96, *p* = 0.008 and CAR(16:1): OR 2.21, 95% CI 1.09–7.48, *p* = 0.009) and two lipids in negative ionisation mode (PC(36:4): OR 0.38, 95%CI 0.15–0.89, *p* = 0.03 and fatty acyl FA(16:0): OR 1.53, 95% CI 1.08–2.19, *p* = 0.02) as independently associated with 90-day mortality status (Fig. 4B and E, Table 4, and Table S7). The resulting model in positive ionisation mode performed better than the Lille model and the MELD score in discriminating between survivors vs. non-survivors at 90 days (Fig. 4C). In negative ionisation mode, the two-lipid model failed to improve the prognostic ability of the Lille model even when combined with it (Fig. 4F). As in the first part, we next explored whether lipoproteome differed between survivors *vs*. non-survivors. The OPLS-DA model was not valid for discriminating between the two conditions (Fig. S6A–D). Volcano plot and univariable analysis identified VLDL subclass 2 cholesterol (V2CH) level as decreased in non-survivors (Fig. S6E and F). Among the 27 cytokines panel, only three differed between survivors and non-survivors and were increased in the latter group: IGF, IL-22, and IL-6 (Fig. S6G). None of the lipid mediators reached the predefined threshold on volcano plots (Fig. S6H). CK-18 M65 and M30 fragments as well as their ratio were evaluated in the cohort and are provided in Fig. S6I.

**Table 4:** Univariable and multivariable logistic regression analyses of lipid species associated with D90 mortality in positive ionisation mode.

Covariant	Univariable analysis			Multivariable analysis		
	OR	95% CI	*p* value	OR	95% CI	*p* value
CAR(2:0)	1.86	1.11–3.16	<0.0001	2.51 3.02∗	1.25–4.96 1.37–6.65∗	0.008 0.005∗
PC(36:5)	0.68	0.44–0.83	0.004	1.51	0.95–2.39	0.09
CAR(18:1)	1.68	1.25–2.14	<0.0001	1.08	0.39–2.98	0.88
PC(38:7)	0.44	0.28–0.73	0.0006	0.30	0.08–1.03	0.19
CAR(16:1)	1.81	1.15–2.89	<0.0001	2.21 1.81∗	1.09–7.48 103–5.12∗	0.009 0.03∗
CAR(16:0)	1.56	1.06–2.12	0.0004	1.71	0.79–4.32	0.41
CAR(20:1)	1.48		0.0001	1.45	0.83–2.54	0.07
PC(36:4)	0.58	0.41–0.72	<0.0001	1.01	0.49–2.09	0.18

Effect size calculated per standard deviation increase. Independent associated observed for CAR(2:0): *p* = 0.008 and *p* = 0.005 after adjustment for the Lille model; CAR(16:1): *p* = 0.009 and *p* = 0.03 after adjustment for the Lille model. ∗After adjustment for the Lille model (13.78, 2.63–72.12, *p* = 0.003). CI, confidence interval; MELD, model for end-stage liver disease; OR, odds ratio.

The correlation matrix of clinical and routine laboratory data, lipids identified in volcano plots, lipoproteins, and cytokines differentiating the D90 status of sAH as well as CK-18 M65, M30 fragments, and their ratio are provided in Fig. S7A (positive ionisation lipids) and B (negative ionisation lipids).

In positive ionisation mode, CAR(2:0), CAR(16:0), and CAR(16:1) negatively co-clustered with phosphatidylcholines PC(38:7) and PC(36:4). Notably, CAR(2:0) was positively correlated with the MELD score (0.363, *p* <0.0001), Glasgow AH score (GAHS) (0.384, *p* <0.0001), creatinine (0.401, *p* <0.0001), IL-6 (0.362, *p* = 0.0006), IGF (0.342, *p* = 0.007), and IL-22 (0.348, *p* = 0.0001) and negatively correlated with PC(36:4) (-0.475, *p* <0.0001) and PC(38:7) (-0.553, *p* <0.0001) (Fig. S8A). In negative ionisation mode, although PC(36:4) and FA(16:0) showed no strong correlation with any other laboratory parameters, scores, cytokines, and lipoproteins included in the correlation matrix, LPC(18:0) showed a strong negative correlation with IL-6 (-0.341, *p* = 0.0001) and biological routine severity data such as the MELD score (-0.461, *p* <0.0001), GAHS score (-0.330, *p* <0.0001), or Lille model (-0.381, *p* = 0.0002) and a positive correlation with PC(36:4) (0.460, *p* <0.0001), which was included in the two-lipid model associated with the D90 status (Fig. S8B).

Association between lipids and baseline infection, response to treatment according to the Lille model, and incident events known to be associated with the prognosis of sAH, such as incident acute kidney injury (AKI) and infection (see definition in Supplementary materials), were further explored. For the analyses of incident AKI, patients with AKI at D0 (n = 82) or those without creatinine level data between D0 and D7 (n = 14) were excluded. Volcano plots identified seven lipids in positive ionisation mode (among which CAR(16:1) and PC(38:7) were already identified as associated with the D90 status) associated with incident AKI status. No lipids in negative ionisation mode were associated with incident AKI status (Fig. S9A and B). Incident infection was defined as infection occurring from the day of treatment initiation to last follow-up.^17^ For these analyses, patients who received antimicrobial treatment within the last 48 h before sampling (n = 38) were excluded. Volcano plots identified 14 lipids in positive ionisation mode (among which PC(36:4) and PC(38:7) were already identified as associated with the D90 status) and four in negative ionisation mode (among which PC(36:4) and PC(37:6) were already associated with the D90 status) associated with incident infection (Fig. S9C and D). Notably, CAR(16:1) and CAR(18:1) were among the 11 lipids identified in the volcano plot as differentiating responders from non-responders according to the Lille model (Fig. S9E–H).

### Pathology

Finally, we aimed to evaluate the relationship between pathology features and key lipids differentiating survivors from non-survivors at D90. We performed a correlation analysis between these lipids, CK-18 M65, M30 fragments, and their ratio and histological features such as AHHSS score, neutrophil infiltration, ballooning, Mallory–Denk bodies, bilirubinostasis, megamitochondria presence, and fibrosis according to Laennec scoring system. Inflammation and steatosis, as defined by AHHSS, were not included as constant in the cohort with positive liver biopsy feature. The correlation matrix with key lipids from positive and negative ionisation modes is provided in Fig. S10A and B, respectively. In positive ionisation mode, ballooning was the only pathology variable associated with lipid signature associated with the D90 status (negatively correlated with PC(36:4) and PC(38:7) and positively with CAR(16:1)). In negative ionisation, the presence of Mallory–Denk bodies was negatively correlated with all lipid signatures except FA(16:0).

## Discussion

In this largest lipidomic analysis of patients with sAH, we identified how lipid biology can distinguish the syndrome from DC and assist in mortality prediction.

A small number of lipid species can be used to differentiate sAH from DC predominantly based on glycerophosphocholines. Concentrations of these lipids correlated with systemic inflammation markers, LDL, and pro-inflammatory lipid mediators. The second part suggests that, in the sAH population, the main lipids associated with clinical events (death, incident AKI, and infection), and some histological features of the disease are acylcarnitines and glycerophosphocholines. Considering the increasing amount of evidence showing that these species can modulate key features of sAH (inflammation, immune dysfunction, and liver regeneration and repair), this study implicates them in the aetiopathogenesis of sAH and provides novel avenues to explore therapeutic targets.

It is important to note that in our study, we selected patients by matching patients with sAH to those with DC based on their serum bilirubin. This was to account for the effect of liver dysfunction and cholestasis on the circulating lipidome. In the second part, the analyses were restricted to patients with sAH from the initial STOPAH cohort to identify metabolites that may predict outcome independently of liver disease severity. As such, the cohort comprised two groups of patients who differed in outcome but who had similar age and MELD scores.

PC/LPC lipid subclasses are key discriminating metabolites between sAH and DC as well as between sAH with favourable and poor outcome (especially regarding the risk of 90-day mortality and incident infection). These lipids regulate liver repair and lipolysis and exhibit immune-modulatory functions (enhancing chemotaxis, stimulating phagocytosis, and upregulating the expression of adhesion molecules) reducing the organ injury and dysfunction in septic shock.[23], [24], [25] LPC injections induced lipoapoptosis of hepatocytes and lobular hepatitis in mice.^26^^,^^27^ In studies evaluating the impact of acute alcohol intoxication in patients with alcohol-related liver disease and non-alcohol-related fatty liver disease, LPC levels were decreased, suggesting an uptake by the liver contributing to caspase activation, endoplasmic reticulum stress, lipoapoptosis, and the development of alcohol-related steatohepatitis in humans.^28^ LPC levels can also decrease through the activation of the LPC–ATX–LPA axis. We recently showed that in both the ACLF and acute liver failure (ALF) settings, this axis was associated with outcomes and that the modulation of LPA, acting through their specific receptor (lysophosphatidic acid receptor [LPAR]), has the potential to reverse the pro-restorative phenotype of circulating monocytes.^15^^,^^29^ In the present lipidomic analysis, LPC(16:0), LPC(18:1), and LPC(18:2) were identified as key lipids differentiating sAH from DC. LPC(16:0) was negatively correlated with pro-inflammatory cytokines, WBC, and neutrophil count, as well as indicators of liver disease severity.

Sphingolipids are represented by ceramides and their precursors sphingomyelins as well as their phosphorylated derivatives sphingosine-1-phosphate (S1P) and ceramide-1-phosphate (C1P). Lysosphingolipids have been implicated in the regulation of a myriad of cell signals, particularly cell survival, adhesion, migration, and barrier integrity, which led to considering sphingolipid metabolism as a true rheostat of the inflammatory processes with pro-inflammatory and anti-inflammatory capacities.^6^^,^[30], [31], [32] Of note, the S1P–S1P receptor (S1PR) pathway is a driver of multiple inflammatory diseases (*e.g*. multiple sclerosis, ulcerative colitis, and rheumatoid arthritis), and there is already a S1PR-targeted drug available.^30^ In liver disease, this axis has been shown to promote liver fibrosis and to regulate liver regeneration, a key altered mechanism in sAH.^30^^,^^33^ Although lower sphingolipid concentrations have been associated with a higher risk of death in sAH, S1P concentration has been shown to be reduced in patients with acute decompensation of cirrhosis and ACLF compared with healthy controls, suggesting an increased activation of the S1P–S1PR pathway.^14^^,^^16^ Here, we observed that several sphingomyelins were part of the signature differentiating sAH from DC and also served as indicators of incident infection and AKI in the sAH population.

Our results suggest that acylcarnitines are enriched in the plasma of patients with sAH with a higher risk of death. Acylcarnitines are long-chain FAs activated in the mitochondria and their accumulation is a marker of mitochondrial dysfunction, which is a key feature observed in patients with sAH.^34^^,^^35^ Indeed, after mitochondrial translocation, acylcarnitines are converted to acyl-CoA, which enters the fatty acid β-oxidation pathway. In the present study, CAR(16:1) correlated with ballooning, a marker of hepatocyte damage, and positively correlated with neutrophils infiltration and Mallory–Denk bodies as well as cell death markers (CK-18 M65 and M30 fragments), which were previously associated with steroid-responsiveness in sAH.^21^ These results are in line with recently published data.^16^ Fatty acid transport and β-oxidation have been shown to be impaired in hepatocytes from patients who underwent liver transplantation in the setting of sAH, suggesting an upstream accumulation of circulating acylcarnitines in patients that are likely to die.^16^ Most acylcarnitines identified in the present work correlated with the MELD score, Glasgow score, and Lille model. Mitochondrial dysfunction has been shown to govern the immunometabolism in circulating leucocytes in ACLF.^36^ Considering the impact of infection incidence in the natural history of patients with sAH and the evidence provided by the present study, therapeutic approaches aiming to resuscitate mitochondrial dysfunction should be further explored. Proof-of-concept studies have already been published in the setting of septic shock as well as in ACLF, where the modulation of anaplerotic reaction aiming to fuel tricarboxylic acid cycle has been demonstrated to improve mitochondrial function.^37^^,^^38^ Such approaches should be investigated in the sAH population. Remarkably, we observed that acetylcarnitine (CAR(2:0)) was one of the two lipids independently associated with the D90 status in positive ionisation mode. CAR(2:0) may be influenced by bacterial translocation, as microbes are a major source of short-chain FAs.^39^ Consequently, CAR(2:0) levels could reflect a combination of the intensity of bacterial translocation and mitochondrial dysfunction, both of which are key features of sAH.

Interestingly, we identified a specific lipoproteomic signature in patients with sAH compared with their matched controls with DC. Although it is known that the overall lipoprotein concentration decreases with the severity of the liver disease,^40^^,^^41^ we reported here an increase in LDL features, commonly reported as exerting pro-inflammatory effects, in patients with sAH compared with those with DC. Such an increase was closely correlated with levels of circulating lysophospholipids and glycerophospholipids. This suggests that in addition to changes in the circulating lipidome, patients with sAH have uniquely impaired metabolic changes that could further participate in promoting systemic inflammation.

This study has several limitations. Firstly, the lipid analysis performed was untargeted and thus lacked the specificity of quantitative techniques targeting one or two lipid classes, providing only relative differences between the samples. An untargeted profiling approach, however, has the potential of discovering novel lipid species not previously described in this kind of disease. In addition, although we have some strong evidence that LPC–ATX–LPA pathway modulation plays an important role in the phenotype of circulating immune cells in ACLF, we have not been able to confirm these findings in the sAH setting in an independent cohort. Further work is needed to investigate the role of sphingolipids and acylcarnitines in sAH. However, in the context of the evidence for the role of sphingolipids and acylcarnitines in other diseases and the robustness of our bioinformatics approach, our study helps identify lipid classes that might play an important role in the key mechanisms that differentiate sAH from DC and lead to complications.

Taken together, the present study underlines the role of several PC/LPC species and sphingolipids in differentiating sAH from DC. These species were also associated with the risk of death, incident infection, and AKI in the sAH population. Considering the growing evidence on the role of these lipids in the regulation of liver regeneration and immune function, they should be promptly explored for therapeutic potential. The acylcarnitine accumulation observed in patients with sAH and poorer prognosis could reflect the degree of mitochondrial dysfunction as well as gut translocation in these patients. Considering the place of mitochondrial dysfunction in the pathogenesis of the disease, prospective exploration of acylcarnitines pathways as a potential target to modulate this dysfunction is also urgently indicated.

## Abbreviations

11/15-HETE, 11/15-hydroxyeicosatetraenoic acid; 9-HODE, 9-hydroxyoctadeca dienoic acid; ACLF, acute-on-chronic liver failure; AHHSS, alcoholic hepatitis histological scoring system; AKI, acute kidney injury; ALF, acute liver failure; ATX, autotaxin; AUROC, area under the receiver operating characteristic curve; C1P, ceramide-1-phosphate; CAR, carnitine; CD163, cluster of differentiation 163; CI, confidence interval; DC, decompensated cirrhosis; EGF, epidermal growth factor; FA, fatty acid; GAHS, Glasgow AH score; HGF, hepatocyte growth factor; IDPN, intermediate-density lipoprotein particle number; IFN, interferon; IGF, insulin-like growth factor; INR, international normalised ratio; L1PN, lipoprotein subclass 1 particle number; L3PN, lipoprotein subclass 3 particle number; L4PN, lipoprotein subclass 4 particle number; LBP, lipopolysaccharide-binding protein; LDPN, LDL particle number; LPA, lysophosphatidic acid; LPAR, lysophosphatidic acid receptor; LPC, lysophosphatidylcholine; MELD, model for end-stage liver disease; NGAL, neutrophil gelatinase-associated lipocalin; OPLS-DA, orthogonal partial least squares discriminant analysis; OR, odds ratio; PC, phosphocholine; PCA, principal components analysis; PD-1, programmed death-1; PD-L1, programmed death-ligand 1; PDGFA, platelet-derived growth factor subunit A; PGE2, prostaglandin E2; PGF2α, prostaglandin F2α; PI, phosphatidylinositol; S1P, sphingosine-1-phosphate; S1PR, S1P–S1P receptor; sAH, severe alcohol-related hepatitis; SL, sphingolipid; SM, sphingomyelin; TBPN, total blood particle number; TG, triglyceride; TGFβ1/2/3, transforming growth factor beta 1/2/3; TNFα, tumour necrosis factor alpha; TWEAK, TNF-like weak inducer of apoptosis; VEGF, vascular endothelial growth factor; VIP, variable projection of importance; WBC, white blood cell.

## Financial support

This study was supported by the National Institute for Health and Care Research (NIHR) Imperial Biomedical Research Centre and the UKRI Medical Research Council (project reference: MR/X009904/1 and MR/R014019/1).

## Authors’ contributions

Design of the study: FA, SA, MM, MT. Acquisition of data: FA, SA, FT, LDT, NV, VCP, MGR, NK, RG, AQ, AP, PM, SM, AC, EJ, MZ, RS, MM, ET, EH, MJM, MT. Statistical analysis: FA, MM. Drafting of the manuscript and critical review: FA, SA, FT, LDT, NV, VCP, MGR, NK, RG, AQ, AP, PM, SM, AC, EJ, MZ, RS, MM, ET, EH, MJM, MT.

## Data availability statement

The data that support the findings of this study are available from the corresponding author upon reasonable request.

## Conflicts of interest

The authors declare no conflicts of interest that pertain to this work.

Please refer to the accompanying ICMJE disclosure forms for further details.
