# Supplementary material for: Lipidomics-based plasma signature of alcohol-related hepatitis linked to short-term mortality
Source: JHEP Rep. 2025 Mar 1;7(6):101367. doi: 10.1016/j.jhepr.2025.101367 (PMC12123345; doi:10.1016/j.jhepr.2025.101367)
Supplement: Multimedia component 1 [file mmc1.pdf]

Lipidomics-based plasma signature of alcohol-related hepatitis linked to short-term mortality

Florent Artru, Stephen Atkinson, Francesca Trovato, Luke D. Tyson, Vishal C. Patel, Nikhil Vergis, Noora Kano, Robert Goldin, Alberto Quaglia, Alexandros Pechlivanis, Phil Morgan, Salma Mujib, Anna Cavazza, Ellen Jerome, Marc Zentar, Roosey Sheth, Maura Morrison, Evangelos Triantafyllou, Elaine Holmes, María Gómez-Romero, Mark J McPhail, Mark Thursz

Table of contents

Supplementary methods 2

Supplementary figures 9

Supplementary tables 29

Supplementary references 36

Supplementary methods

Definition

Incident acute kidney injury (AKI) was defined at D7 by an increase in serum creatinine to ≥1.5 times the patients' assumed baseline creatinine; an increase in creatinine to ≥26.5 μmol/L (0.3 mg/dL) above the baseline creatinine; or the initiation of renal replacement therapy. Patients with an AKI at recruitment (D0) were excluded; as we did not have pre-randomisation or historical values for patients' baseline creatinine available, historical baseline creatinine was assumed to be the lowest creatinine recorded during the STOPAH trial follow-up (up to day 365), discarding results obtained during RRT. Incident infection was defined as those that occurred after the start of treatment.

Untargeted lipidomics

Sample preparation

1. Thawing: Specimen were removed from low temperature storage (-80ºC) and allowed to thaw completely at 4°C.

2. Subsampling: Each thawed specimen was split by first brief vortexing of the original sample immediately followed by removal of the required volume of sample to a 2 mL eppendorf tube using an appropriate pipette and individual single-use tips to prevent sample cross contamination.

3. Randomisation: Specimen were randomised and given a new sequential numeric processing code prior to preparation. This is done to avoid batching in subsequent steps.

4. Protein precipitation: Serum samples were prepared for UHPLC-MS lipid analysis by isopropanol (IPA) protein precipitation (1). IPA was placed in the fridge a day prior to precipitation use. Cold IPA (1:3 (v/v) sample:solvent) was added to serum, vortexed and incubated at -20˚C for 2 hours. b) Samples were centrifuged at 4°C for 10 minutes at 130 000 rpm. c) The supernatant of each specimen was removed to a glass insert in a vial using an appropriate pipette. d) The volume removed should equal 75% of the original specimen volume to avoid dislodging of or contamination with pelleted material. e) A "blank sample" was also prepared by using high purity water instead of the serum and following the exact same procedure.

5. Composite quality control (QC) sample generation. a) An equal volume of each sample was removed from each Eppendorf tube using an appropriate micropipette. b) The removed sample volumes were pooled to create a single QC. c) The composite QC sample is mixed thoroughly by inversion and vortexing and distributed among several vials.

Sample analysis by UHPLC-MS

Sample analysis was performed on an Acquity UPLC system (Waters Ltd. Elstree, U.K.) coupled to a Q-TOF Premier mass spectrometer (Waters Ltd., Manchester, U.K.). Mass spectra were acquired in both positive and negative ionisation electrospray modes.

The separation conditions have been previously established (2). Gradient elution was performed using a CSH C18 (1.7μm, 2.1 x 100mm) column (Waters Corporation, Milford, U.S.A.) kept at 55ºC. The injection volume was 5 and 10 μL for the positive and negative ion mode, respectively. The mobile phases consisted of 0.1% formic acid (v/v) and 10 mM ammonium formate in 60:40 (v:v) acetonitrile/water (A) and 0.1% formic acid (v/v) and 10 mM ammonium formate in 90:10 (v:v) IPA/acetonitrile (B) at a flow rate of 0.4 mL/min.

The MS parameters were set as follow: capillary voltage, 3 kV (ESI+) and 2.5 kV (ESI-); sample cone voltage, 30 V (ESI+) and 25 V (ESI-); source temperature 120ºC; desolvation temperature, 400ºC; desolvation gas flow, 800 L/h and cone gas flow, 25 L/h. For mass accuracy, a 0.2 ng/μL leucine enkephalin solution (m/z 556.2771 in positive ion mode, m/z 554.2615 in negative ion mode) at 20 μL/min was used as the lock mass. Data were collected in centroid mode with a scan range of 50-1200 m/z, with lock mass scans collected every 30 s and averaged over 3 scans to perform mass correction.

Masslynx v4.1 software (Waters, Manchester, U.K.) was used for data acquisition and visual inspection. Feature extraction was performed in XCMS (within R) (3). The centWave algorithm was used for peak picking, while the peak grouping step was performed with the density method. Lipid species tentative annotations were based on accurate *m/z* values, isotopic patterns and retention time, by matching the accurate mass of the molecular ion to reference spectra from LIPID MAPS (https://www.lipidmaps.org/) and human metabolome (HMDB; https://hmdb.ca/) publicly available databases. Some of the annotations had already been reported using the same chromatographic method (4).

Lipid mediators targeted analysis [5]

Sample preparation

Analytes were extracted using solid phase extraction (SPE) in 96-well plate format (Oasis MAX μElution plate from Waters (Milford, MA)). Briefly, 20 μL of IS working solution and 30 μL of 2% formic acid in water were added to 100 μL of serum sample. Samples were transferred into the SPE plate after conditioning, and the lipid mediators and PUFA were eluted with four times 25 μL of MeOH with 2% formic acid. The elution fraction was evaporated under nitrogen and the residues reconstituted in 120 μL of methanol/water 1:1 (v/v).

Sample analysis

Sample analysis was performed on an Acquity UPLC system (Waters Ltd. Elstree, U.K.) coupled to a TQ-S mass spectrometer (Waters Ltd., Manchester, U.K.) operated in the negative ionisation electrospray mode. Separation was performed with a Waters HSS T3 UPLC column (100 mm × 1 mm, 1.8 μm) column maintained at 40 °C. The mobile phases consisted of 0.1% formic acid in water (A) and 0.1% formic acid in acetonitrile (B), at a flow rate of 0.14 mL/min. The injection volume was 5 μL and a postcolumn infusion of acetonitrile with 37% formaldehyde 3:1 (v/v) at a flow rate of 5 μL/min was added. The source parameters were as follows: capillary voltage was set at 2.5 kV, cone voltage 10-40 V depending on the analyte, source temperature 150 °C, desolvation temperature 500 °C, desolvation

gas flow 900 L/h and cone gas flow 150 L/h. Analyses were performed in MRM mode, with dwell time, cone voltage and collision energy optimised for each analyte.

Peak detection, integration and quantification were performed using the TargetLynx within Masslynx 4.1 software.

Lipoprotein assays [6,7]

Serum stored at -80°C was thawed at 4°C then centrifuged at 12000 g at 4°C for 5 min, before 350 µL of each sample was transferred to an Eppendorf. This was then mixed with 350 µL of serum buffer (0.075 M NaH2PO4 - 5.32g NaH2PO4 dissolved in 380 mL ultrapure water, with 0.4 g 3-trimethyl-silyl-[2,2,3,3-2H4] propionic acid, sodium salt 98 atom % D, 5 mL of 4% NaN3 aqueous solution, and 100 mL of D2O sequentially added before pH was adjusted to 7.4 with HCl/ NaOH and the solution was made up to 500 mL with water). The mixture of sample and serum buffer was centrifuged at 12000g at 4°C for 5min, then 600 µL was loaded into a SampleJet NMR tube (Bruker, USA). All reagents were NMR grade, Sigma-Aldrich, USA.

Samples were run using the SampleJet loading system on a 600MHz Avance III NMR spectrometer (Bruker Biospin, USA) with a BBI 600 MHz 5-mm Z gradient probe NMR detector and automated tuning and matching unit (Bruker Biospin, USA). The temperature was calibrated so that serum samples were run at exactly 300 K. They were run using an optimised machine protocol described previously. Briefly, 1H NMR spectra were measured using a standardised water suppression pulse sequence, NOESY-presat, as well as by a standardised Carr-Purcell-Meiboom-Gill (CPMG) spin-echo sequence with presaturation, and Bruker J-resolved pulse sequence and diffiusion-filtered sequence 303. The spectra generated by these standardised sequences was then be analysed using Bruker's propriety B.I. Methods Package 2.0 which quantifies a lipoprotein subclass panel with 114 parameters (B.I. LISA). Analytes quantified by B.I. LISA are listed below. These metabolites, molecules and lipoproteins can be analysed directly. The spectra (e.g. CPMG) can be also be analysed, and discriminatory peaks identified if possible.

Abbreviations: VLDL = Very Low Density Lipoprotein; IDL = Intermediate Density Lipoprotein; LDL = Low Density Lipoprotein; HDL = High Density Lipoprotein. Subfractions 1-6 are numbered according to increasing density, as reproduced below.

Densities (in kg/L) of Lipoprotein Main Fractions:

VLDL	IDL	LDL	HDL
0.950 - 1.006	1.006 - 1.019	1.019 - 1.063	1.063 - 1.210

Density of the Very Low Density Lipoprotein Subfractions:

5 subfractions VLDL-1 ... VLDL-5, numbering according to increasing density.
Subfractions properties are specified in [1]

Densities (in kg/L) of Low Density Lipoprotein Subfractions:

LDL-1	LDL-2	LDL-3	LDL-4	LDL-5	LDL-6
1.019 - 1.031	1.031 - 1.034	1.034 - 1.037	1.037 - 1.040	1.040 - 1.044	1.044 - 1.063

Densities (in kg/L) of High Density Lipoprotein Subfractions:

HDL-1	HDL-2	HDL-3	HDL-4
1.063 - 1.100	1.100 - 1.112	1.112 - 1.125	1.125 - 1.210

- Cholesterol
- LDL-cholesterol
- HDL-cholesterol
- Apo-A1

- Apo-A2
- Apo-B100
- LDL-cholesterol/ HDL-cholesterol
- Apo-B100/ Apo-A1
- Total Particle Number
- VLDL Particle Number
- IDL Particle Number
- LDL Particle Number
- LDL-1 Particle Number
- LDL-2 Particle Number
- LDL-3 Particle Number
- LDL-4 Particle Number
- LDL-5 Particle Number
- LDL-6 Particle Number
- VLDL triglycerides
- IDL triglycerides
- LDL triglycerides
- HDL triglycerides
- VLDL cholesterol
- IDL cholesterol
- LDL cholesterol
- HDL cholesterol
- VLDL free cholesterol
- IDL free cholesterol
- LDL free cholesterol
- HDL free cholesterol
- VLDL phospholipids
- IDL phospholipids
- LDL phospholipids
- HDL phospholipids
- HDL Apo-A1
- HDL Apo-A2
- VLDL Apo-B
- IDL Apo-B
- LDL Apo-B
- VLDL-1 triglyceride
- VLDL-2 triglyceride
- VLDL-3 triglyceride
- VLDL-4 triglyceride
- VLDL-5 triglyceride
- VLDL-1 cholesterol
- VLDL-2 cholesterol
- VLDL-3 cholesterol
- VLDL-4 cholesterol
- VLDL-5 cholesterol
- VLDL-1 free cholesterol
- VLDL-2 free cholesterol
- VLDL-3 free cholesterol

- VLDL-4 free cholesterol
- VLDL-5 free cholesterol
- VLDL-1 phospholipids
- VLDL-2 phospholipids
- VLDL-3 phospholipids
- VLDL-4 phospholipids
- VLDL-5 phospholipids
- LDL-1 triglyceride
- LDL-2 triglyceride
- LDL-3 triglyceride
- LDL-4 triglyceride
- LDL-5 triglyceride
- LDL-6 triglyceride
- LDL-1 cholesterol
- LDL-2 cholesterol
- LDL-3 cholesterol
- LDL-4 cholesterol
- LDL-5 cholesterol
- LDL-6 cholesterol
- LDL-1 free cholesterol
- LDL-2 free cholesterol
- LDL-3 free cholesterol
- LDL-4 free cholesterol
- LDL-5 free cholesterol
- LDL-6 free cholesterol
- LDL-1 phospholipids
- LDL-2 phospholipids
- LDL-3 phospholipids
- LDL-4 phospholipids
- LDL-5 phospholipids
- LDL-6 phospholipids
- LDL-1 Apo-B
- LDL-2 Apo-B
- LDL-3 Apo-B
- LDL-4 Apo-B
- LDL-5 Apo-B
- LDL-6 Apo-B
- HDL-1 triglyceride
- HDL-2 triglyceride
- HDL-3 triglyceride
- HDL-4 triglyceride
- HDL-1 cholesterol
- HDL-2 cholesterol
- HDL-3 cholesterol
- HDL-4 cholesterol
- HDL-1 free cholesterol
- HDL-2 free cholesterol
- HDL-3 free cholesterol

- HDL-4 free cholesterol
- HDL-1 phospholipids
- HDL-2 phospholipids
- HDL-3 phospholipids
- HDL-4 phospholipids
- HDL-1 Apo-A1
- HDL-2 Apo-A1
- HDL-3 Apo-A1
- HDL-4 Apo-A1
- HDL-1 Apo-A2
- HDL-2 Apo-A2
- HDL-3 Apo-A2
- HDL-4 Apo-A2

Cytokines, chemokines, immune and renal markers analyses

All reagents used in this method were purchased from MSD, Rockville, USA. Kits were combined in a customised manner. The kits purchased were: R-PLEX Human HGF Antibody Set; R-PLEX Human EGF Antibody Set; R-PLEX Human CD163 Antibody Set; R-PLEX Human IGF-1 Antibody Set; R-PLEX Human LBP Antibody Set; R-PLEX Human NGAL/LCN2 Antibody Set; R-PLEX Human TWEAK/TNFSF12 Antibody Set; R-PLEX Human Cystatin C Antibody Set; U-PLEX Custom Biomarker (hu) Assays (U-PLEX Human IFN-γ, U-PLEX Human IL-1α, U-PLEX Human IL-1β, U-PLEX Human IL-6, U-PLEX Human IL-10, U-PLEX Human IL-22, U-PLEX Human IL-23, U-PLEX Human TNF-α. U-PLEX Human VEGF-A, Open Spots – 1 [to bind R-PLEX antibody]); U-PLEX Custom Immuno-Oncology Grp 1 (hu) Assays (U-PLEX Human IL-1RA; U-PLEX Human IL-8; U-PLEX Human IL-18; U-PLEX Human PD1 (Epitope 2); U-PLEX Human PD-L1 (Epitope 1); Open Spots – 4). Addition 4-spot plates and Diluent 3, 10, 43 and 100 were purchased. Samples were run on 96-well plates, with eight-point calibration curves run in duplicate after serial dilution in 96 well plates using diluent 100. The 10-plex was run with neat sample, the 9-plex with 1:10 and the 3-plex with 1:3000 dilutions.

Statistical analyses

Principal components analysis (PCA) was performed to visualise any inherent clustering and identify outliers (SIMCA v16.0). OPLS-DA was performed to maximise modelling of class differences while minimising variability unrelated to class. The R^2 value was calculated to give a measure of the goodness-of-fit or amount of variability explained by the model. A cross-validated Q2 statistic (leave-one-out algorithm) was calculated as a quantitative measure of the predictability of the model for the Y variable. The cross-validated analysis of-variance (CV-ANOVA) statistic corresponds to a null-hypothesis of equal predictive residuals between the models under investigation ($p<0.05$ suggests the model is superior to one chosen at random). Nine hundred ninety-nine permutation tests in the OPLS-DA were

performed to validate the models. Area under the receiving operating systems (AUROC) were built to evaluate the performance of the model to predict condition. S-plot loadings (displaying correlation versus covariance of spectral variables) and variable importance of the projection (VIP) tests were used to determine the features contributing to class separation. Features with a VIP value ≥ 2 were analysed on raw chromatograms and mass spectra for annotation using cross analyses of LIPID MAPS (https://www.lipidmaps.org/) and human metabolome (HMDB; https://hmdb.ca/) online databases. If the OPLS-DA model was not valid (or a low number of targeted metabolites formed the basis of the method), a volcano plot based on log 2-fold-change (log2[FC]) as X axis and -log10 of P-value of t-test (-log10[P-value]) as Y axis was built. Thresholds of -0.5 and 0.5 of log2(FC) and 2 of -log10(P-value) were set to identify features significantly associated with the condition. These features were analysed on raw chromatograms and mass spectra as performed in those identified from OPLS-DA models. After annotation, univariable and stepwise multivariable logistic regression was performed. Lipids with $p \leq 0.01$ were entered in the multivariable analysis to mitigate the risk of model overfitting based on an appropriate balance between the number of events and the number of variables. Adjustments on MELD score to differentiate between sAH and cirrhosis first, and Lille score between 90-day survivors and non-survivors in the sAH group were performed as an internal control. To obtain a probability score ranging from 0 to 1, the R function obtained by the forward logistic regression function combining lipids with a p-value ≤ 0.05 was inserted in the following formula: $1/(1 + \mathrm{Exp}(-R))$. This approach has been previously described for the development of the Lille Model (8). Performance of lipids-based models were compared to reference scores using AUROC curves and the z test (9). Threshold of -1 and -1 of log2(FC) and 1 of -log10(P-value) was set for lipid mediators analyses to increase sensitivity as none reached -log10 (P-value) threshold of 2. Missing data were not inputted. T-test, one-way ANOVA/Kruskal-Wallis, Pearson/Spearman's correlations and AUROC were calculated with NCSS v2022 (NCSS, LLC) or GraphPad Prism v9.0 (GraphPad Software, San Diego, CA).

Supplementary figures

Supplementary figure 1

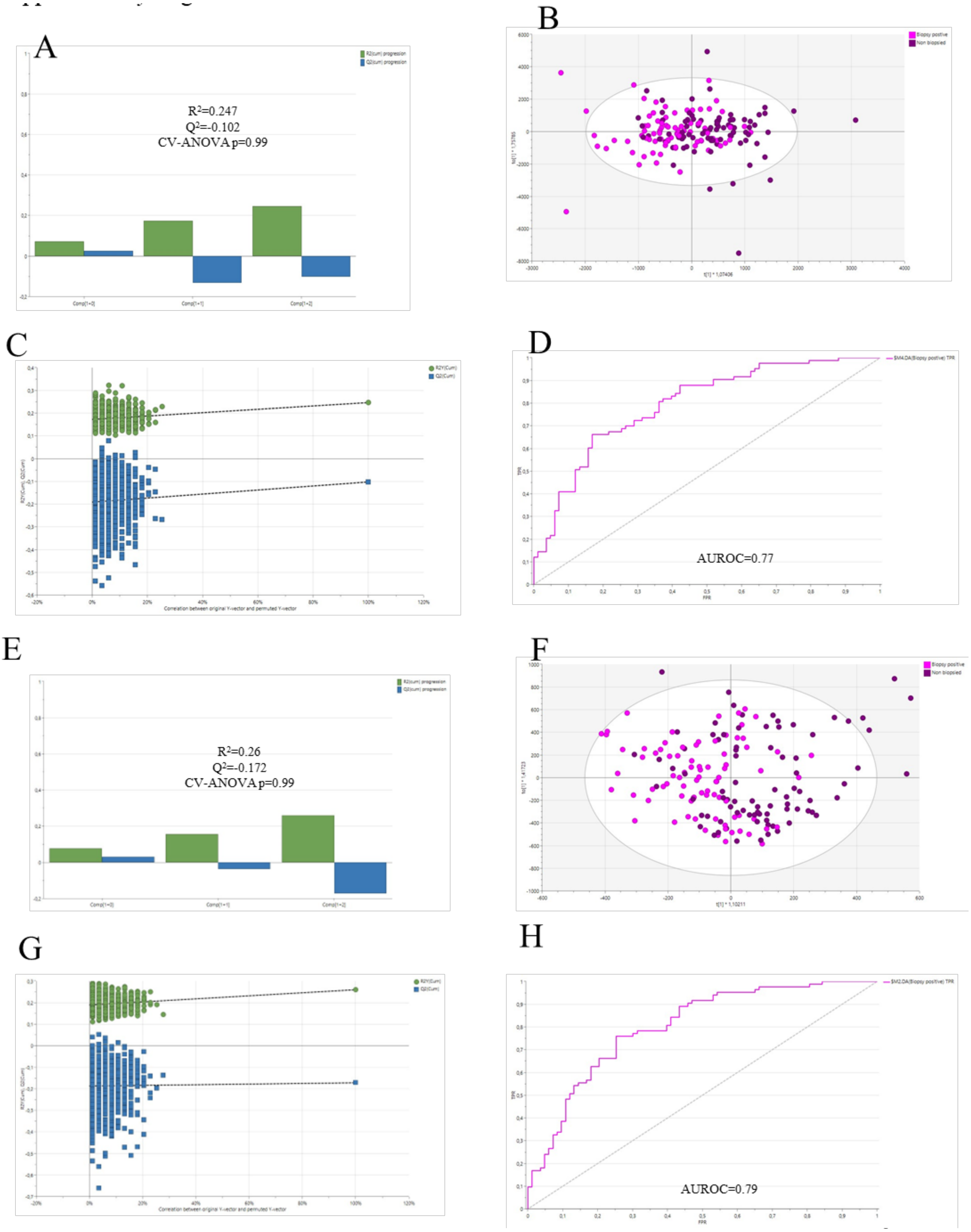

Legend to Fig. S1: Analyses of untargeted lipidomics between patients with sAH with positive features of sAH (n=76) and patients who did not undergo liver biopsy (n=83). A. Summary of fit of the OPLS-DA model in positive ionisation mode with 1+2+0 component: R^2=0.247, Q^2= -0.102, CV-ANOVA p=0.99. B. Plot of the positive ionisation mode model (biopsy positive patients in rose, non-biopsied patients in purple). C. Permutation test demonstrating the non-validity of the positive ionisation mode model. D. AUROC of the positive ionisation mode model. E Summary of fit of the model in negative ionisation mode with 1+2+0 component: R^2=0.26, Q^2= -0.172, CV-ANOVA p=0.99. F. Plot of the negative ionisation mode model (biopsy positive patients in rose, non-biopsied patients in purple). G. Permutation test demonstrating the non-validity of the negative ionisation mode model. H. AUROC of the negative ionisation mode model.

Supplementary figure 2

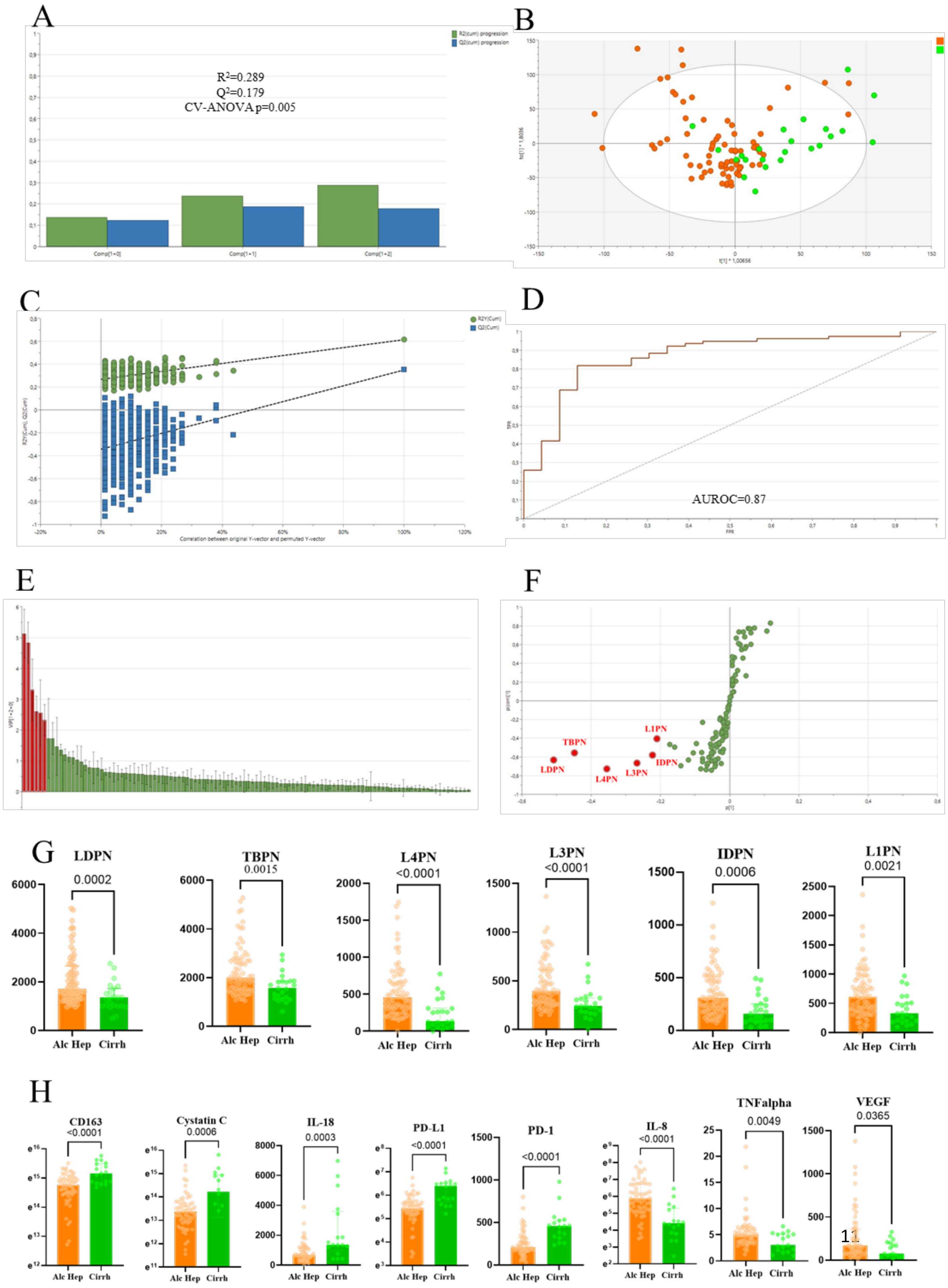

Legend to Fig. S2: Analyses of lipoprotein assay through 1H-NMR spectroscopy in patients with sAH (n=78) matched to patients with cirrhosis (n=23) on bilirubin level A. Summary of fit of the model in positive ionisation mode with 1+2+0 component: R^2=0.289, Q^2= 0.179, CV-ANOVA=0.005. B. Plot of the model with patients with sAH in orange and patients with DC in green. C. Permutation test demonstrating the non-validity of the model. D. AUROC of the model. E VIP plot of the OPLS-DA model: each bar in red represents a variable with VIP value ≥ 2. F. S-plot of the positive ionisation mode model: each variable is plotted; variables in red are those with VIP value ≥ 2. G. Univariate analyses of the six lipoproteins with a VIP value ≥ 2. H. Univariate analyses of the eight cytokines with different concentrations between patients with sAH and patients with DC.

Supplementary figure 3

A

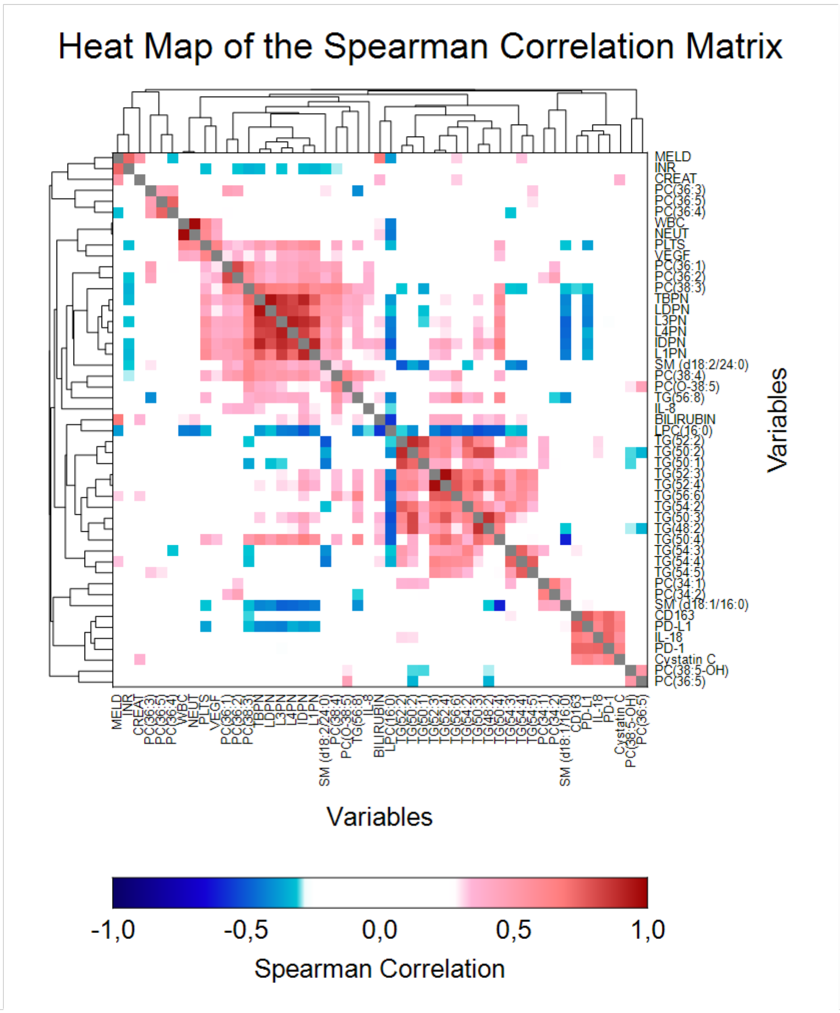

B

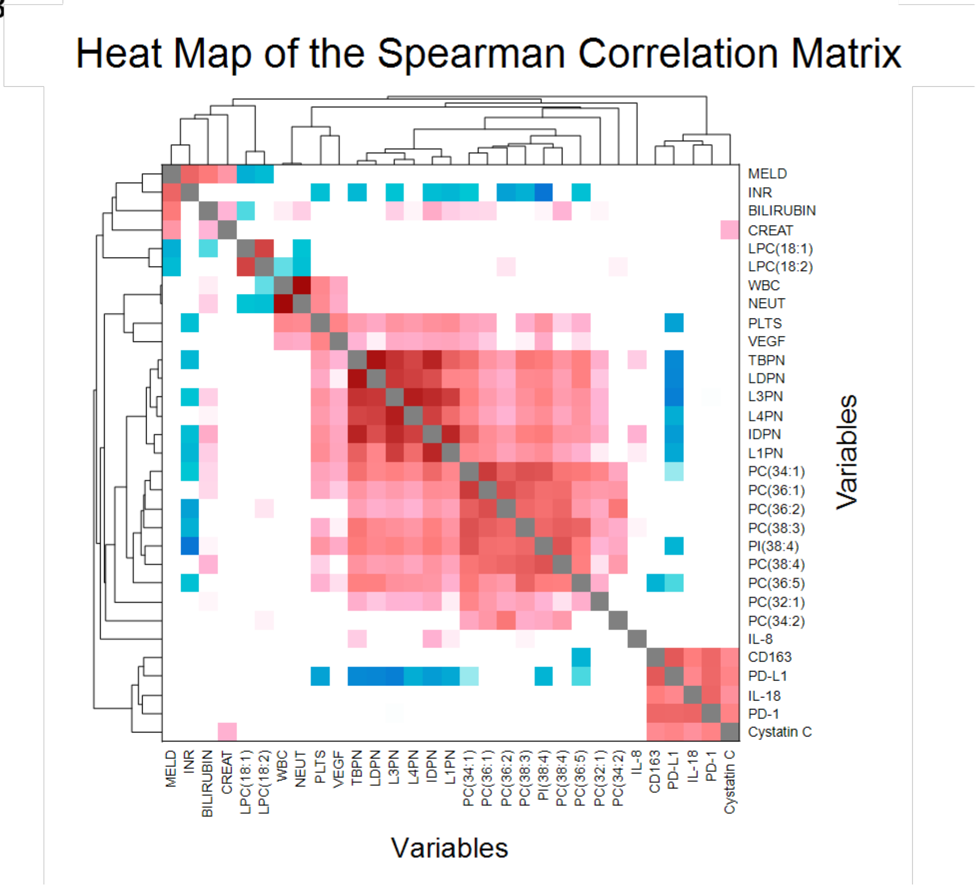

Legend to Fig. S3: Correlation matrix in in patients with sAH (n=78) matched to patients with DC (n=23) on bilirubin level based on Spearman's test between A. clinical, laboratory, lipids identified in positive ionisation mode with a VIP value ≥ 2, lipoproteins and cytokines identified in Fig. S2. B. clinical, laboratory, lipids identified in negative ionisation mode with a VIP value ≥ 2, lipoproteins and cytokines identified in Fig. S2.

Supplementary figure 4

A

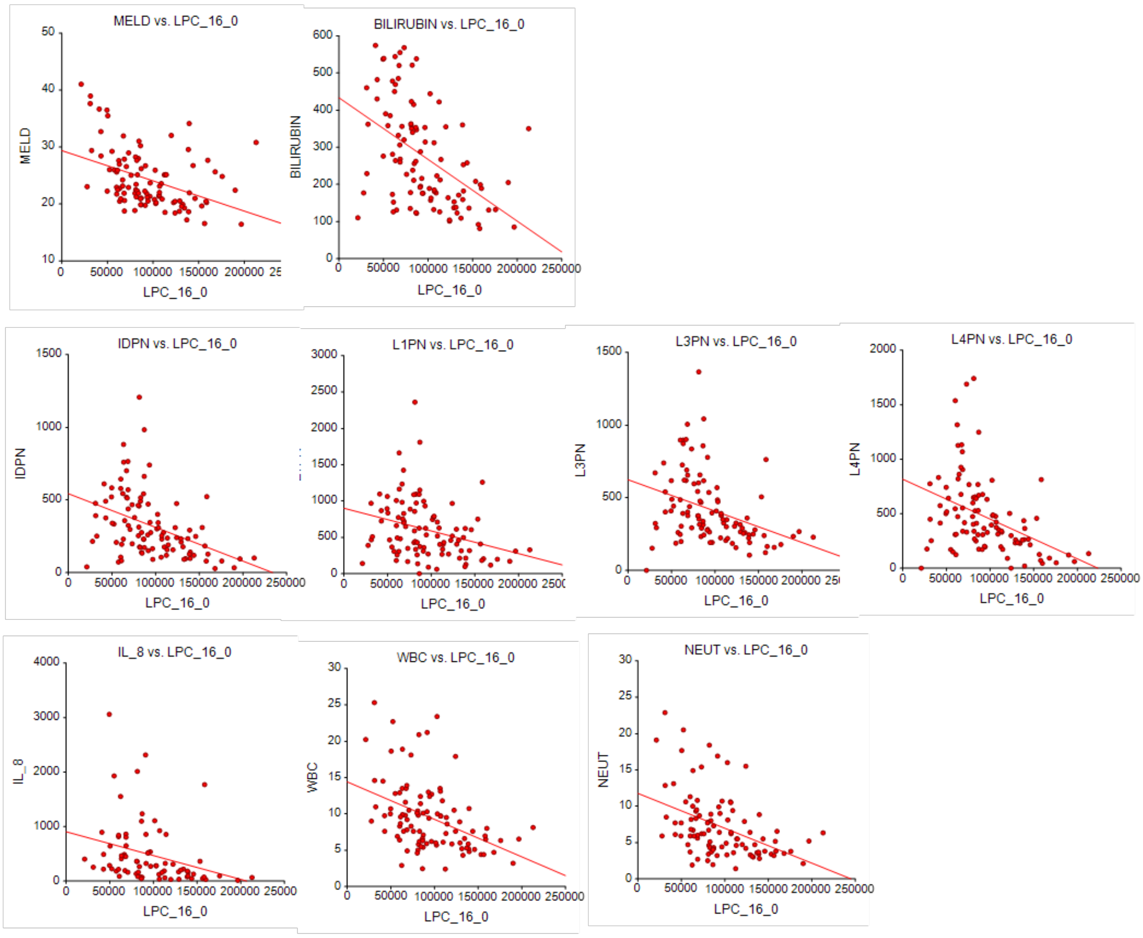

B

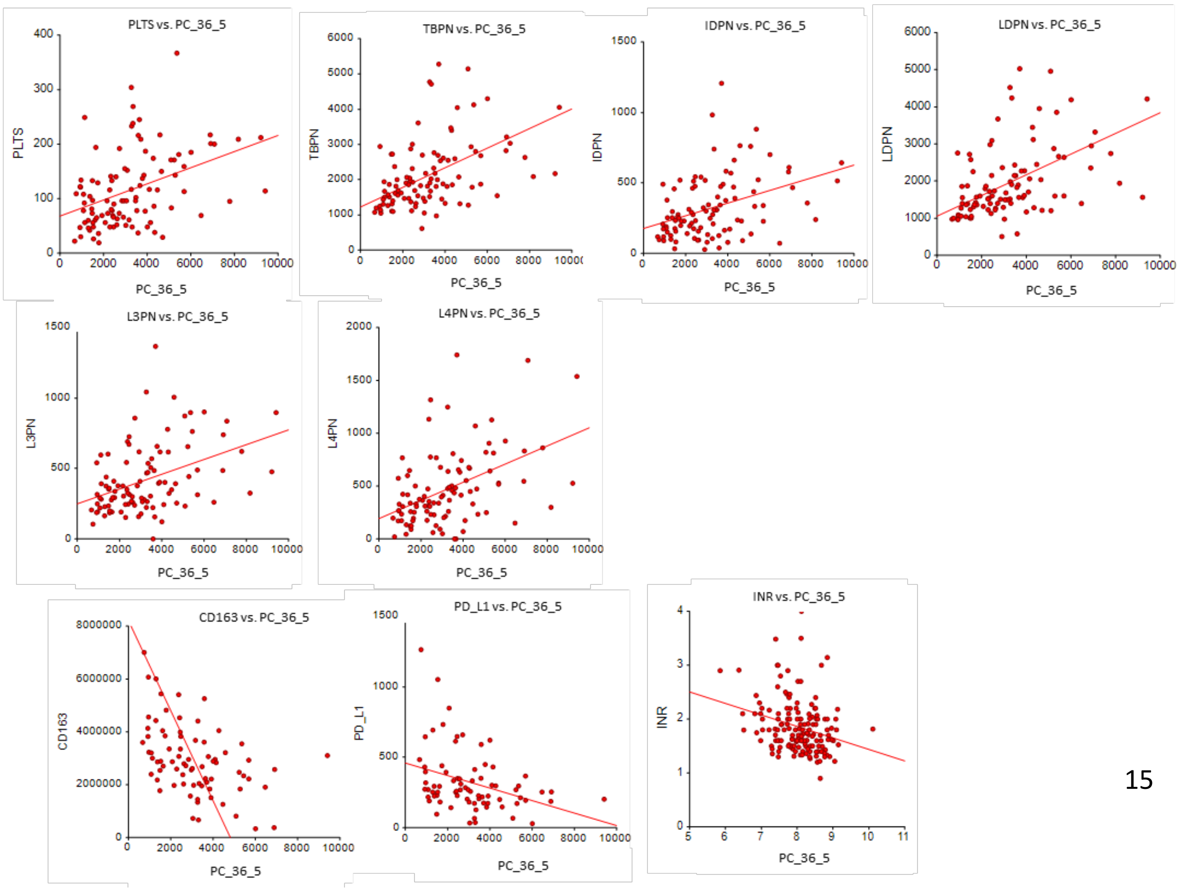

Legend to Fig. S4. A. Correlation plots of LPC(16:0) with MELD, bilirubin level, IDPN, L1PN, L3PN, L4PN, IL-8, WBC count and neutrophils counts (Spearman correlation coefficient and p value) in patients with sAH (n=78) matched to patients with cirrhosis (n=23) on bilirubin level. B. Correlation plots of PC(36:5) with platelet counts, TBPN, IDPN, LDPN, L3PN, L4PN, CD-163, PD-L1 levels and INR (Spearman correlation coefficient and p value) in patients with sAH (n=78) matched to patients with cirrhosis (n=23) on bilirubin level.

Supplementary figure 5

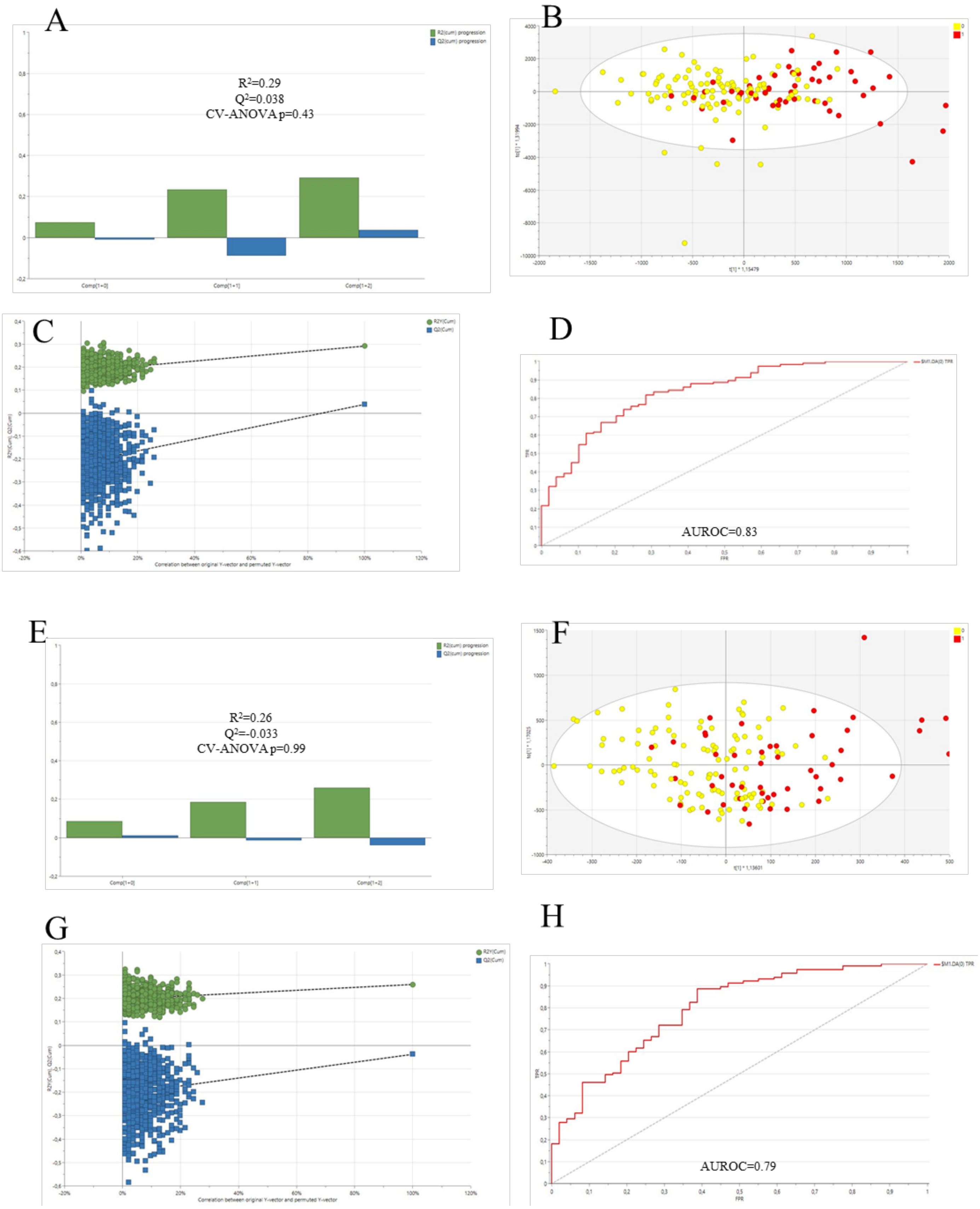

Legend to Fig. S5. A. summary of fit of the three-component model in OPLS-DA to differentiate between D90 survivors and non survivors in positive ionisation mode. B. Scores plot of the model in positive ionisation mode: each dot representing the model in one patient; D90-survivors in yellow, D90 non-survivors in red. C. Permutation test of the model in positive ionisation mode D. AUROC using the discriminant variables of the OPLS-DA model in positive ionisation mode. E. summary of fit of the three-component model in OPLS-DA to differentiate between D90 survivors and non survivors in negative ionisation mode. F Scores plot of the model in negative ionisation mode: each dot representing the model in one patient; D90-survivors in yellow, D90 non-survivors in red. G. Permutation test of the model in negative ionisation mode H. AUROC using the discriminant variables of the OPLS-DA model in negative ionisation mode.

Supplementary figure 6

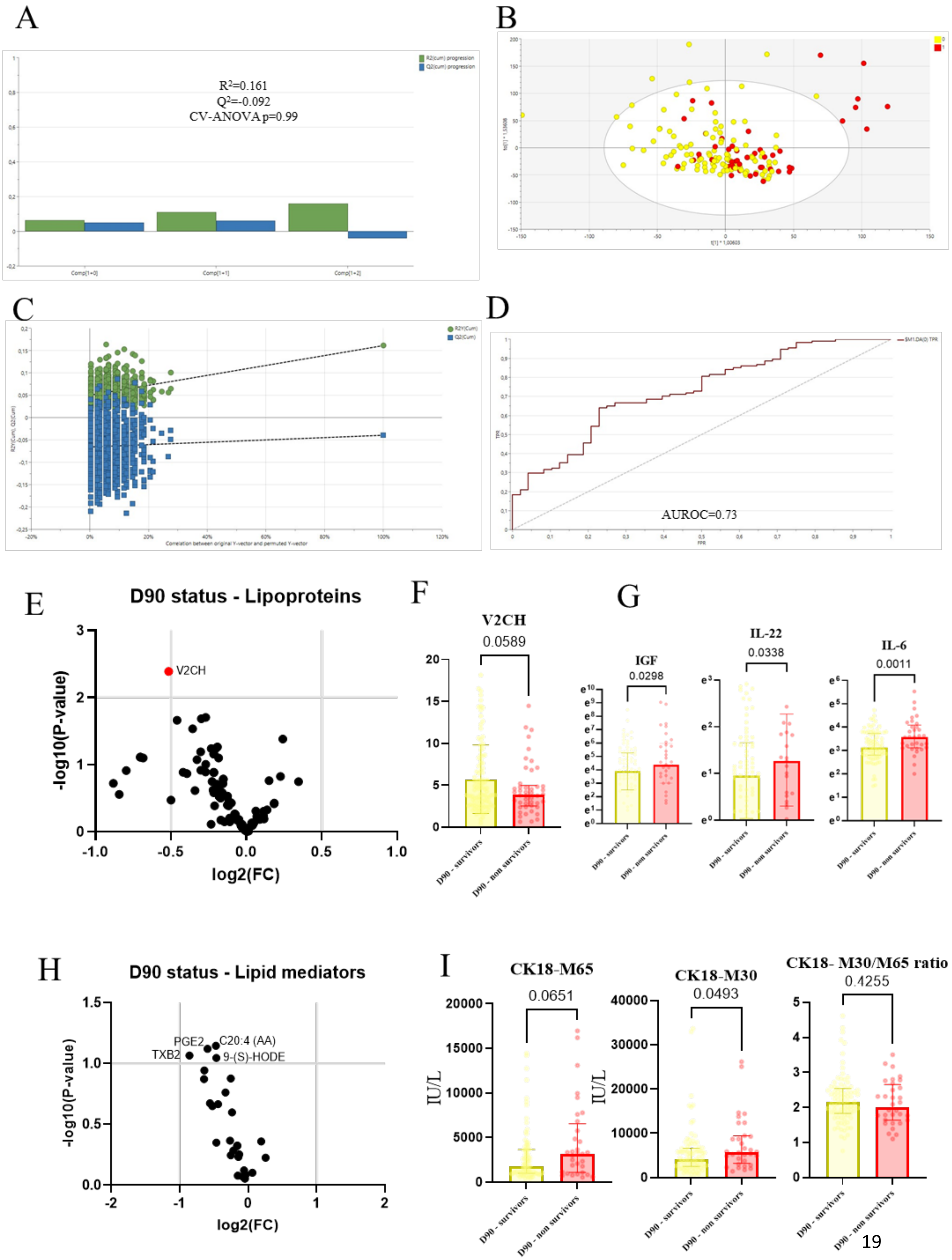

Legend to Fig. S6: Analyses restricted to patients with sAH based on status on day 90 (survivors n=106; non-survivors n=51). A. analyses of targeted lipoproteomics: summary of fit of the OPLS-DA model in positive ionisation mode with 1+2+0 component: R^2=0.161, Q^2=-0.092, CV-ANOVA p=0.99. B. Scores plot: each dot representing one patient; survivors in yellow, non-survivors in red. C. Permutation test demonstrating the non-validity of the model. D. AUROC of the model. E. Volcano plot of each lipoprotein with respect to the status at 90 days (survivors vs. non-survivors). F. Univariable analysis of V2CH levels with respect to the status at 90 days (survivors vs. non-survivors). G. Univariable analyses of cytokines differentiating between survivors vs. non-survivors at 90 days. H. Volcano plot of each lipid mediators with respect to the status at 90 days (survivors vs. non-survivors). I. Univariable analyses of cytokeratin 18 M65 and M30 fragments levels and their ratio with respect to status at 90 days (survivors vs. non-survivors)**.**

Supplementary figure 7

A

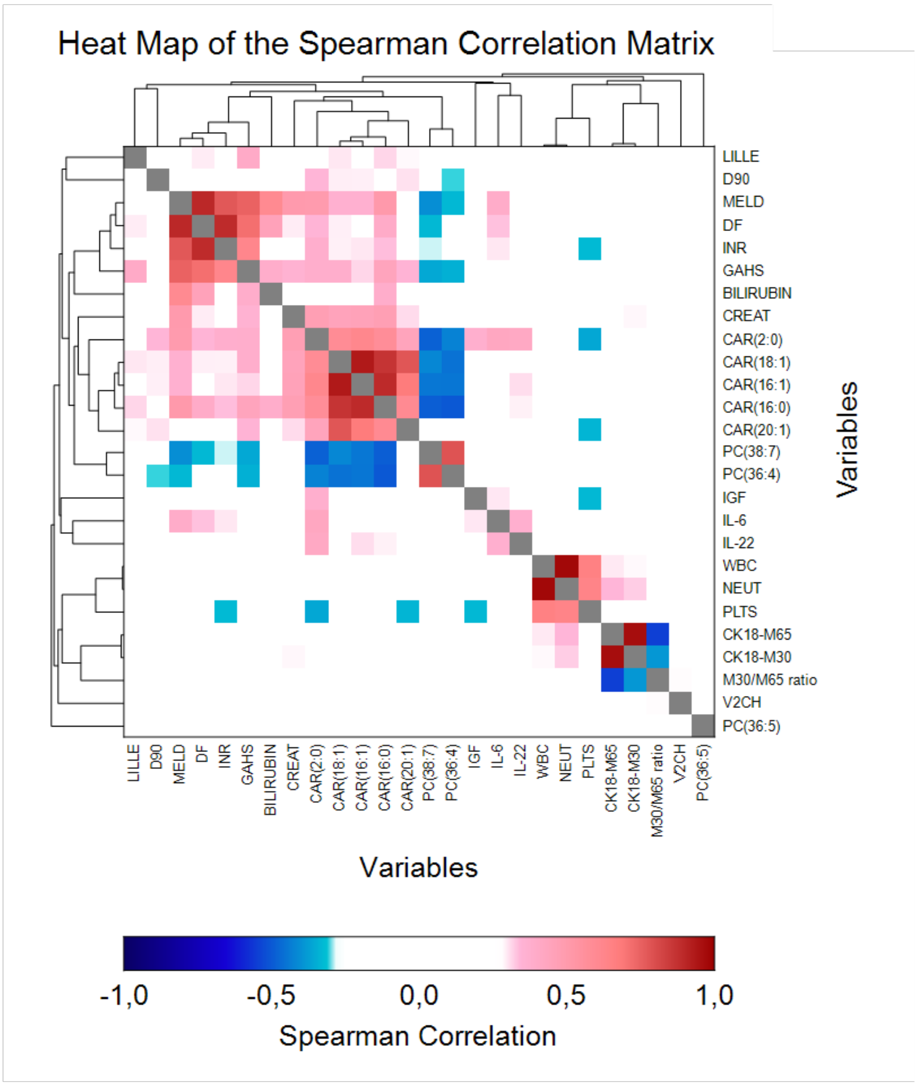

B

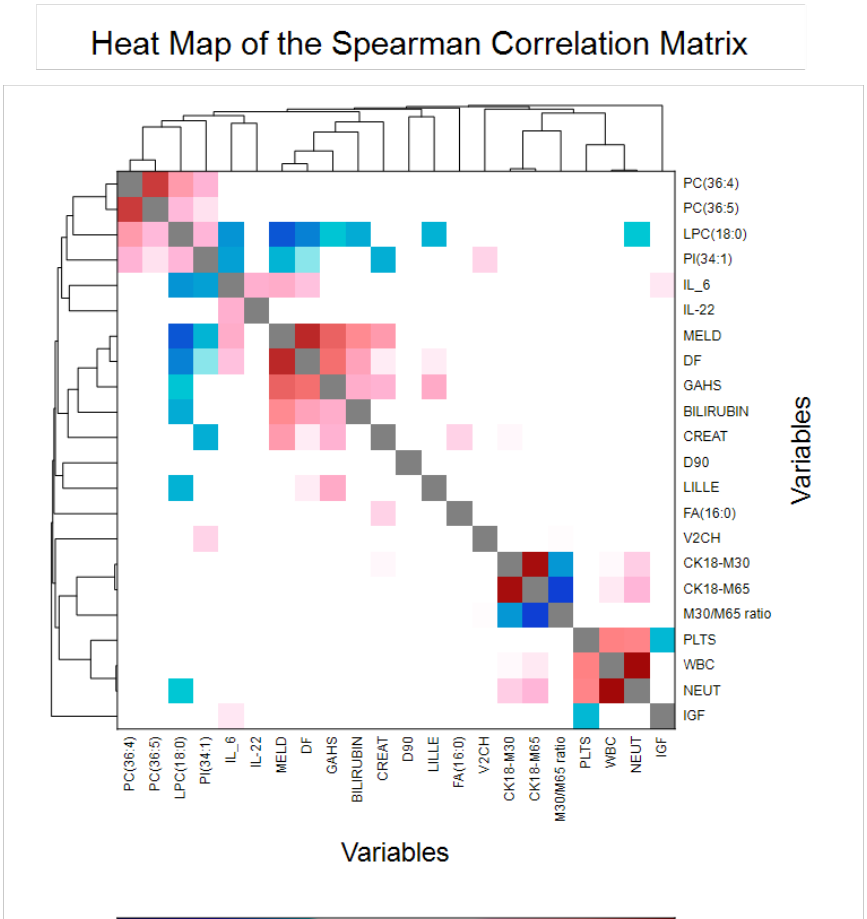

Legend to Fig. S7: Correlation matrix in in patients with sAH included in the final analyses (n=159) based on Spearman's test between A. clinical, laboratory, lipids annotated in positive ionisation mode on volcano plots (Figure 4A), lipoproteins and cytokines identified in Figure 5E-G and CK-18 M65 and M30 fragments and their ratio. B. clinical, laboratory, lipids identified in negative ionisation mode on volcano plots (Figure 4D), lipoproteins and cytokines identified in Figure 5E-G and CK-18 M65 and M30 fragments and their ratio.

Supplementary figure 8

A

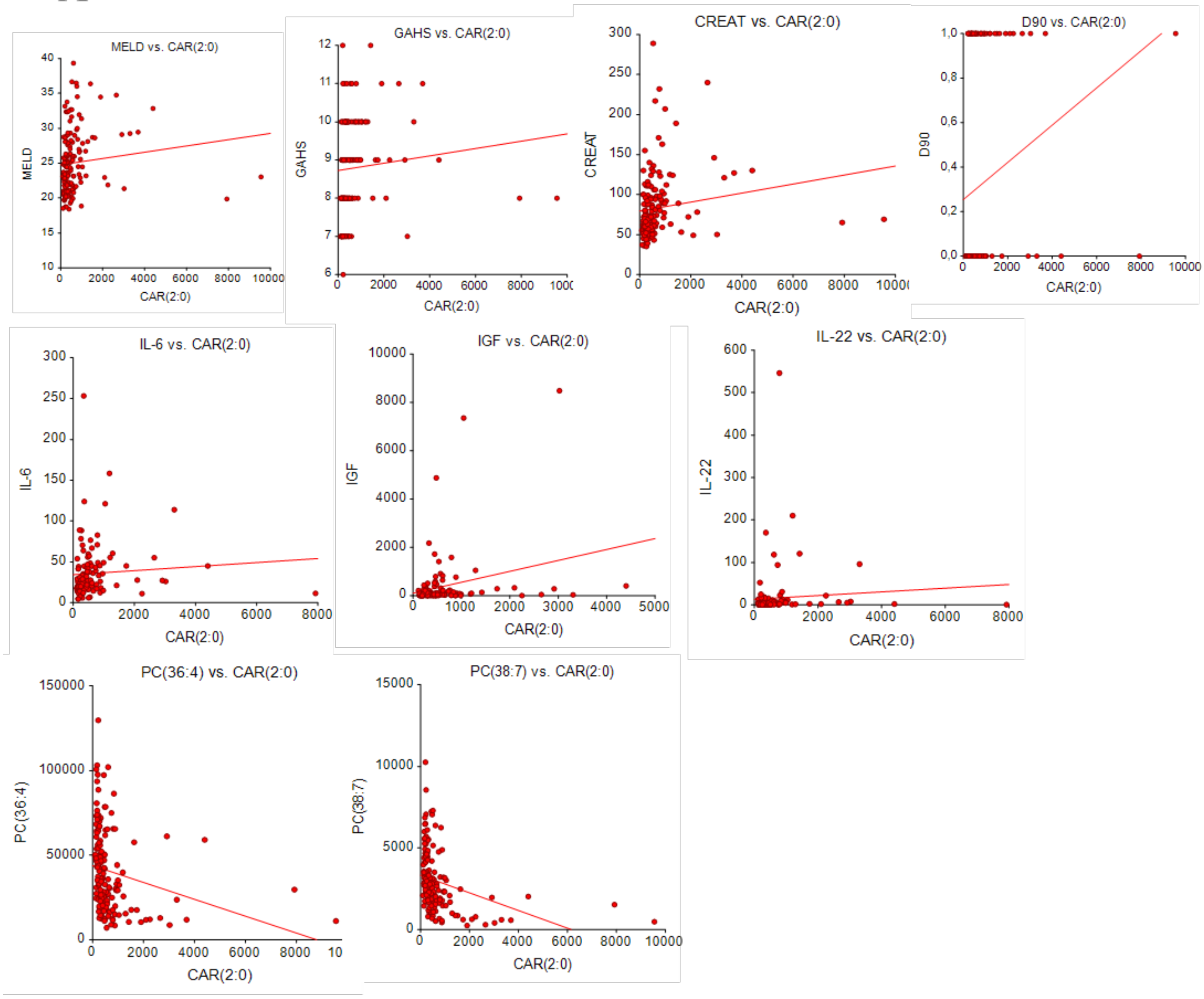

B

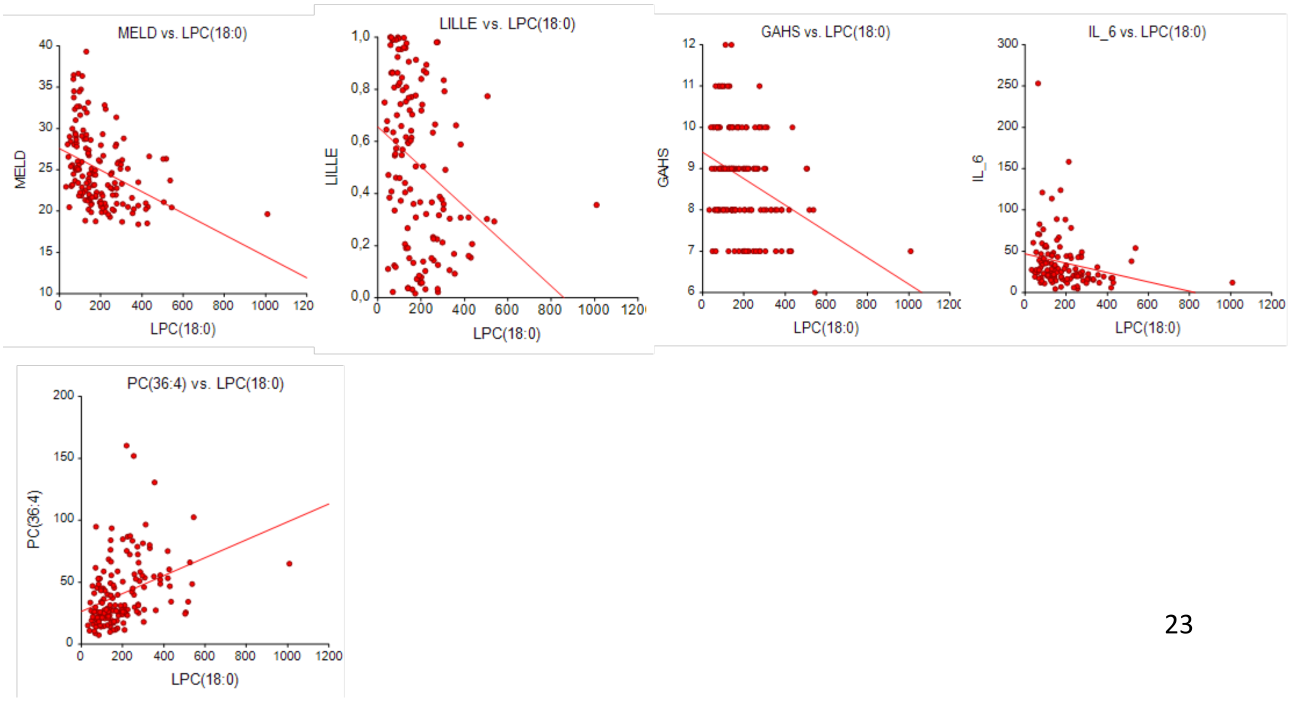

Legend to Fig. S8. Correlation plots of CAR(2:0) with MELD, Glasgow score, creatinine, D90 status, IL-6, IGF, IL-22, PC(36:4), PC(38:7) (Spearman correlation coefficient and p value) in patients with sAH (n=159). B. Correlation plots of LPC(18:0) with MELD, Lille score, Glasgow score, IL-6 and PC(36:4) (Spearman correlation coefficient and p value) in patients with sAH (n=159).

Supplementary Figure 9.

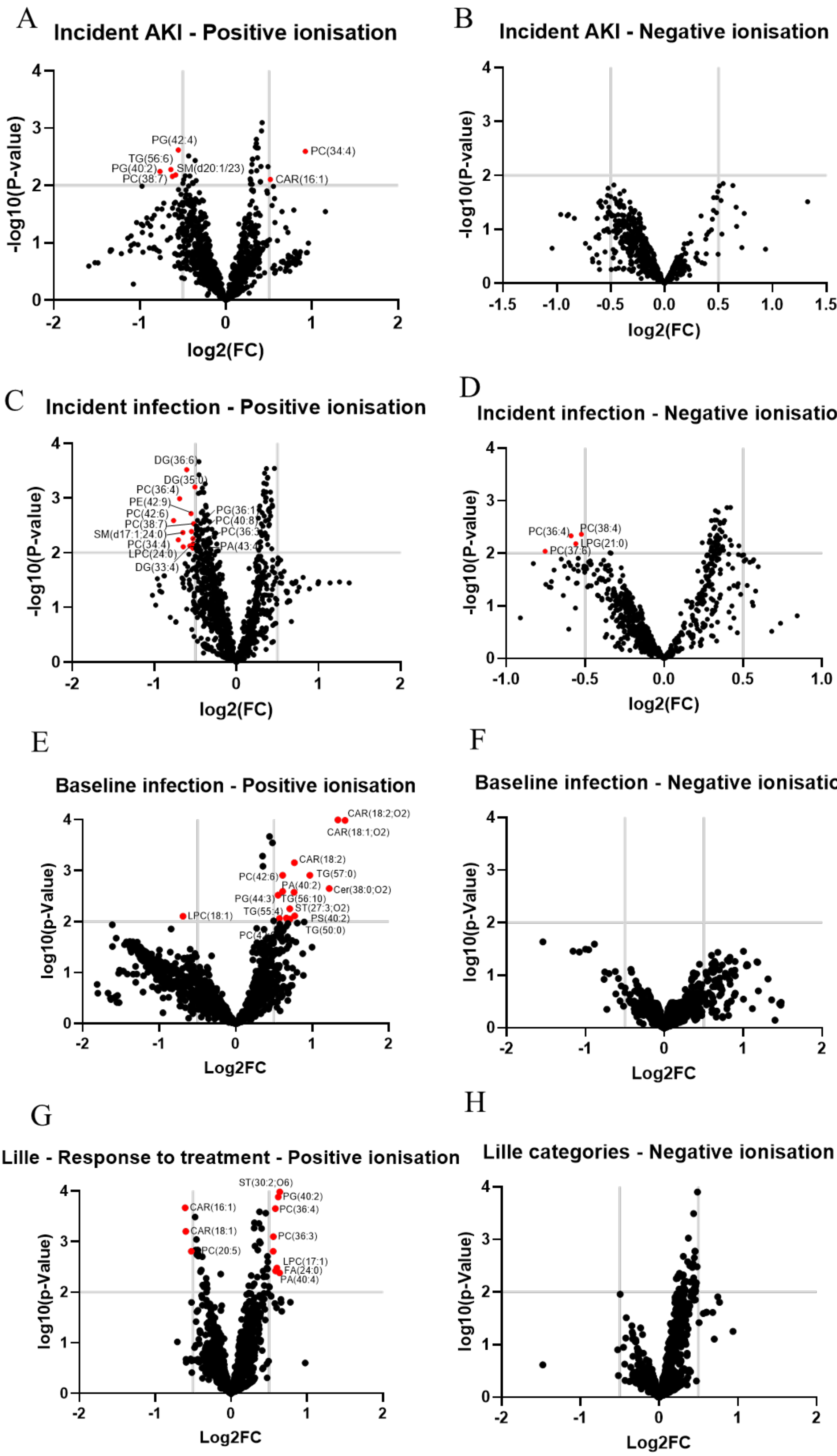

Legend to Fig. S9. A and B. Plots of each peak of positive (A) and negative (B) ionisation modes with respect to incident acute kidney injury (AKI) status: red plot identifying peaks above -0.5;0.5 log2(FC) threshold and 2 -log10(P-value) threshold that were further annotated based on raw chromatograms. C and D. Plots of each peak of positive (A) and negative (B) ionisation modes with respect to incident infection status: red plot identifying peaks above -0.5;0.5 log2(FC) threshold and 2 -log10(P-value) threshold that were further annotated based on raw chromatograms. D and E. Plots of each peak of positive and negative ionisation modes with respect to baseline infection status: red plot identifying peaks above -0.5;0.5 log2(FC) threshold and 2 -log10(P-value) threshold that were further annotated based on raw chromatograms. E and F. Plots of each peak of positive and negative ionisation modes with respect to response to treatment according to Lille model status: red plot identifying peaks above -0.5;0.5 log2(FC) threshold and 2 -log10(P-value) threshold that were further annotated based on raw chromatograms

Supplementary Figure 10.

A

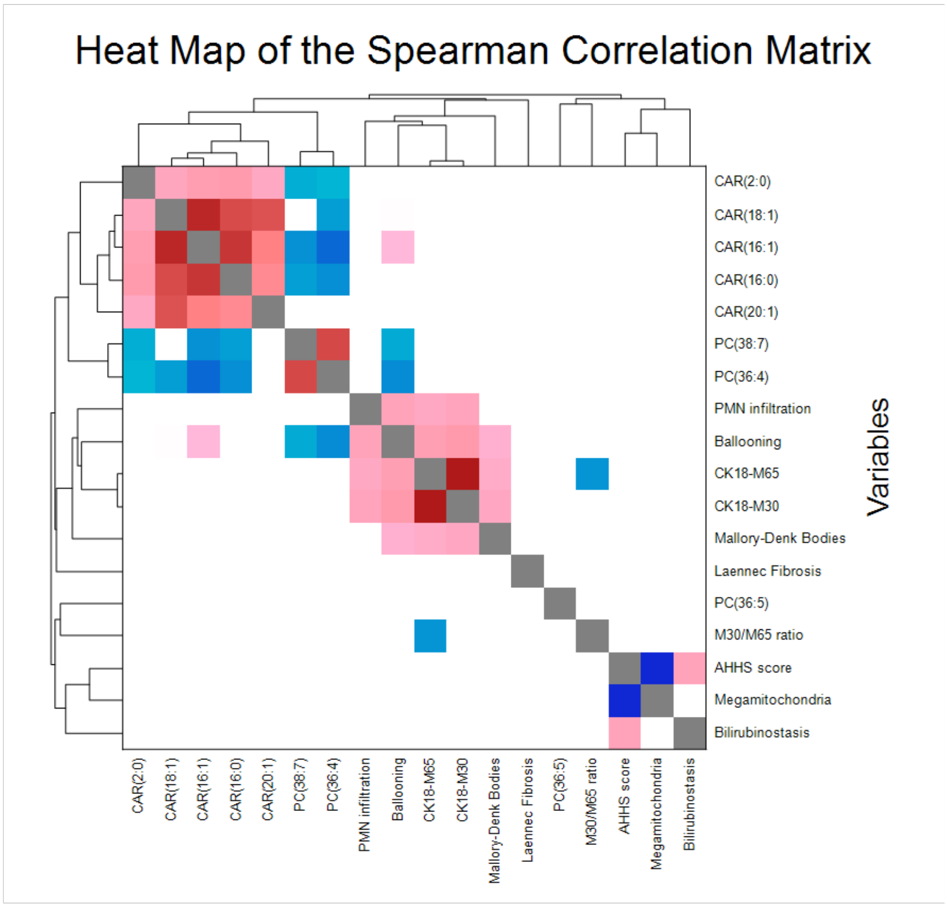

B

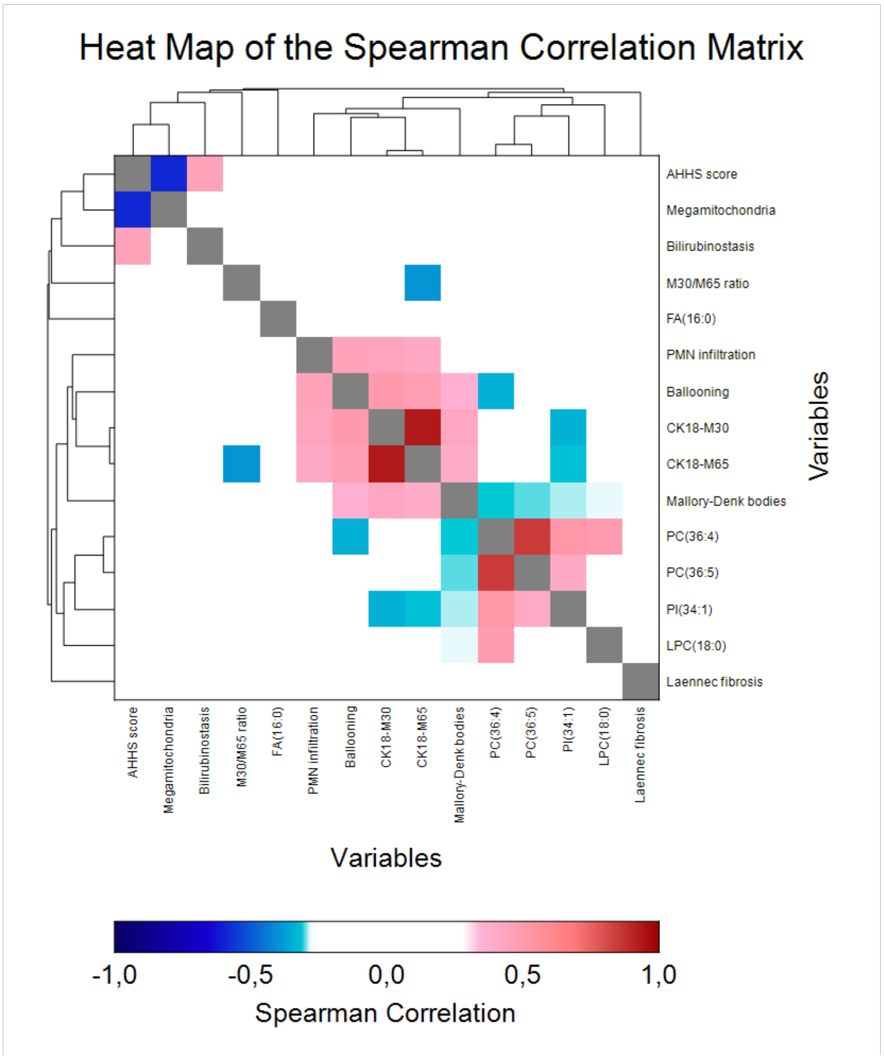

Legend to Fig. S10: Correlation matrix in in patients with sAH with positive biopsy (n=76) based on Spearman's test between A. lipids identified in positive ionisation mode on volcano plots (Figure 4A), CK-18 M65 and M30 fragments and their ratio and AHHSS score and group, fibrosis, neutrophils infiltration, ballooning, Mallory-Denk bodies, bilirubinostasis, and megamitochondria presence. Inflammation and steatosis as defined by AHHSS were not included as constant in the cohort with positive liver biopsy feature. B. clinical, laboratory, lipids identified in negative ionisation on volcano plots (Figure 4C), CK-18 M65 and M30 fragments and their ratio, AHHSS score and group, fibrosis, neutrophils infiltration, ballooning, Mallory-Denk bodies, bilirubinostasis, and megamitochondria presence. Inflammation and steatosis as defined by AHHSS were not included as constant in the cohort with positive liver biopsy feature.

Supplementary tables

Table S1

Characteristics of patients at time of sampling in patients who were biopsied with features of alcohol related hepatitis (n=76) and patients with clinical history of alcohol related hepatitis who did not underwent biopsy (n=83). Continuous and categorical variables expressed respectively in median (interquartile range) and N (percentages). Student's t-test or Mann-Whitney U test for quantitative variables or Chi-Square and Fisher exact tests for categorical variables were used as appropriate.

[redacted]			
**Characteristics**			
Age (years)	49.0 (42.8-56.7)	53.0 (43.8-57.8)	0.09
Sex (male)	52 (68.4)	51 (51.5)	0.61
BMI (kg/m^2)	25.4 (22.3-29.8)	26.1 (22.1-31.5)	0.39
Alcohol related liver disease	76 (100)	83(100)	0.99
Biopsy (yes)	76 (100)	-	-
**Laboratory**			
Leukocytes (G/l)	9.2 (6.2-12.3)	10.1 (7.6-13.5)	0.23
Neutrophils (G/l)	6.2 (4.0-9.4)	7.7 (5.5-10.6)	0.22
Hemoglobin (g/l)	108 (99.0-122.0)	111.0 (92.0-121.0)	0.33
Platelets (G/l)	137.0 (86.0-185.0)	113.0 (78.0-189.0)	0.72
INR	1.6 (1.5-2.0)	1.8 (1.6-2.1)	0.11
Bilirubin (mmol/l)	359.0 (260.0-511.0)	325.0 (227.0-433.0)	0.07
AST (IU)	132.0 (96.0-160.0)	136.0 (100.0-179.0)	0.13
ALP (IU)	179.0 (132.0-254.0)	183.0 (134.0-252.0)	0.92
Albumin (g/l)	24.0 (22.0-30.0)	24.0 (21.0-29.0)	0.21
Creatinine (mmol/l)	70.0 (59.0-102.0)	72.0 (52.0-96.0)	0.96
Urea (mmol/l)	3.4 (2.4-5.9)	3.7 (2.3-6.0)	0.46
Sodium (mmol/l)	136.0 (133.0-138.0)	132.0 (127.0-134.0)	<0.0001
**Scores**			
MELD	23.5 (20.9-26.7)	24.6 (21.9-28.1)	0.42
Maddrey DF	54.3 (40.1-74.5)	60.0 (56.8-77.7)	0.28
Lille	0.40 (0.2-0.7)	0.6 (0.3-0.8)	0.06
**Outcome**			
Death at 3 months	13 (17.1)	38 (46.0)	<0.0001

Table S2

Characteristics of patients at time of sampling (overall cohort, N=233; patients with alcohol related hepatitis n=159; patients with cirrhosis n=74). Continuous and categorical variables expressed respectively in median (interquartile range) and N (percentages). Mann-Whitney U test for quantitative variables or Chi-Square and Fisher exact tests for categorical variables were used as appropriate.

[redacted]				
**Characteristics**				
Age (years)	51.5 (43.6-58.6)	50.9 (43.1-57.6)	54.0 (45.0-61.8)	0.10
Sex (male)	160 (66.7)	109 (65.7)	51 (68.9)	0.61
BMI (kg/m^2)	25.4 (22.4-30.1)	26.0 (22.4-30.8)	24.0 (22.6-27.7)	0.02
Alcohol related liver disease	224 (93.3)	166 (100)	58 (78.4)	<0.0001
Biopsy (yes)	-	83 (50)	-	-
**Laboratory**				
Leukocytes (G/l)	8.2 (5.8-12.3)	9.5 (6.8-13.0)	6.3 (4.7-8.6)	<0.0001
Neutrophils (G/l)	5.7 (3.6-9.3)	6.6 (4.5-10.1)	3.6 (2.8-5.9)	0.0005
Hemoglobin (g/l)	109.0 (95.8-122.0)	109 (97.0-121.0)	106.0 (90.0-129.0)	0.68
Platelets (G/l)	117.0 (77.0-183.0)	119.0 (79.0-186.0)	109.0 (68.0-176.0)	0.32
INR	1.7 (1.4-2.0)	1.7 (1.5-2.0)	1.4 (1.2-1.9)	0.14
Bilirubin (µmol/l)	268.0 (123.0-422.0)	334.0 (231.0-459.0)	41.0 (21.0-118.0)	<0.0001
AST (IU)	100.0 (54.0-154.0)	134.0 (100.0-170.0)	47.0 (32.0-78.0)	0.0005
ALP (IU)	162.0 (116.0-237.0)	182.0 (133.0-253.0)	125.0 (83.0-166.0)	<0.0001
Albumin (g/l)	27.0 (22.0-33.0)	24.0 (21.0-29.0)	33.0 (25.0-36.0)	<0.0001
Creatinine (µmol/l)	72.0 (58.0-103.0)	71.0 (57.0-98.0)	74.0 (62.0-114.0)	0.05
Urea (mmol/l)	3.7 (2.5-6.6)	3.5 (2.4-5.9)	4.9 (3.0-9.3)	<0.0001
Sodium (mmol/l)	135.0 (131.0-138.0)	134.0 (129.0-137.0)	137.0 (134.0-140.0)	<0.0001
**Scores**				
MELD	22.9 (19.8-27.0)	24.2 (21.5-27.8)	15.0 (10.9-24.6)	<0.0001
Maddrey DF	56.0 (43.4-75.3)	56.0 (43.4-75.3)	-	-
Lille	0.5 (0.2-0.8)	0.5 (0.2-0.8)	-	-
**Outcome**				
Death at 3 months	62 (25.8)	52 (31.3)	10 (13.5)	0.004

Table S3

The 29 lipids putatively identified from the 74 features with a VIP value ≥ 2 in the positive ionisation mode.

PC(34:1)	$[M+H]^+$	2	760.587	7.2	11.8081
PC(34:2)	$[M+H]^+$	2	758.571	5.8	10.6465
SM (d18:1/16:0)	$[M+H]^+$	1	703.577	5.2	6.40203
TG(52:2)	$[M+NH_4]^+$	4	876.805	15.7	6.07929
TG(52:3)	$[M+NH_4]^+$	2	874.79	15.4	5.37885
PC(36:3)	$[M+H]^+$	2	784.588	6.0	5.06594
TG(54:3)	$[M+NH_4]^+$	3	902.821	15.7	4.82478
TG(54:4)	$[M+NH_4]^+$	4	900.806	15.5	4.74365
TG(54:5)	$[M+NH_4]^+$	5	898.79	15.2	4.52367
PC(36:1)	$[M+H]^+$	6	788.62	9.5	4.37981
TG(52:4)	$[M+NH_4]^+$	4	872.774	15.2	4.21215
PC(36:2)	$[M+H]^+$	2	786.602	7.7	3.97121
TG(50:2)	$[M+NH_4]^+$	4	848.774	15.4	3.54993
PC(38:3)	$[M+H]^+$	3	812.62	8.3	3.42408
TG(50:1)	$[M+NH_4]^+$	2	850.79	15.7	3.42084
PC(O-38:5)	$[M+H]^+$	1	794.608	6.7	3.39092
SM (d18:2/24:0)	$[M+H]^+$	4	813.688	11.9	3.39012
TG(54:2)	$[M+NH_4]^+$	3	904.837	16.0	3.369
TG(50:3)	$[M+NH_4]^+$	5	846.758	15.1	3.34927
PC(36:5)	$[M+H]^+$	1	780.557	4.7	3.25165
PC(36:4)	$[M+H]^+$	2	782.572	4.9	2.99232
TG(48:2)	$[M+NH_4]^+$	4	820.743	15.1	2.81332
PC(38:4)	$[M+H]^+$	5	810.604	7.4	2.62413
LPC(16:0)	$[M+H]^+$	1	496.34	1.1	2.61124
PC(38:5-OH)	$[M+H]^+$	3	808.589	6.2	2.52024
TG(50:4)	$[M+NH_4]^+$	4	844.742	14.9	2.29547
TG(56:6)	$[M+NH_4]^+$	6	924.806	15.3	2.2495
TG(56:8)	$[M+NH_4]^+$	4	920.775	14.9	2.1741
PC(36:5)	$[M+H]^+$	1	766.576	6.3	2.0883

Table S4

The 11 lipids putatively identified from the 40 features with a VIP value ≥ 2 in the negative ionisation mode.

PC(34:1)	$[2M+FA]^-$	4	1564.160	7.2	2.01514
PI(38:4)	$[M-H]^-$	8	885.552	5.5	3.37548
PC(38:3)	$[M+FA-H]^-$	11	856.611	8.3	2.91123
PC(38:4)	$[M-H]^-$	4	855.597	7.4	2.65136
PC(36:1)	$[M+FA]^-$	8	832.607	9.6	3.97649
PC(36:2)	$[M+FA]^-$	12	831.598	7.7	3.15473
PC(36:5)	$[M+FA]^-$	7	824.544	4.8	2.26431
PC(34:2)	$[M+FA]^-$	2	802.548	5.9	4.78334
PC(32:1)	$[M+FA]^-$	9	776.544	5.6	3.17116
LPC(18:1)	$[M+FA]^-$	0	566.326	1.2	2.2196
LPC(18:2)	$[M+FA]^-$	8	564.327	1.0	2.16906

Table S5

The eight lipids putatively identified above the log2(FC) and -log10(P-value) thresholds on the volcano plot in positive ionisation mode to differentiate between D90 survivors vs. non survivors.

CAR(2:0)	$[M+H]^+$	2	204.121	0.5	0.98	2.32
PC(36:5)	$[M+H]^+$	2	780.557	4.1	-0.72	2.41
CAR(18:1)	$[M+H]^+$	2	426.357	1.2	0.57	3.17
PC(38:7)	$[M+H]^+$	2	804.556	4.9	-0.69	4.40
CAR(16:1)	$[M+H]^+$	5	398.325	0.9	0.61	4.67
CAR(16:0)	$[M+H]^+$	5	401.345	1.2	0.52	4.88
CAR(20:1)	$[M+H]^+$	2	454.389	1.6	0.53	4.96
PC(36:4)	$[M+H]^+$	2	782.572	4.9	-0.83	5.24

Table S6

The 13 lipids putatively identified above the log2(FC) and -log10(P-value) thresholds on the volcano plot in negative ionisation mode to differentiate between D90 survivors vs. non survivors.

PC(36:4)	$[M+FA-H]^-$	11	829.569	4.9	-0.62	3.43
FA(16:0)	$[M-H]^-$	11	255.232	2.5	0.57	2.98
LPC(18:0)	$[M+FA-H]^-$	1	508.340	1.6	-0.51	2.41
PI(34:1)	$[M-H]^-$	4	835.534	5.4	-0.50	2.28
PC(36:5)	$[M+FA-H]^-$	3	824.547	4.2	-0.64	2.14

Table S7

Univariable and multivariable logistic regression analyses of lipids species associated with D90 status in negative ionisation mode. Effect size calculated per standard deviation increase.

[redacted]						
PC(36:4)	0.42	0.25-0.69	0.0008	0.38<br>0.48*	0.15-0.89<br>0.27-0.88*	0.03<br>0.02*
FA(16:0)	1.58	1.13-2.21	0.009	1.53<br>1.34	1.08-2.19<br>0.58-3.08*	0.02<br>0.32*
LPC(18:0)	0.70	0.47-1.06	0.09			
PI(34:1)	0.52	0.33-0.84	0.006	0.67	0.41-1.09	0.09
PC(36:5)	0.56	0.36-0.88	0.01	1.09	0.71-1.67	0.69

*after adjustement on Lille score (12.69, 2.93-54.98, p=0.0009)

Supplementary references

[1] Sarafian, M. H.; Gaudin, M.; Lewis, M. R.; Martin, F. P.; Holmes, E.; Nicholson, J. K.; Dumas, M. E. Objective set of criteria for optimization of sample preparation procedures for ultra-high throughput untargeted blood plasma lipid profiling by ultra performance liquid chromatography-mass spectrometry. Anal Chem 2014, 86 (12), 5766-5774. DOI: 10.1021/ac500317c

[2] Isaac, G.; Mc Donald, S.; Astaritra, G. Lipid Separation using UPLC with Charged Surface Hybrid Technology. Waters Application Note 720004107en; 2011

[3] Smith, C. A.; Want, E. J.; O'Maille, G.; Abagyan, R.; Siuzdak, G., XCMS: processing mass spectrometry data for metabolite profiling using nonlinear peak alignment, matching, and identification. Anal Chem 2006, 78 (3), 779-87

[4] Vorkas PA, Shalhoub J, Isaac G, Want EJ, Nicholson JK, Holmes E, Davies AH. Metabolic phenotyping of atherosclerotic plaques reveals latent associations between free cholesterol and ceramide metabolism in atherogenesis. J Proteome Res. 2015 Mar 6;14(3):1389-99. doi: 10.1021/pr5009898. Epub 2015 Feb 23. PMID: 25565173.

[5] Development and Validation of a High-Throughput Ultrahigh-Performance Liquid Chromatography–Mass Spectrometry Approach for Screening of Oxylipins and Their Precursors Arnaud M. Wolfer, Mathieu Gaudin, Simon D. Taylor-Robinson, Elaine Holmes, and Jeremy K. Nicholson Analytical Chemistry 2015 87 (23), 11721-11731 DOI: 10.1021/acs.analchem.5b02794

[6] Dona AC, Jiménez B, Schäfer H, Humpfer E, Spraul M, Lewis MR, Pearce JT, Holmes E, Lindon JC, Nicholson JK. Precision high-throughput proton NMR spectroscopy of human urine, serum, and plasma for large-scale metabolic phenotyping. Anal Chem. 2014 Oct 7;86(19):9887-94. doi: 10.1021/ac5025039. Epub 2014 Sep 16. PMID: 25180432.

[7] Jiménez B, Holmes E, Heude C, Tolson RF, Harvey N, Lodge SL, Chetwynd AJ, Cannet C, Fang F, Pearce JTM, Lewis MR, Viant MR, Lindon JC, Spraul M, Schäfer H, Nicholson JK. Quantitative Lipoprotein Subclass and Low Molecular Weight Metabolite Analysis in Human Serum and Plasma by 1H NMR Spectroscopy in a Multilaboratory Trial. Anal Chem. 2018 Oct 16;90(20):11962-11971. doi: 10.1021/acs.analchem.8b02412. Epub 2018 Sep 27. PMID: 30211542.
