# Supplementary material for: Lipidomics-based plasma signature of alcohol-related hepatitis linked to short-term mortality
Source: JHEP Rep. 2025 Mar 1;7(6):101367. doi: 10.1016/j.jhepr.2025.101367 (PMC12123345; doi:10.1016/j.jhepr.2025.101367)
Supplement: Multimedia component 4 [file mmc4.pdf]

Lipidomics-based plasma signature of alcohol-related hepatitis linked to short-term mortality

Florent Artru[1,2,3,4,†], Stephen Atkinson[2,3,†], Francesca Trovato[1,2], Luke D. Tyson[3], Vishal C. Patel[1,2,5], Nikhil Vergis[3], Noora Kano[3], Robert Goldin[3], Alberto Quaglia[3], Alexandros Pechlivanis[3], Phillip Morgan[1], Salma Mujib[1], Anna Cavazza[1,2], Ellen Jerome[1,2], Marc Zentar[1,2], Roosey Sheth[1,2,3], Maura Morrison[1], Evangelos Triantafyllou[3], Elaine Holmes[3], María Gómez-Romero[6], Mark J. McPhail[1,2,*,‡], Mark Thursz[3,*,‡]

JHEP Reports **2025**. vol. 7 | 1–13

Check for updates

Background & Aims: Severe alcohol-related hepatitis (sAH) is an inflammatory condition with high short-term mortality. Hypothesis-driven approaches have failed to identify effective treatments. Given the role of lipids as inflammatory mediators, this study aimed to identify lipidomic changes and lipid species associated with sAH and mortality risk.

Methods: Untargeted lipidomics was performed on serum samples from two cohorts of patients with sAH and decompensated cirrhosis (DC). Principal component analysis and orthogonal partial least squares discriminant analysis were used to assess lipidome changes. Correlations were made with lipoproteins, lipid mediators, cytokines, cytokeratin fragments, and histological indices.

Results: In the first part, 78 patients with sAH were matched on bilirubin levels with 23 patients with DC. Lipidomics identified a distinct sAH signature involving glycerophospholipids, including PC(34:2) (odds ratio [OR] 2.18, 95% confidence interval [CI] 1.45–7.05, p = 0.01), PC(O–38:5) (OR 3.31, 95% CI 2.23–7.14, p = 0.002), PI(38:4) (OR 0.71, 95% CI 0.46–0.88, p = 0.02), and LPC(18:1) (OR 0.47, 95% CI 0.32–0.82, p = 0.01). These lipids demonstrated excellent discriminatory power between sAH and DC with areas under the receiver operating characteristic curve (AUROCs) between 0.87 and 0.88. In the second part, in 159 sAH patients, specific lipids, including carnitines CAR(2:0) (OR 2.51, 95% CI 1.25–4.96, p = 0.008) and CAR(16:1) (OR 2.21, 95% CI 1.09–7.48, p = 0.009), were linked to 90-day mortality. Acylcarnitines correlated with disease severity parameters such as model for end-stage liver disease, pro-inflammatory cytokines levels, and hepatocyte ballooning on pathology.

Conclusions: Untargeted lipidomics identified a glycerophospholipid and sphingolipid signature distinguishing sAH from DC, implicating lipid species involved in liver regeneration and immune function. Acylcarnitine accumulation in patients with sAH and poor prognosis suggests mitochondrial dysfunction and warrants further investigation into therapeutic potential.

© 2025 The Authors. Published by Elsevier B.V. on behalf of European Association for the Study of the Liver (EASL). This is an open access article under the CC BY license (http://creativecommons.org/licenses/by/4.0/).

Introduction

Severe alcohol-associated hepatitis (sAH) affects young patients with a high short-term mortality: 30% of patients will die within the first 90 days.[1] Liver necroinflammation, defects in hepatic regeneration, gut dysbiosis, systemic inflammation, immune exhaustion, and an increased risk of infection are all contributors to disease pathophysiology and outcome.[2–4]

These mechanisms have been predominantly investigated using hypothesis-driven approaches, yet research efforts have not resulted in novel therapies that improve outcomes.[2] Corticosteroids, the only class of drug currently recommended, are associated with a modest reduction in mortality at 28 days but no therapeutic benefit at 3 months or beyond.[5] To improve therapeutic advances, research in the field of sAH is now applying unbiased approaches.[2,3] The use of large multi-omics datasets has allowed researchers to adopt more agnostic approaches and has identified new pivotal mechanisms and new molecular species to target.[3]

Lipids are organic compounds insoluble in water with a variety of metabolic and nonmetabolic functions. They not only represent an efficient energy substrate but can also act as key inflammatory and anti-inflammatory molecules as part of a network of soluble mediators at the interface of metabolism and the immune system.[6] The role of endogenous bioactive lipid mediators has been demonstrated in several inflammatory diseases (rheumatoid arthritis, inflammatory bowel disease, atherosclerosis, cancer).[6–8] The liver is unique in providing balanced immunotolerance to the exposure of bacterial components from the gut. Specifically, lipids are involved in the natural course of alcohol-related liver disease. Indeed, alcohol increases the hepatic uptake of fatty acids (FAs), primarily released from adipose tissue, resulting in the intrahepatic

* Corresponding authors. Addresses: Institute of Liver Studies, King's College London, Denmark Hill, SE5 9RS, London, UK (M.J. McPhail); Division of Digestive Diseases, Imperial College South Kensington Campus, SW7 2BX, London, UK (M. Thursz).
E-mail addresses: mark.mcphail@kcl.ac.uk (M.J. McPhail), m.thursz@imperial.ac.uk (M. Thursz).
† Joint first authors.
‡ Joint last authors.
https://doi.org/10.1016/j.jhepr.2025.101367

accumulation of triglycerides (TGs).[9–11] Furthermore, alcohol alters FA oxidation and lipid transport, further increasing lipogenesis and steatosis. A recent comprehensive study has revealed that the progression of alcohol-related liver fibrosis is closely linked to significant alterations in the plasma and liver lipidome. These changes predominantly affect sphingolipid (SL) classes, with a marked disruption of their metabolic pathways and a progressive depletion of their components. A depletion in most phosphocholines (PCs) was also observed.[12] In the setting of decompensated cirrhosis (DC) and acute-on-chronic liver failure (ACLF), untargeted and targeted lipidomics identified a plasma fingerprint of these diseases including eicosanoids, SL, and lysophosphatidylcholines (LPC) that are associated with their dynamic evolution.[13,14] In patients with ACLF, the dysregulation of the LPC–autotaxin (ATX)–lysophosphatidic acid (LPA) axis was associated with mortality and inflammation through an LPA-dependent monocyte activation.[15] Among endogenous bioactive lipids, the role of fatty acyls, including eicosanoids and acylcarnitines, pro-resolving lipid mediators, lysophospholipids, and SL, has been investigated in the setting of sAH. Although recent work has indicated an association between acylcarnitine metabolism and prognosis in sAH, comparisons have not been made with important disease controls such as DC.[16] Untargeted lipidomics was performed in two large prospectively recruited cohorts of patients with sAH and DC (from the Steroid Or Pentoxifylline for Alcoholic Hepatitis [STOPAH] trial[17] and the Gut–Liver Axis study) to evaluate whether lipidomics could identify key lipid mediators and their class involved in the pathogenesis of sAH and its complications.

Materials and methods

Patients

Patients with sAH were recruited via the STOPAH trial as per the trial protocol.[17] Briefly, patients were randomised to treatment with prednisolone, pentoxifylline, both, or neither for 28 days using a double-blind, double-dummy, factorial 2 × 2 design. Outcome data were collected for mortality at 28 and 90 days. The Wales Research Ethics Committee (REC 09/MRE09/59) granted ethical approval for this study. To optimise the discovery of metabolites and pathways associated with 90-day mortality and independent of liver disease severity, patients were subselected from the overall cohort based upon (1) the presence of steatohepatitis on biopsy and/or (2) the absence of end-stage liver disease illustrated by model for end-stage liver disease (MELD) score >30. The final cohort included 166 patients with similar liver function assessed using the MELD score. In the sAH cohort, serum samples for all analyses performed in the present study were collected the day of treatment initiation.

Patients with alcohol-related cirrhosis recruited to the prospective longitudinal Compensated Cirrhosis Cohort in Nottingham study (Ref 10/H0403/10; approved by East Midlands Nottingham 1 ethics committee) as well as patients recruited to the Gut–Liver Axis study (London – Westminster Research Ethics Committee No. 12/LO/1417; IRAS No 104301) were used as an additional control group. The study was conducted according to the Declaration of Helsinki (Hong Kong Amendment) and Good Clinical Practice (European guidelines). All participants, or their legally appointed representatives, provided written informed consent. For the purpose of the study, patients with sAH were matched to patients with DC without sAH based on serum bilirubin ±30 μmol/L.

Lipidomics

Serum lipidomic analyses were performed by ultrahigh performance liquid chromatography coupled to mass spectrometry (UHPLC-MS) after isopropanol protein precipitation (see Supplementary material). MassLynx software 4.1 (Waters, Milford, MA, USA) was used for data acquisition. For the processing of the data, UHPLC-MS raw data files in positive and negative ionisation modes were converted to NetCDF format (using databridge, within MassLynx) and extracted via XCMS (version 1.24.1) package within R (version 2.11; R Foundation for Statistical Computing, Vienna, Austria) software (see Supplementary material). XCMS analysis of these data provided a matrix containing the retention time, *m/z* value, and integrated peak area for aligned features across samples. Every feature of interest identified through supervised multivariable statistical analysis (see below) and volcano plots was subsequently annotated by matching the accurate mass of the molecular ion to reference spectra contained in publicly available databases.

Lipid mediator-targeted lipidomics

Serum lipid mediators were extracted using solid-phase extraction (SPE) in a mixed-mode plate with a strong anion exchanger (Oasis MAXμElution 96-well plate; Waters). Concentrations were quantified using UHPLC coupled to tandem mass spectrometric detection (MS/MS) using a targeted method[18] validated to detect up to 48 lipid mediators, of which 26 were quantifiable within this study (see Supplementary material for further details on sample extraction and analysis).

Liproprotein

Lipoprotein parameters were measured using the B.I. LISA (Bruker Biospin, Billerica, Mass, USA) analysis pipeline for ^{1}H-NMR spectroscopy, according to the manufacturer's instruction.[19] The spectra generated by standardised sequences can be analysed to quantify a lipoprotein panel of 114 parameters including the main VLDL, intermediate-density lipoprotein (IDL), LDL, and HDL and their subclasses (see Supplementary materials).

Cytokines, chemokines, and immune and renal markers analyses

Plasma cytokines were measured using Meso Scale Discovery (Meso Scale Diagnostics LLC, Rockville, MD, USA) proinflammatory multiplex assay kits, as described in the Supplementary materials. The panel kits included platelet-derived growth factor subunit A (PDGFA); transforming growth factor betas 1, 2, and 3 (TGFβ1, TGFβ2, and TGFβ3); cluster of differentiation 163 (CD163); cystatin C; epidermal growth factor (EGF); hepatocyte growth factor (HGF); interferon (IFN); insulin-like growth factor (IGF); interleukins IL-10, IL-18, IL-1 receptor antagonist (IL-1RA), IL-1α, IL-1β, IL-22, IL-23, IL-6, and IL-8; lipopolysaccharide-binding protein (LBP); neutrophil gelatinase-associated lipocalin (NGAL); programmed death-1 (PD-1); programmed death-ligand 1 (PD-L1); tumour necrosis factor alpha (TNFα); TNF-like weak inducer of apoptosis (TWEAK); and vascular endothelial growth factor (VEGF).

Serum keratin-18 fragments

The M65 antibody-detected protein reflects total cell death, whereas the M30 antibody-detected fragment is generated when K18 is cleaved during apoptosis. M65/M30 ratio reflects the contribution of apoptosis to cell death pathways.[20] Quantification of total K18–M65 and caspase-cleaved K18–M30 was performed using ELISA (VLVbio, Stockholm, Sweden).[21]

Histological analyses

Two experienced histopathologists (RG and AQ), blinded to patient treatment and outcomes, independently assessed the histological features of each biopsy using the alcoholic hepatitis (AH) histological scoring system (AHHSS).[22] The presence or absence of Mallory–Denk bodies and megamitochondria was also recorded.

Statistical analyses

Continuous variables were expressed in median and IQR, and categorical variables were expressed in number and percentages. Comparisons between groups were performed using the Mann-Whitney *U* test for quantitative variables or the Chi-square test and Fisher's exact test for categorical variables, as appropriate. The significance level was set at 0.05 for a two-sided test. Principal components analysis (PCA), orthogonal partial least squares discriminant analysis (OPLS-DA), and the development of scores and volcano plots analyses are detailed in the Supplementary materials. All the analyses were performed through SIMCA version 16.0 (Sartorius Stedim, Aubagne, France), NCSS version 2022 (NCSS, LLC, Kaysville, Utah, USA), and GraphPad Prism version 9.0 (GraphPad Software, San Diego, CA, USA).

Results

Exploratory analyses of patients with sAH

Untargeted lipidomics was performed in 166 patients with a clinical diagnosis of sAH according to the STOPAH inclusion criteria.[17] Among them, 76 were confirmed by liver biopsy, seven patients underwent liver biopsy without any features of the condition after pathology examination, and 83 patients did not undergo liver biopsy. The seven patients with the absence of sAH features on biopsy were excluded from the final analyses. PCA did not demonstrate a visual difference, and OPLS-DA models comparing patients with sAH and a positive biopsy and patients with sAH who were not biopsied (Fig. S1) suggested that from the lipidome side, these patients were not different (comparison of clinical and biological data is provided in Table S1). Consequently, these patients were merged in the same group for final analyses with the final group of sAH (n = 159).

Patients with sAH have a distinct lipidomic profile compared with patients with DC

In the first part, we aimed to explore the lipidomic signature of patients with sAH compared with matched patients with DC without sAH. To achieve this, we matched up to four patients with sAH to one patient with DC based on serum bilirubin ±30 μmol/L. Hence, 78 of the 159 patients with sAH and 23 of the 74 patients with cirrhosis who underwent untargeted lipidomics were included in these analyses. Comparison between the groups of patients with sAH and cirrhosis after matching on bilirubin level is provided in Table 1. Comparison between the overall sAH cohort (n = 159) and overall patients with cirrhosis (n = 74) is provided in Table S2.

In positive ionisation mode, OPLS-DA gave robust differentiation in lipidome between patients with sAH and patients with DC (three-component model 1 + 2 + 0, $R^2 = 0.64$, $Q^2 = 0.47$, CV-ANOVA $p < 0.0001$) (Fig. 1A). A permutations plot confirmed the validity of the model. The area under the receiver operating characteristic curve (AUROC) of the positive ionisation mode model was 0.90 (95% confidence interval [CI] 0.85–0.94, $p < 0.0001$) to differentiate between the two conditions (Fig. 1B and C). According to variable projection of importance (VIP) plots, 74 features corresponding to 29 identifiable lipids had a value ≥2 and are illustrated in Fig. 1D and E and listed in Table S3. These lipids mainly belonged to the glycerophospholipid and sphingomyelin subclasses. We inputted these 29 lipids in univariable and multivariable logistic regression analyses. Two lipids, phosphatidylcholine PC(34:2) and PC-O(38:5), independently differentiated between patients with sAH and those with DC even after adjustment for the MELD score (PC(34:2): OR 2.18, 95% CI 1.45–7.05, $p = 0.01$; PC-O(38:5): OR 3.31, 95% CI 2.23–7.4, $p = 0.002$) (Table 2). The model based on these two lipids outperformed the MELD score alone in differentiating sAH from DC with an AUROC of 0.87 (0.77–0.93) *vs.* 0.70 (0.54–0.82) respectively ($p = 0.02$). Adjusting for the MELD score further improved its performance with an AUROC of 0.93 (0.84–0.97, $p = 0.05$) (Fig. 1F).

In negative ionisation mode, OPLS-DA provided a similarly valid model that was able to differentiate patients with sAH from those with DC (three-component model 1 + 2 + 0, $R^2 = 0.61$, $Q^2 = 0.35$, CV-ANOVA $p < 0.0001$) with an AUROC of 0.93 (95% CI 0.87–0.97, $p < 0.0001$) (Fig. 2A–C). Forty features corresponding to 11 identifiable lipids had a value ≥2 in the VIP plot (Fig. 2D and E and Table S4). These were mainly glycerophospholipids and glycophosphoinositols. In univariable and multivariable logistic regression analyses, two lipids were able to independently differentiate between the two conditions even after adjustment for the MELD score: phosphatidylinositol PI(38:4) (OR 0.71, 95%CI 0.46–0.88, $p = 0.02$) and LPC(18:1) (OR 0.47, 95% CI 0.32–0.82, $p = 0.01$) (Fig. 2G and Table 3). The model based on these two lipids outperformed the MELD score alone in differentiating sAH from DC, with an AUROC of 0.88 (0.78–0.94) *vs.* 0.70 (0.54–0.82), respectively ($p = 0.04$). The two-lipid model was not improved after adjustment for the MELD score (0.91, 0.83–0.96, $p = 0.07$) (Figure 2F).

Patients with sAH have a distinct lipoprotein and cytokine signature as compared with patients with DC

Lipoproteome of patients with sAH differed from that of patients with DC. Indeed, OPLS-DA of lipoproteomics provided valid models differentiating sAH from DC (Fig. S2A–D). The six lipoprotein parameters with the greatest discriminant abilities were LDL particle number (LDPN), total blood particle number (TBPN), lipoprotein subclass 4 particle number (L4PN),

lipoprotein subclass 3 particle number (L3PN), intermediate-density lipoprotein particle number (IDPN), and lipoprotein subclass 1 particle number (L1PN), which were all elevated in sAH compared with DC (Fig. S2G). In addition, among a panel of 27 cytokines, chemokines, and immune and renal markers, eight had different circulating levels between the two conditions: CD163, cystatin C, IL-18, PD-L1, and PD-1 concentrations were decreased in patients with sAH compared with patients with DC; conversely, IL-8, TNFα, and VEGF levels were increased (Fig. S2H). To gain insights into aspects of the clinical phenotype associated with discriminatory lipid species, these lipids were combined with clinical, laboratory, and serum cytokine data, and a bi-clustered cross-correlation matrix was generated and is shown in Fig. S3A (positive ionisation mode lipids) and B (negative ionisation mode lipids). In positive ionisation, we observed co-clustering with positive correlation of the lipoproteins together with several glycerophospholipids and sphingolipids and negative correlation with LPC(16:0) and sphingomyelin SM (d18:1/16:0). LPC(16:0) was strongly negatively correlated with the MELD score (-0.446, p <0.0001), bilirubin level (-0.478, p <0.0001), IDPN (-0.467, p <0.0001), L1PN (-0.347, p = 0.002), L3PN (-0.443, p <0.0001), L4PN (-0.470, p <0.0001), and inflammation markers (white blood cell [WBC] count, neutrophil count, and IL-8) (Fig. S4A). In negative ionisation, we also observed co-clustering of the lipoproteins together with several glycerophospholipids. Among them, PC(36:5) was positively correlated with platelet count (0.42, p <0.0001), TBPN (0.502, p <0.0001), IDPN (0.401, p <0.0001), LDPN (0.508, p <0.0001), L3PN (0.422, p <0.0001), and L4PN (0.440, p <0.0001) and was negatively correlated with CD163 (-0.470, p <0.0001), PD-L1 levels (-0.469, p <0.0001), and international normalised ratio (INR) (-0.402, p <0.0002) (Fig. S4B).

Exploration of correlation between lipid mediators and key lipids from an untargeted approach

Forty seven of the 78 patients with sAH (60%) and 15 of the 23 patients with DC (65%) underwent plasma targeted lipid mediators lipidomics. Based on logFCs and p values, volcano plots isolated five lipid mediators differentiated between conditions, and all were increased in patients with sAH compared with patients with DC: prostaglandin E2 (PGE2), prostaglandin F2α (PGF2α), 11-hydroxyeicosatetraenoic acid (11-HETE), 9-hydroxyoctadecadienoic acid (9-HODE), and 15-hydroxyeicosatetraenoic acid (15-HETE) (Fig. 3A and B). In positive mode, PC(O-38:5), one of the two lipids included in the positive model, positively correlated with PGF2α (0.403, p = 0.0004) 11-HETE (0.405, p = 0.0005), and 15-HETE (0.411, p = 0.0002) (Fig. 3C). In negative mode, PI(38:4), one of the two lipids included in the negative model, positively correlated with 11-HETE (0.421, p = 0.0003) and 15-HETE (0.402, p = 0.001) (Fig. 3D).

Acylcarnitines and phosphatidylcholines are associated with outcome of sAH

In the cohort of patients with sAH (n = 159), OPLS-DA models in both positive and negative ionisation modes were not robust and/or valid to identify survivors (n = 106) *vs.* non-survivors (n = 51) in the sAH cohort (Fig. S5A–H). Volcano

Table 1. Characteristics of patients at the time of sampling after matching based on bilirubin level (overall cohort, N = 101; patients with alcohol-related hepatitis, n = 78; patients with cirrhosis, n = 23).

	Population included in the matched analyses (N = 101)	Patients with alcohol-related hepatitis included in the matched analyses (n = 78)	Patients with cirrhosis included in the matched analyses (n = 23)	*p* value
**Characteristics**				
Age (years)	52.0 (45–60.0)	52.0 (44.0–60.0)	52.0 (45.0–61.0)	0.97
Sex (male)	64 (63.4)	46 (61.5)	16 (69.5)	0.48
BMI (kg/m^2)	24.5 (22.5–29.8)	26.1 (22.5–30.1)	22.9 (22.4–23.8)	0.03
Alcohol-related liver disease	98 (97.0)	78 (100)	20 (86.9)	0.32
Biopsy (yes)	–	38 (48.7)	–	–
**Laboratory**				
Leucocytes (G/L)	8.6 (6.0–11.5)	8.9 (6.2–11.7)	8.0 (5.3–10.9)	0.85
Neutrophils (G/L)	6.0 (3.9–8.9)	6.0 (4.2–8.9)	6.3 (3.6–8.9)	0.42
Haemoglobin (g/L)	104.0 (91.0–116.0)	105.0 (91.0–112.0)	102.0 (89.0–108.0)	0.18
Platelets (G/L)	96.0 (82.0–153.5)	110.0 (75.0–175.0)	66.0 (48.0–116.0)	0.003
INR	1.7 (1.5–2.1)	1.7 (1.5–2.0)	2.0 (1.6–2.8)	0.06
Bilirubin (µmol/L)	253.0 (153.5–359.0)	252.0 (176.0–360.0)	225 (159.0–372.0)	0.29
AST (IU)	120.0 (78.5–155.5)	132.5 (99.5–171.5)	64.0 (49.0–140.0)	0.87
ALP (IU)	170.0 (120.3–254.5)	183.0 (133.0–253.0)	119.0 (82.0–166.0)	0.02
Albumin (g/L)	24.0 (20.5–29.0)	23.0 (20.0–28.0)	26.0 (22.0–33.0)	0.06
Creatinine (µmol/L)	69.0 (55.5–94.0)	64.5 (52.0–80.3)	106 (62.0–167.0)	<0.0001
Urea (mmol/L)	3.4 (2.2–5.7)	3.0 (2.0–4.9)	6.6 (3.3–16.6)	0.0002
Sodium (mmol/L)	134.0 (131.0–137.0)	134.0 (130.0–137.0)	136.0 (133.0–138.0)	0.12
**Scores**				
MELD	22.7 (20.6–26.9)	22.1 (20.5–25.7)	27.6 (22.4–37.6)	0.002
Maddrey's discriminant function	48.9 (41.1–71.2)	48.9 (41.1–71.2)	–	–
Lille	0.6 (0.3–0.8)	0.6 (0.3–0.8)	–	–
**Outcome**				
Death at 3 months	32 (31.7)	26 (33.3)	6 (26.1)	0.51

Continuous and categorical variables expressed respectively in median (IQR) and n (percentages), respectively. The Mann-Whitney *U* test was used for quantitative variables, and the Chi-square test and Fisher's exact test were used for categorical variables, as appropriate. ALP, alkaline phosphatase; AST, aspartate aminotransferase; MELD, model for end-stage liver disease.

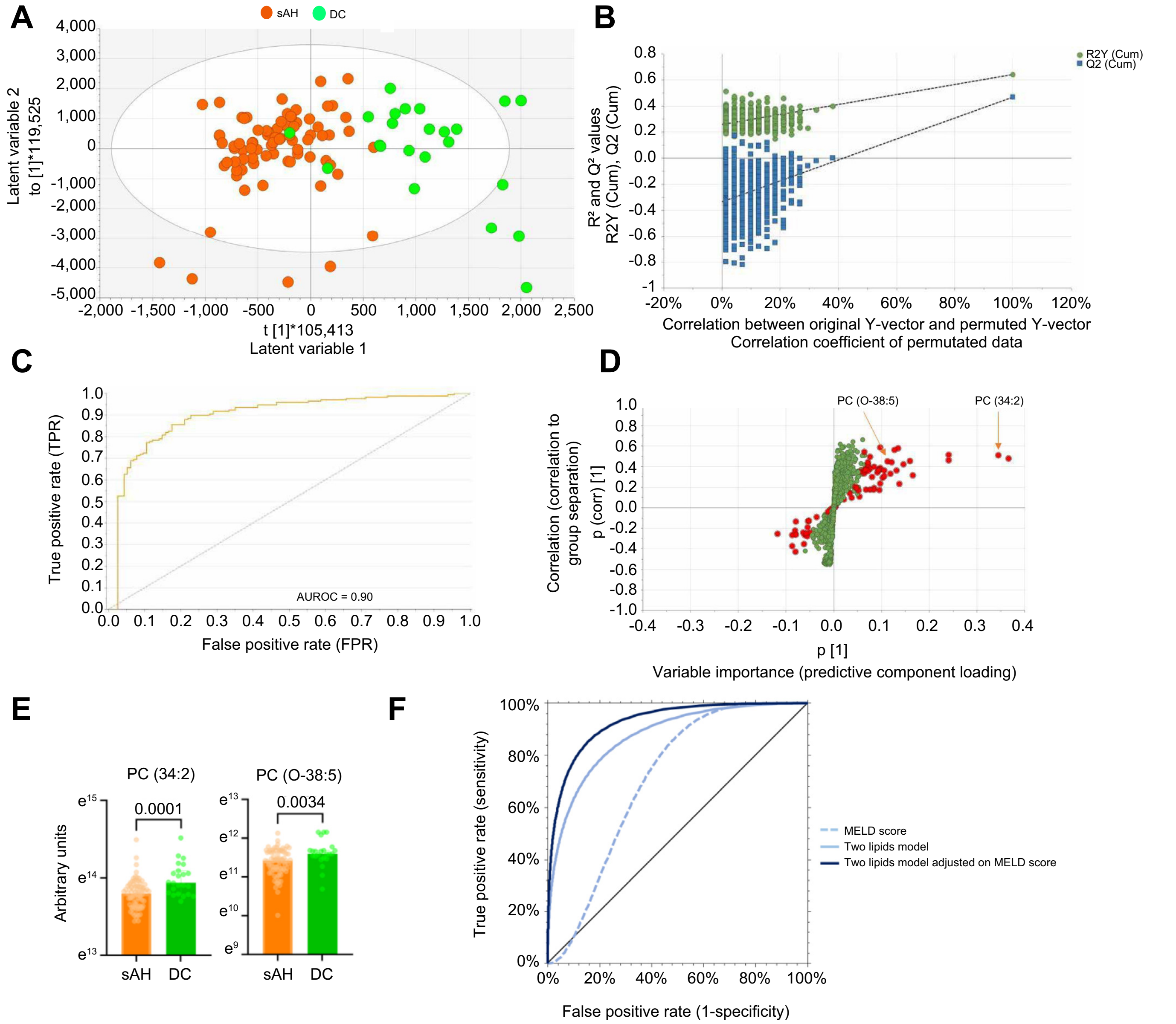

Model 1	Model 2	p value
Two lipids model: 0.87 (0.77-0.93)	MELD score: 0.70 (0.54-0.82)	0.02
Two lipids model: 0.87 (0.77-0.93)	Two lipids model adjusted on MELD score: 0.93 (0.84-0.97)	0.05
Two lipids model adjusted on MELD score: 0.93 (0.84-0.97)	MELD score: 0.70 (0.54-0.82)	<0.0001

Fig. 1. Untargeted lipidomics in positive ionisation mode in patients with sAH (n = 78) matched to patients with DC (n = 23) on bilirubin level. (A) Score plot of the model. Each dot represents the model in one patient: sAH in orange and DC in green. It shows how the samples are grouped based on their lipidome. Two distinct clusters suggest that the two groups are separable from a lipidome perspective. (B) Permutation test demonstrating the validity of the model. A permutation plot tests whether the OPLS-DA model's ability to separate groups is real or as a result of random chance. A good model shows much higher performance metrics (R^2 and Q^2) for the actual data compared with randomised models, with a clear downward trend in performance as the data are permuted. (C) AUROC using the discriminant variables of the OPLS-DA model. (D) S-plot of the model. Each variable is plotted. Variables in red are those with a VIP value ≥2. This plot shows the relationship between the variables' importance (x-axis) and their correlation with group separation (y-axis). Points far from the centre are the most relevant variables driving the separation. They may correspond to key biomarkers. (E) Univariable analyses of the two lipids identified from multivariable logistic regression as independently differentiating between patients with sAH and DC (PC(34:2), p = 0.0001, and PC-O(38:5), p = 0.0034, Mann-Whitney U test). (F) Performance based on the AUROC of the two-lipid model, two-lipid models adjusted for the MELD score, and the MELD score alone in differentiating patients with sAH from patients with DC. Two-lipid model *vs*. MELD score: p = 0.02; two-lipid model *vs*. two-lipid model adjusted for the MELD score: p = 0.05; two-lipid model adjusted for the MELD score *vs*. MELD score: p <0.0001 (Z test). AUROC, area under the receiver operating characteristic curve; DC, decompensated cirrhosis; MELD, model for end-stage liver disease; OPLS-DA, orthogonal partial least squares discriminant analysis; sAH, severe alcohol-related hepatitis; VIP, variable projection of importance.

Table 2. Univariable and multivariable logistic regression analyses of lipid species associated with decompensated cirrhosis condition compared with severe alcohol-related hepatitis in positive ionisation mode.

	Univariable analysis			Multivariable analysis		
Covariant	OR	95% CI	*p* value	OR	95% CI	*p* value
PC(34:1)	2.32	1.26–4.28	0.007	1.83	0.50–6.70	0.36
PC(34:2)	2.73	1.54–4.83	0.0006	2.18<br>2.59*	1.45–7.05<br>1.21–7.51*	0.01<br>0.02*
SM(d18:1/16:0)	1.71	1.11–2.65	0.02			
TG(52:2)	1.37	0.88–2.15	0.15			
TG(52:3)	1.45	0.89–2.36	0.13			
PC(36:3)	1.99	1.13–3.50	0.02			
TG(54:3)	0.93	0.58–1.50	0.77			
TG(54:4)	1.43	0.89–2.30	0.14			
TG(54:5)	1.56	0.94–2.56	0.08			
PC(36:1)	0.68	0.43–1.09	0.12			
TG(52:4)	1.27	0.79–2.06	0.32			
PC(36:2)	1.03	0.64–1.05	0.90			
TG(50:2)	0.78	0.92–1.48	0.74			
PC(38:3)	0.57	0.35–0.94	0.03			
TG(50:1)	2.13	1.28–3.56	0.003	1.57	0.64–3.84	0.28
PC(O-38:5)	2.46	1.32–4.57	0.004	3.31<br>2.82*	2.23–7.14<br>1.22–6.54*	0.002<br>0.01*
SM(d18:2/24:0)	0.72	0.47–1.13	0.16			
TG(54:2)	0.68	0.42–1.08	0.11			
TG(50:3)	0.57	0.35–0.91	0.02			
PC(36:5)	1.35	0.82–2.19	0.22			
PC(36:4)	1.54	0.93–2.54	0.09			
TG(48:2)	0.54	0.33–0.87	0.01	0.39	0.15–1.02	0.07
PC(38:4)	0.73	0.45–1.17	0.20			
LPC(16:0)	1.20	0.74–1.97	0.45			
PC(38:5-OH)	2.02	1.14–3.60	0.02			
TG(50:4)	0.53	0.32–0.86	0.01	1.56	0.53–4.57	0.42
TG(56:6)	0.68	0.44–1.06	0.09			
TG(56:8)	0.52	0.32–0.84	0.008	0.67	0.28–1.55	0.45
PC(36:5)	1.70	1.03–2.82	0.04			

Effect size calculated per standard deviation increase. Independent association observed for PC(34:2): p = 0.01 and p = 0.02 after adjustment for the MELD score; PC(O-38-5): p = 0.002 and p = 0.01 after adjustment for the MELD score. *After adjustment for the MELD score (1.21, 1.08–1.35, p = 0.003). CI, confidence interval; MELD, model for end-stage liver disease; OR, odds ratio.

plots isolated eight lipids in positive ionisation mode (Fig. 4A and Table S5) and five lipids in negative ionisation mode (Fig. 4D and Table S6), differentiating survivors from non-survivors at day 90. Univariable and multivariable logistic regression identified two lipids in positive ionisation mode (carnitine CAR(2:0): OR 2.51, 95% CI 1.25–4.96, p = 0.008 and CAR(16:1): OR 2.21, 95% CI 1.09–7.48, p = 0.009) and two lipids in negative ionisation mode (PC(36:4): OR 0.38, 95%CI 0.15–0.89, p = 0.03 and fatty acyl FA(16:0): OR 1.53, 95% CI 1.08–2.19, p = 0.02) as independently associated with 90-day mortality status (Fig. 4B and E, Table 4, and Table S7). The resulting model in positive ionisation mode performed better than the Lille model and the MELD score in discriminating between survivors vs. non-survivors at 90 days (Fig. 4C). In negative ionisation mode, the two-lipid model failed to improve the prognostic ability of the Lille model even when combined with it (Fig. 4F). As in the first part, we next explored whether lipoproteome differed between survivors *vs*. non-survivors. The OPLS-DA model was not valid for discriminating between the two conditions (Fig. S6A–D). Volcano plot and univariable analysis identified VLDL subclass 2 cholesterol (V2CH) level as decreased in non-survivors (Fig. S6E and F). Among the 27 cytokines panel, only three differed between survivors and non-survivors and were increased in the latter group: IGF, IL-22, and IL-6 (Fig. S6G). None of the lipid mediators reached the predefined threshold on volcano plots (Fig. S6H). CK-18 M65 and M30 fragments as well as their ratio were evaluated in the cohort and are provided in Fig. S6I.

The correlation matrix of clinical and routine laboratory data, lipids identified in volcano plots, lipoproteins, and cytokines differentiating the D90 status of sAH as well as CK-18 M65, M30 fragments, and their ratio are provided in Fig. S7A (positive ionisation lipids) and B (negative ionisation lipids).

In positive ionisation mode, CAR(2:0), CAR(16:0), and CAR(16:1) negatively co-clustered with phosphatidylcholines PC(38:7) and PC(36:4). Notably, CAR(2:0) was positively correlated with the MELD score (0.363, p <0.0001), Glasgow AH score (GAHS) (0.384, p <0.0001), creatinine (0.401, p <0.0001), IL-6 (0.362, p = 0.0006), IGF (0.342, p = 0.007), and IL-22 (0.348, p = 0.0001) and negatively correlated with PC(36:4) (-0.475, p <0.0001) and PC(38:7) (-0.553, p <0.0001) (Fig. S8A). In negative ionisation mode, although PC(36:4) and FA(16:0) showed no strong correlation with any other laboratory parameters, scores, cytokines, and lipoproteins included in the correlation matrix, LPC(18:0) showed a strong negative correlation with IL-6 (-0.341, p = 0.0001) and biological routine severity data such as the MELD score (-0.461, p <0.0001), GAHS score (-0.330, p <0.0001), or Lille model (-0.381, p = 0.0002) and a positive correlation with PC(36:4)

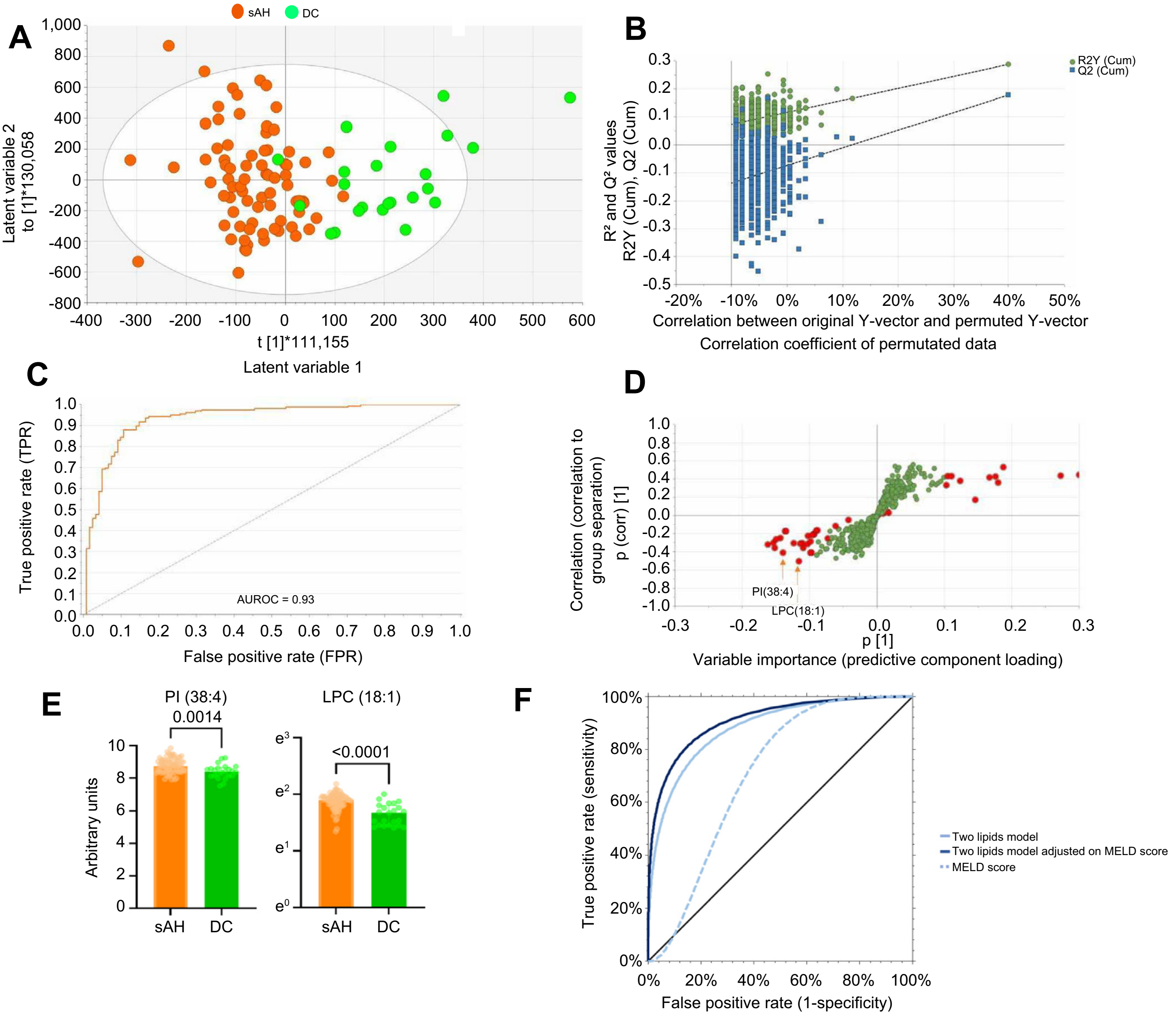

Model 1	Model 2	*p* value
Two lipids model: 0.88 (0.78-0.94)	MELD score: 0.70 (0.54-0.82)	0.04
Two lipids model: 0.88 (0.78-0.94)	Two lipids model adjusted on MELD score: 0.91 (0.83-0.96)	0.07
Two lipids model adjusted on MELD score: 0.91 (0.83-0.96)	MELD score: 0.70 (0.54-0.82)	0.01

Fig. 2. Untargeted lipidomics in negative ionisation mode in patients with sAH (n = 78) matched to patients with DC (n = 23) on bilirubin level. (A) Score plots of the model. Each dot represents the model in one patient: sAH in orange and DC in green. It shows how the samples are grouped based on their lipidome. Two distinct clusters suggest that the two groups are separable from a lipidome perspective. (B) Permutation test demonstrating the validity of the positive ionisation mode. A permutation plot tests whether the OPLS-DA model's ability to separate groups is real or as a result of random chance. A good model shows much higher performance metrics (R^2 and Q^2) for the actual data compared with randomised models, with a clear downward trend in performance as the data are permuted. (C) AUROC using the discriminant variables of the OPLS-DA model. (D) S-plot of the model. Each variable is plotted. Variables in red are those with a VIP value ≥2. This plot shows the relationship between the variables' importance (x-axis) and their correlation with the group separation (y-axis). Points far from the centre are the most relevant variables driving the separation. They may correspond to key biomarkers. (E) Univariable analyses of the two lipids identified from multivariable logistic regression as independently differentiating between patients with sAH and DC (PI(38:4), p = 0.0014, and LPC(18:1), p <0.0001, Mann-Whitney *U* test). (F) Performance based on the AUROC of the two-lipid model, two-lipid models adjusted for the MELD score, and MELD score alone in differentiating between patients with sAH and patients with DC. Two-lipid model *vs*. MELD score: p = 0.04; two-lipid model *vs*. two-lipid model adjusted for the MELD score: p = 0.07; two-lipid model adjusted for the MELD score *vs*. MELD score: p = 0.01 (Z test). AUROC, area under the receiver operating characteristic curve; DC, decompensated cirrhosis; MELD, model for end-stage liver disease; OPLS-DA, orthogonal partial least squares discriminant analysis; sAH, severe alcohol-related hepatitis; VIP, variable projection of importance.

Table 3. Univariable and multivariable logistic regression analyses of lipid species associated with decompensated cirrhosis condition compared with severe alcohol-related hepatitis in negative ionisation mode.

	Univariable analysis			Multivariable analysis		
Covariant	OR	95% CI	*p* value	OR	95% CI	*p* value
PC(34:1)	0.53	0.31–0.91	0.02			
PI(38:4)	0.40	0.22–0.71	0.002	0.71<br>0.75*	0.46–0.88<br>0.53–0.98*	0.02<br>0.05*
PC(38:3)	0.53	0.31–0.91	0.02			
PC(38:4)	0.57	0.33–0.98	0.04			
PC(36:1)	0.57	0.32–0.99	0.05			
PC(36:2)	0.80	0.51–1.27	0.35			
PC(36:5)	0.54	0.33–0.91	0.02			
PC(34:2)	1.39	0.86–2.25	0.18			
PC(32:1)	0.72	0.44–1.17	0.18			
LPC(18:1)	0.49	0.32–0.90	0.01	0.47<br>0.42*	0.32–0.82<br>0.27–0.88*	0.01<br>0.03*
LPC(18:2)	0.71	0.46–1.07	0.09			

Effect size calculated per standard deviation increase. Independent association observed for PI(38:4): $p = 0.02$ and $p = 0.05$ after adjustment for the MELD score; LPC(18:1): $p = 0.01$ and $p = 0.03$ after adjustment for the MELD score. *After adjustment for the MELD score (1.09, 0.89–1.32, $p = 0.44$). CI, confidence interval; MELD, model for end-stage liver disease; OR, odds ratio.

(0.460, p <0.0001), which was included in the two-lipid model associated with the D90 status (Fig. S8B).

Association between lipids and baseline infection, response to treatment according to the Lille model, and incident events known to be associated with the prognosis of sAH, such as incident acute kidney injury (AKI) and infection (see definition in Supplementary materials), were further explored. For the analyses of incident AKI, patients with AKI at D0 (n = 82) or those without creatinine level data between D0 and D7 (n = 14) were excluded. Volcano plots identified seven lipids in positive ionisation mode (among which CAR(16:1) and PC(38:7) were already identified as associated with the D90 status) associated with incident AKI status. No lipids in negative ionisation mode were associated with incident AKI status (Fig. S9A and B). Incident infection was defined as infection occurring from the day of treatment initiation to last follow-up.[17] For these analyses, patients who received antimicrobial treatment within the last 48 h before sampling (n = 38) were excluded. Volcano plots identified 14 lipids in positive ionisation mode (among which PC(36:4) and PC(38:7) were already identified as associated with the D90 status) and four in negative ionisation mode (among which PC(36:4) and PC(37:6) were already associated with the D90 status) associated with incident infection (Fig. S9C and D). Notably, CAR(16:1) and CAR(18:1) were among the 11 lipids identified in the volcano plot as differentiating responders from non-responders according to the Lille model (Fig. S9E–H).

Pathology

Finally, we aimed to evaluate the relationship between pathology features and key lipids differentiating survivors from non-survivors at D90. We performed a correlation analysis between these lipids, CK-18 M65, M30 fragments, and their ratio and histological features such as AHHSS score, neutrophil infiltration, ballooning, Mallory–Denk bodies, bilirubinostasis, megamitochondria presence, and fibrosis according to Laennec scoring system. Inflammation and steatosis, as defined by AHHSS, were not included as constant in the cohort with positive liver biopsy feature. The correlation matrix with key lipids from positive and negative ionisation modes is provided in Fig. S10A and B, respectively. In positive ionisation mode, ballooning was the only pathology variable associated with lipid signature associated with the D90 status (negatively correlated with PC(36:4) and PC(38:7) and positively with CAR(16:1)). In negative ionisation, the presence of Mallory–Denk bodies was negatively correlated with all lipid signatures except FA(16:0).

Discussion

In this largest lipidomic analysis of patients with sAH, we identified how lipid biology can distinguish the syndrome from DC and assist in mortality prediction.

A small number of lipid species can be used to differentiate sAH from DC predominantly based on glycerophosphocholines. Concentrations of these lipids correlated with systemic inflammation markers, LDL, and pro-inflammatory lipid mediators. The second part suggests that, in the sAH population, the main lipids associated with clinical events (death, incident AKI, and infection), and some histological features of the disease are acylcarnitines and glycerophosphocholines. Considering the increasing amount of evidence showing that these species can modulate key features of sAH (inflammation, immune dysfunction, and liver regeneration and repair), this study implicates them in the aetiopathogenesis of sAH and provides novel avenues to explore therapeutic targets.

It is important to note that in our study, we selected patients by matching patients with sAH to those with DC based on their serum bilirubin. This was to account for the effect of liver dysfunction and cholestasis on the circulating lipidome. In the second part, the analyses were restricted to patients with sAH from the initial STOPAH cohort to identify metabolites that may predict outcome independently of liver disease severity. As such, the cohort comprised two groups of patients who differed in outcome but who had similar age and MELD scores.

PC/LPC lipid subclasses are key discriminating metabolites between sAH and DC as well as between sAH with favourable and poor outcome (especially regarding the risk of 90-day mortality and incident infection). These lipids regulate liver repair and lipolysis and exhibit immune-modulatory functions (enhancing chemotaxis, stimulating phagocytosis, and upregulating the expression of adhesion molecules) reducing the organ injury and dysfunction in septic shock.[23–25] LPC injections induced lipoapoptosis of hepatocytes and lobular hepatitis in mice.[26,27] In studies evaluating the impact of acute

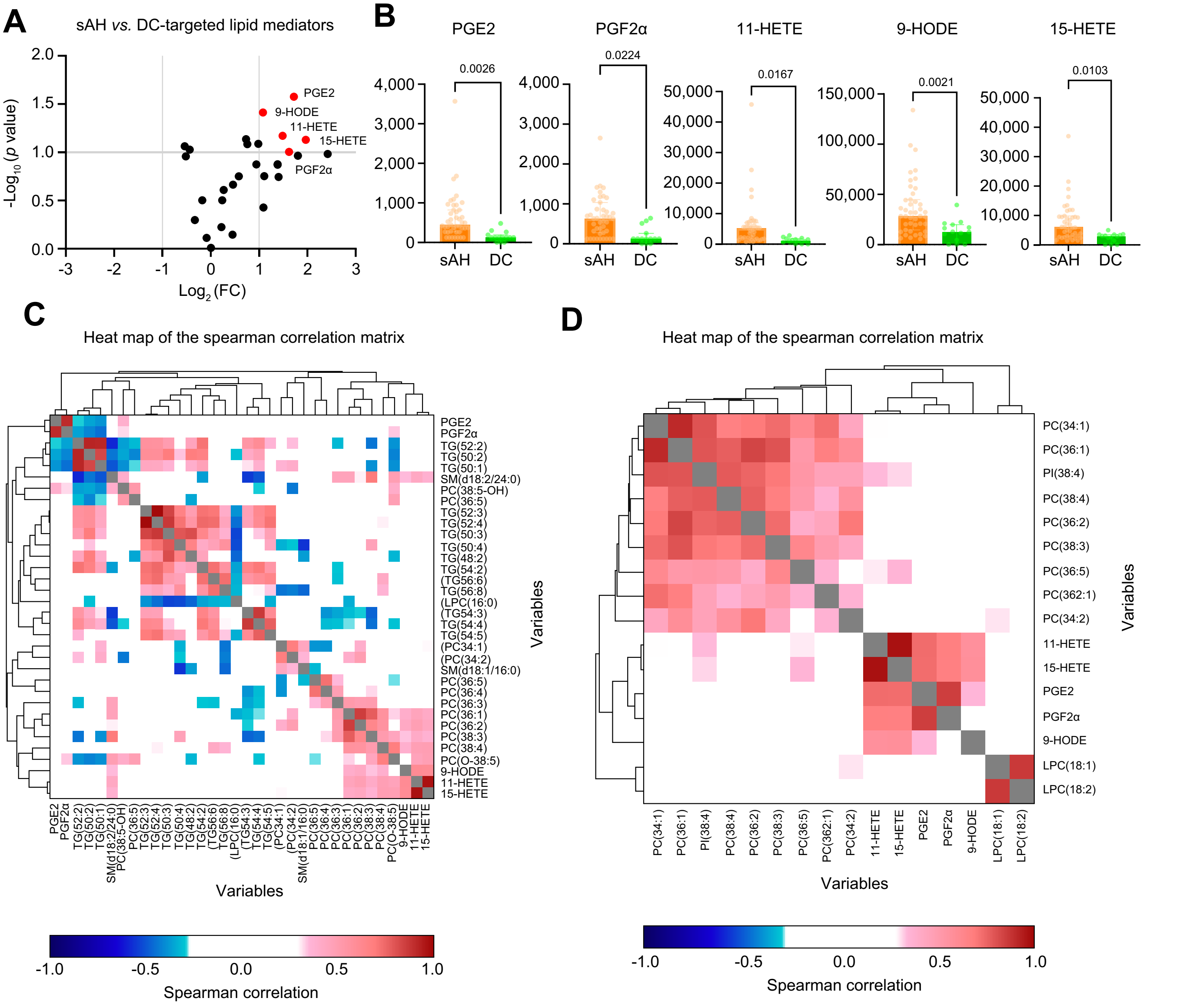

Fig. 3. Analyses of lipid mediators in the matched cohort (sAH n = 47; DC n = 15). (A) Volcano plots of each lipid mediators with respect to condition (sAH *vs.* DC). Red plot identifying variables above -1;1 $\log_2$(FC) threshold and 1 $-\log_{10}$(p value) threshold. (B) Univariable analysis of the lipid mediators identified in (A) with respect to the condition (sAH *vs.* DC): PGE2, p = 0.0026; PGF2α, p = 0.0224; 11-HETE, p = 0.0167; 9-HODE, p = 0.0021; and 15-HETE, p = 0.0103 (Mann-Whitney U test). (C) Correlation matrix (Spearman test) including lipid mediators identified in (A) and (B) with lipids with a VIP value ≥2 in positive ionisation mode. (D) Correlation matrix (Spearman test) including lipid mediators identified in (A) and (B) with lipids with a VIP value ≥2 in negative ionisation mode. DC, decompensated cirrhosis; sAH, severe alcohol-related hepatitis; VIP, variable projection of importance.

alcohol intoxication in patients with alcohol-related liver disease and non-alcohol-related fatty liver disease, LPC levels were decreased, suggesting an uptake by the liver contributing to caspase activation, endoplasmic reticulum stress, lipoapoptosis, and the development of alcohol-related steatohepatitis in humans.[28] LPC levels can also decrease through the activation of the LPC–ATX–LPA axis. We recently showed that in both the ACLF and acute liver failure (ALF) settings, this axis was associated with outcomes and that the modulation of LPA, acting through their specific receptor (lysophosphatidic acid receptor [LPAR]), has the potential to reverse the pro-restorative phenotype of circulating monocytes.[15,29] In the present lipidomic analysis, LPC(16:0), LPC(18:1), and LPC(18:2) were identified as key lipids differentiating sAH from DC. LPC(16:0) was negatively correlated with pro-inflammatory cytokines, WBC, and neutrophil count, as well as indicators of liver disease severity.

Sphingolipids are represented by ceramides and their precursors sphingomyelins as well as their phosphorylated derivatives sphingosine-1-phosphate (S1P) and ceramide-1-phosphate (C1P). Lysosphingolipids have been implicated in the regulation of a myriad of cell signals, particularly cell survival, adhesion, migration, and barrier integrity, which led to considering sphingolipid metabolism as a true rheostat of the inflammatory processes with pro-inflammatory and anti-inflammatory capacities.[6,30–32] Of note, the S1P–S1P receptor

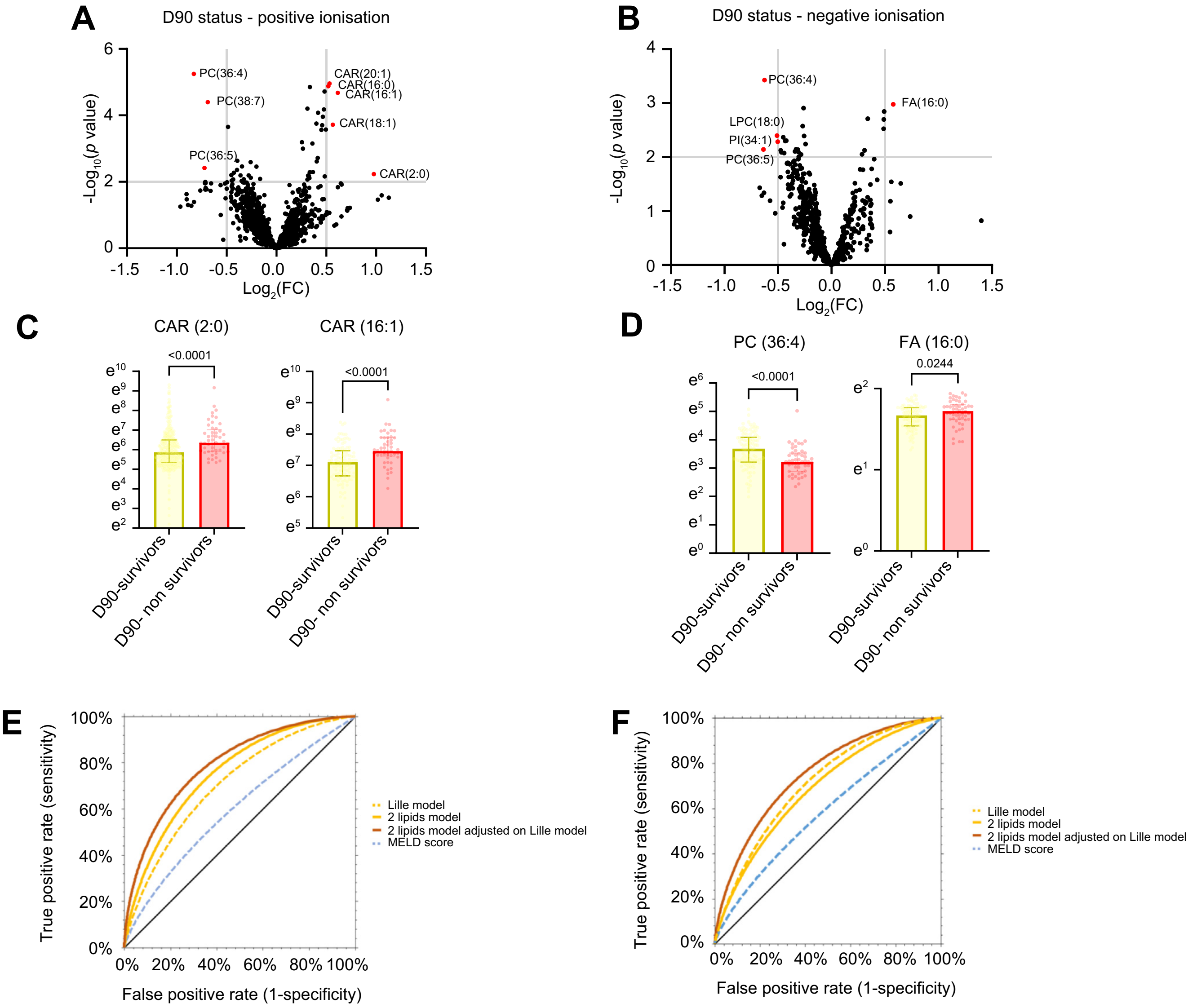

Model 1	Model 2	*p* value
2 lipids model: 0.75 (0.65-0.82)	MELD score: 0.59 (0.48-0.69)	0.02
2 lipids model: 0.75 (0.65-0.82)	Lille model: 0.71 (0.60-0.79)	0.50
2 lipids model: 0.75 (0.65-0.82)	2 lipids model adjusted on Lille score: 0.79 (0.70-0.90)	0.11
2 lipids model adjusted on Lille model: 0.79 (0.70-0.90)	MELD score: 0.59 (0.48-0.69)	0.001
2 lipids model adjusted on Lille model: 0.79 (0.70-0.90)	Lille model: 0.71 (0.60-0.79)	0.02

Model 1	Model 2	*p* value
2 lipids model: 0.69 (0.58-0.77)	MELD score: 0.59 (0.48-0.69)	0.08
2 lipids model: 0.69 (0.58-0.77)	Lille model: 0.71 (0.60-0.79)	0.73
2 lipids model: 0.69 (0.58-0.77)	2 lipids model adjusted on Lille score: 0.75 (0.65-0.82)	0.09
2 lipids model adjusted on Lille model: 0.75 (0.65-0.82)	MELD score: 0.59 (0.48-0.69)	0.004
2 lipids model adjusted on Lille model: 0.75 (0.65-0.82)	Lille model: 0.71 (0.60-0.79)	0.21

Fig. 4. Untargeted lipidomics in restricted to patients with sAH based on status on day 90 (survivors n = 106; non-survivors n = 51). (A) Volcano plots of all features in positive ionisation mode with respect to the D90 status (survivors *vs*. non-survivors). Red plot identifying features above the -0.5;0.5 $\log_2$(FC) and 2 -$\log_{10}$(*p* value according to the Mann-Whitney *U* test) thresholds that were further annotated based on raw chromatograms. (B) Volcano plots of all features of negative ionisation mode with respect to the D90 status (survivors *vs*. non-survivors). Red plot identifying features above the -0.5;0.5 $\log_2$(FC) and 2 -$\log_{10}$(*p* value according to the Mann-Whitney *U* test) thresholds that were further annotated based on raw chromatograms. (C) Univariable analyses of the lipids independently associated with the D90 status in Table 4 in positive ionisation mode: CAR(2:0), p <0.0001, and CAR(16:1), p <0.0001 (Mann-Whitney *U* test). (D) Univariable analyses of the lipids independently associated with the D90 status in Table S7 in negative ionisation mode: PC(36:4), p <0.0001, and FA(16:0), p = 0.0244. (E) Performance based on AUROC of the two-lipid model adjusted for the Lille model and MELD score alone, in differentiating D90 survivors from non-survivors in the sAH cohort in positive ionisation mode. Two-lipid model *vs*. MELD score: p = 0.02; two-lipid model *vs*. Lille model: p = 0.50; two-lipid model *vs*. two-lipid model adjusted for the Lille model: p = 0.11; two-lipid model adjusted for the Lille model *vs*. MELD score: p = 0.001; two-lipid model adjusted for the Lille model *vs*. Lille model: p = 0.02. (F) Performance based on AUROC of the two-lipid model adjusted for the Lille model and MELD score alone in differentiating D90 survivors from non-survivors in the sAH cohort in negative ionisation mode. Two-lipid model *vs*. MELD score: p = 0.08; two-lipid model *vs*. Lille model: p = 0.73; two-lipid model *vs*. two-lipid model adjusted for the Lille model: p = 0.09; two-lipid model adjusted for the Lille model *vs*. MELD score: p = 0.004; two-lipid model adjusted for the Lille model *vs*. Lille model: p = 0.21 (Z test).

Table 4. Univariable and multivariable logistic regression analyses of lipid species associated with D90 mortality in positive ionisation mode.

	Univariable analysis			Multivariable analysis		
**Covariant**	**OR**	**95% CI**	***p* value**	**OR**	**95% CI**	***p* value**
CAR(2:0)	1.86	1.11–3.16	<0.0001	2.51<br>3.02*	1.25–4.96<br>1.37–6.65*	0.008<br>0.005*
PC(36:5)	0.68	0.44–0.83	0.004	1.51	0.95–2.39	0.09
CAR(18:1)	1.68	1.25–2.14	<0.0001	1.08	0.39–2.98	0.88
PC(38:7)	0.44	0.28–0.73	0.0006	0.30	0.08–1.03	0.19
CAR(16:1)	1.81	1.15–2.89	<0.0001	2.21<br>1.81*	1.09–7.48<br>103–5.12*	0.009<br>0.03*
CAR(16:0)	1.56	1.06–2.12	0.0004	1.71	0.79–4.32	0.41
CAR(20:1)	1.48		0.0001	1.45	0.83–2.54	0.07
PC(36:4)	0.58	0.41–0.72	<0.0001	1.01	0.49–2.09	0.18

Effect size calculated per standard deviation increase. Independent associated observed for CAR(2:0): p = 0.008 and p = 0.005 after adjustment for the Lille model; CAR(16:1): p = 0.009 and p = 0.03 after adjustment for the Lille model. *After adjustment for the Lille model (13.78, 2.63–72.12, p = 0.003). CI, confidence interval; MELD, model for end-stage liver disease; OR, odds ratio.

(S1PR) pathway is a driver of multiple inflammatory diseases (*e.g.* multiple sclerosis, ulcerative colitis, and rheumatoid arthritis), and there is already a S1PR-targeted drug available.[30] In liver disease, this axis has been shown to promote liver fibrosis and to regulate liver regeneration, a key altered mechanism in sAH.[30,33] Although lower sphingolipid concentrations have been associated with a higher risk of death in sAH, S1P concentration has been shown to be reduced in patients with acute decompensation of cirrhosis and ACLF compared with healthy controls, suggesting an increased activation of the S1P–S1PR pathway.[14,16] Here, we observed that several sphingomyelins were part of the signature differentiating sAH from DC and also served as indicators of incident infection and AKI in the sAH population.

Our results suggest that acylcarnitines are enriched in the plasma of patients with sAH with a higher risk of death. Acylcarnitines are long-chain FAs activated in the mitochondria and their accumulation is a marker of mitochondrial dysfunction, which is a key feature observed in patients with sAH.[34,35] Indeed, after mitochondrial translocation, acylcarnitines are converted to acyl-CoA, which enters the fatty acid β-oxidation pathway. In the present study, CAR(16:1) correlated with ballooning, a marker of hepatocyte damage, and positively correlated with neutrophils infiltration and Mallory–Denk bodies as well as cell death markers (CK-18 M65 and M30 fragments), which were previously associated with steroid-responsiveness in sAH.[21] These results are in line with recently published data.[16] Fatty acid transport and β-oxidation have been shown to be impaired in hepatocytes from patients who underwent liver transplantation in the setting of sAH, suggesting an upstream accumulation of circulating acylcarnitines in patients that are likely to die.[16] Most acylcarnitines identified in the present work correlated with the MELD score, Glasgow score, and Lille model. Mitochondrial dysfunction has been shown to govern the immunometabolism in circulating leucocytes in ACLF.[36] Considering the impact of infection incidence in the natural history of patients with sAH and the evidence provided by the present study, therapeutic approaches aiming to resuscitate mitochondrial dysfunction should be further explored. Proof-of-concept studies have already been published in the setting of septic shock as well as in ACLF, where the modulation of anaplerotic reaction aiming to fuel tricarboxylic acid cycle has been demonstrated to improve mitochondrial function.[37,38] Such approaches should be investigated in the sAH population. Remarkably, we observed that acetylcarnitine (CAR(2:0)) was one of the two lipids independently associated with the D90 status in positive ionisation mode. CAR(2:0) may be influenced by bacterial translocation, as microbes are a major source of short-chain FAs.[39] Consequently, CAR(2:0) levels could reflect a combination of the intensity of bacterial translocation and mitochondrial dysfunction, both of which are key features of sAH.

Interestingly, we identified a specific lipoproteomic signature in patients with sAH compared with their matched controls with DC. Although it is known that the overall lipoprotein concentration decreases with the severity of the liver disease,[40,41] we reported here an increase in LDL features, commonly reported as exerting pro-inflammatory effects, in patients with sAH compared with those with DC. Such an increase was closely correlated with levels of circulating lysophospholipids and glycerophospholipids. This suggests that in addition to changes in the circulating lipidome, patients with sAH have uniquely impaired metabolic changes that could further participate in promoting systemic inflammation.

This study has several limitations. Firstly, the lipid analysis performed was untargeted and thus lacked the specificity of quantitative techniques targeting one or two lipid classes, providing only relative differences between the samples. An untargeted profiling approach, however, has the potential of discovering novel lipid species not previously described in this kind of disease. In addition, although we have some strong evidence that LPC–ATX–LPA pathway modulation plays an important role in the phenotype of circulating immune cells in ACLF, we have not been able to confirm these findings in the sAH setting in an independent cohort. Further work is needed to investigate the role of sphingolipids and acylcarnitines in sAH. However, in the context of the evidence for the role of sphingolipids and acylcarnitines in other diseases and the robustness of our bioinformatics approach, our study helps identify lipid classes that might play an important role in the key mechanisms that differentiate sAH from DC and lead to complications.

Taken together, the present study underlines the role of several PC/LPC species and sphingolipids in differentiating sAH from DC. These species were also associated with the risk of death, incident infection, and AKI in the sAH population. Considering the growing evidence on the role of

these lipids in the regulation of liver regeneration and immune function, they should be promptly explored for therapeutic potential. The acylcarnitine accumulation observed in patients with sAH and poorer prognosis could reflect the degree of mitochondrial dysfunction as well as gut translocation in these patients. Considering the place of mitochondrial dysfunction in the pathogenesis of the disease, prospective exploration of acylcarnitines pathways as a potential target to modulate this dysfunction is also urgently indicated.

Affiliations

[1]*Institute of Liver Studies, King's College Hospital London, London, UK;* [2]*Institute of Liver Studies, School of Immunology and Microbial Sciences, Faculty of Life Sciences and Medicine, King's College London, London, UK;* [3]*Department of Metabolism, Digestion and Reproduction, Division of Digestive Disease, Liver Unit, St Mary's and Hammersmith Hospitals, Imperial College London, London, UK Imperial College London, London, UK;* [4]*University of Rennes and Liver Department of Rennes University Hospital, Rennes, France;* [5]*Roger Williams Institute of Hepatology London, Foundation for Liver Research, London, UK;* [6]*Section of Bioanalytical Chemistry, Division of Systems Medicine, Department of Metabolism, Digestion and Reproduction, Imperial College London, London, UK*

Abbreviations

11/15-HETE, 11/15-hydroxyeicosatetraenoic acid; 9-HODE, 9-hydroxyoctadeca dienoic acid; ACLF, acute-on-chronic liver failure; AHHSS, alcoholic hepatitis histological scoring system; AKI, acute kidney injury; ALF, acute liver failure; ATX, autotaxin; AUROC, area under the receiver operating characteristic curve; C1P, ceramide-1-phosphate; CAR, carnitine; CD163, cluster of differentiation 163; CI, confidence interval; DC, decompensated cirrhosis; EGF, epidermal growth factor; FA, fatty acid; GAHS, Glasgow AH score; HGF, hepatocyte growth factor; IDPN, intermediate-density lipoprotein particle number; IFN, interferon; IGF, insulin-like growth factor; INR, international normalised ratio; L1PN, lipoprotein subclass 1 particle number; L3PN, lipoprotein subclass 3 particle number; L4PN, lipoprotein subclass 4 particle number; LBP, lipopolysaccharide-binding protein; LDPN, LDL particle number; LPA, lysophosphatidic acid; LPAR, lysophosphatidic acid receptor; LPC, lysophosphatidylcholine; MELD, model for end-stage liver disease; NGAL, neutrophil gelatinase-associated lipocalin; OPLS-DA, orthogonal partial least squares discriminant analysis; OR, odds ratio; PC, phosphocholine; PCA, principal components analysis; PD-1, programmed death-1; PD-L1, programmed death-ligand 1; PDGFA, platelet-derived growth factor subunit A; PGE2, prostaglandin E2; PGF2α, prostaglandin F2α; PI, phosphatidylinositol; S1P, sphingosine-1-phosphate; S1PR, S1P–S1P receptor; sAH, severe alcohol-related hepatitis; SL, sphingolipid; SM, sphingomyelin; TBPN, total blood particle number; TG, triglyceride; TGFβ1/2/3, transforming growth factor beta 1/2/3; TNFα, tumour necrosis factor alpha; TWEAK, TNF-like weak inducer of apoptosis; VEGF, vascular endothelial growth factor; VIP, variable projection of importance; WBC, white blood cell.

Financial support

This study was supported by the National Institute for Health and Care Research (NIHR) Imperial Biomedical Research Centre and the UKRI Medical Research Council (project reference: MR/X009904/1 and MR/R014019/1).

Conflicts of interest

The authors declare no conflicts of interest that pertain to this work.

Please refer to the accompanying ICMJE disclosure forms for further details.

Authors' contributions

Design of the study: FA, SA, MM, MT. Acquisition of data: FA, SA, FT, LDT, NV, VCP, MGR, NK, RG, AQ, AP, PM, SM, AC, EJ, MZ, RS, MM, ET, EH, MJM, MT. Statistical analysis: FA, MM. Drafting of the manuscript and critical review: FA, SA, FT, LDT, NV, VCP, MGR, NK, RG, AQ, AP, PM, SM, AC, EJ, MZ, RS, MM, ET, EH, MJM, MT.

Data availability statement

The data that support the findings of this study are available from the corresponding author upon reasonable request.

Supplementary data

Supplementary data to this article can be found online at https://doi.org/10.1016/j.jhepr.2025.101367.

References

Author names in bold designate shared co-first authorship

[1] Mathurin P, Thursz M. Endpoints and patient stratification in clinical trials for alcoholic hepatitis. J Hepatol 2019;70:314–318.

[2] Szabo G, Thursz M, Shah VH. Therapeutic advances in alcohol-associated hepatitis. J Hepatol 2022;76:1279–1290.

[3] Rattan P, Shah VH. Review article: current and emerging therapies for acute alcohol-associated hepatitis. Aliment Pharmacol Ther 2022;56:28–40.

[4] Bou Saleh M, Louvet A, Ntandja-Wandji LC, et al. Loss of hepatocyte identity following aberrant YAP activation: a key mechanism in alcoholic hepatitis. J Hepatol 2021;75:912–923.

[5] Louvet A, Thursz MR, Kim DJ, et al. Corticosteroids reduce risk of death within 28 days for patients with severe alcoholic hepatitis, compared with pentoxifylline or placebo—a meta-analysis of individual data from controlled trials. Gastroenterology 2018;155:458–468.e8.

[6] Artru F, McPhail MJW, Triantafyllou E, et al. Lipids in liver failure syndromes: a focus on eicosanoids, specialized pro-resolving lipid mediators and lysophospholipids. Front Immunol 2022;13:867261.

[7] Serhan CN. Pro-resolving lipid mediators are leads for resolution physiology. Nature 2014;510:92–101.

[8] Dennis EA, Norris PC. Eicosanoid storm in infection and inflammation. Nat Rev Immunol 2015;15:511–523.

[9] Zhou SL, Gordon RE, Bradbury M, et al. Ethanol up-regulates fatty acid uptake and plasma membrane expression and export of mitochondrial aspartate aminotransferase in HepG2 cells. Hepatology 1998;27:1064–1074.

[10] Zhong W, Zhao Y, Tang Y, et al. Chronic alcohol exposure stimulates adipose tissue lipolysis in mice: role of reverse triglyceride transport in the pathogenesis of alcoholic steatosis. Am J Pathol 2012;180:998–1007.

[11] Berk PD, Zhou S, Bradbury MW. Increased hepatocellular uptake of long chain fatty acids occurs by different mechanisms in fatty livers due to obesity or excess ethanol use, contributing to development of steatohepatitis in both settings. Trans Am Clin Climatol Assoc 2005;116:335–344. ; discussion 345.

[12] **Thiele M**, **Suvitaival T**, **Trošt K**, et al. Sphingolipids are depleted in alcohol-related liver fibrosis. Gastroenterology 2023;164:1248–1260.

[13] López-Vicario C, Checa A, et al. Targeted lipidomics reveals extensive changes in circulating lipid mediators in patients with acutely decompensated cirrhosis. J Hepatol 2020;73:817–828.

[14] **Clària J**, **Curto A**, **Moreau R**, et al. Untargeted lipidomics uncovers lipid signatures that distinguish severe from moderate forms of acutely decompensated cirrhosis. J Hepatol 2021;75:1116–1127.

[15] Trovato FM, Zia R, Napoli S, et al. Dysregulation of the lysophosphatidylcholine/autotaxin/lysophosphatidic acid axis in acute-on-chronic liver failure is associated with mortality and systemic inflammation by lysophosphatidic acid-dependent monocyte activation. Hepatology 2021;74:907–925.

[16] Gao B, Argemi J, Bataller R, Schnabl B. Serum acylcarnitines associated with high short-term mortality in patients with alcoholic hepatitis. Biomolecules 2021;11:281.

[17] Thursz MR, Richardson P, Allison M, et al. Prednisolone or pentoxifylline for alcoholic hepatitis. N Engl J Med 2015;372:1619–1628.

[18] Wolfer AM, Gaudin M, Taylor-Robinson SD, et al. Development and validation of a high-throughput ultrahigh-performance liquid chromatography-mass spectrometry approach for screening of oxylipins and their precursors. Anal Chem 2015;87:11721–11731.

[19] Plasma analysis | serum analysis | quantification. Bruker 2022. https://www.bruker.com/en/products-and-solutions/mr/nmr-clinical-research-solutions/b-i-quant-ps.html. [Accessed 14 July 2022].

[20] Ku NO, Strnad P, Bantel H, et al. Keratins: biomarkers and modulators of apoptotic and necrotic cell death in the liver. Hepatology 2016;64:966–976.

[21] Atkinson SR, Grove JI, Liebig S, et al. In severe alcoholic hepatitis, serum keratin-18 fragments are diagnostic, prognostic, and theragnostic biomarkers. Am J Gastroenterol 2020;115:1857–1868.

[22] Altamirano J, Miquel R, Katoonizadeh A, et al. A histologic scoring system for prognosis of patients with alcoholic hepatitis. Gastroenterology 2014;146:1231–1239. e1-6.
[23] Yun M-R, Okajima F, Im D-S. The action mode of lysophosphatidylcholine in human monocytes. J Pharmacol Sci 2004;94:45–50.
[24] Kume N, Cybulsky MI, Gimbrone MA. Lysophosphatidylcholine, a component of atherogenic lipoproteins, induces mononuclear leukocyte adhesion molecules in cultured human and rabbit arterial endothelial cells. J Clin Invest 1992;90:1138–1144.
[25] Murch O, Collin M, Sepodes B, et al. Lysophosphatidylcholine reduces the organ injury and dysfunction in rodent models of gram-negative and gram-positive shock. Br J Pharmacol 2006;148:769–777.
[26] Han MS, Park SY, Shinzawa K, et al. Lysophosphatidylcholine as a death effector in the lipoapoptosis of hepatocytes. J Lipid Res 2008;49:84–97.
[27] Mota M, Banini BA, Cazanave SC, et al. Molecular mechanisms of lipotoxicity and glucotoxicity in nonalcoholic fatty liver disease. Metabolism 2016;65:1049–1061.
[28] **Israelsen M**, **Kim M**, Suvitaival T, et al. Comprehensive lipidomics reveals phenotypic differences in hepatic lipid turnover in ALD and NAFLD during alcohol intoxication. JHEP Rep 2021;3:100325.
[29] Trovato FM, Zia R, Artru F, et al. Lysophosphatidylcholines modulate immunoregulatory checkpoints in peripheral monocytes and are associated with mortality in people with acute liver failure. J Hepatol 2023;78:558–573.
[30] Cartier A, Hla T. Sphingosine 1-phosphate: lipid signaling in pathology and therapy. Science 2019;366:eaar5551.
[31] Cuvillier O, Pirianov G, Kleuser B, et al. Suppression of ceramide-mediated programmed cell death by sphingosine-1-phosphate. Nature 1996;381:800–803.
[32] Tan ST, Ramesh T, Toh XR, et al. Emerging roles of lysophospholipids in health and disease. Prog Lipid Res 2020;80:101068.
[33] Sato M, Ikeda H, Uranbileg B, et al. Sphingosine kinase-1, S1P transporter spinster homolog 2 and S1P2 mRNA expressions are increased in liver with advanced fibrosis in human. Sci Rep 2016;6:32119.
[34] McGill MR, Li F, Sharpe MR, et al. Circulating acylcarnitines as biomarkers of mitochondrial dysfunction after acetaminophen overdose in mice and humans. Arch Toxicol 2014;88:391–401.
[35] Bataller R, Arab JP, Shah VH. Alcohol-associated hepatitis. N Engl J Med 2022;387:2436–2448.
[36] Zhang IW, Curto A, López-Vicario C, et al. Mitochondrial dysfunction governs immunometabolism in leukocytes of patients with acute-on-chronic liver failure. J Hepatol 2022;76:93–106.
[37] Korf H, du Plessis J, van Pelt J, et al. Inhibition of glutamine synthetase in monocytes from patients with acute-on-chronic liver failure resuscitates their antibacterial and inflammatory capacity. Gut 2019;68:1872–1883.
[38] Reizine F, Grégoire M, Lesouhaitier M, et al. Beneficial effects of citrulline enteral administration on sepsis-induced T cell mitochondrial dysfunction. Proc Natl Acad Sci U S A 2022;119:e2115139119.
[39] McCrory C, Lenardon M, Traven A. Bacteria-derived short-chain fatty acids as potential regulators of fungal commensalism and pathogenesis. Trends Microbiol 2024;32:1106–1118.
[40] Cicognani C, Malavolti M, Morselli-Labate AM, et al. Serum lipid and lipoprotein patterns in patients with liver cirrhosis and chronic active hepatitis. Arch Intern Med 1997;157:792–796.
[41] Trieb M, Rainer F, Stadlbauer V, et al. HDL-related biomarkers are robust predictors of survival in patients with chronic liver failure. J Hepatol 2020;73:113–120.

Keywords: Alcohol-related hepatitis; Acute-on-chronic liver failure; Cirrhosis; Lipidomics; Glycerophospholipids; Lysophospholipids; Mitochondrial dysfunction; Acylcarnitines.
Received 23 May 2024; received in revised form 7 February 2025; accepted 17 February 2025; Available online 1 March 2025

Journal of Hepatology, Volume *7*

Supplemental information

Lipidomics-based plasma signature of alcohol-related hepatitis linked to short-term mortality

Florent Artru, Stephen Atkinson, Francesca Trovato, Luke D. Tyson, Vishal C. Patel, Nikhil Vergis, Noora Kano, Robert Goldin, Alberto Quaglia, Alexandros Pechlivanis, Phillip Morgan, Salma Mujib, Anna Cavazza, Ellen Jerome, Marc Zentar, Roosey Sheth, Maura Morrison, Evangelos Triantafyllou, Elaine Holmes, María Gómez-Romero, Mark J. McPhail, and Mark Thursz

Lipidomics-based plasma signature of alcohol-related hepatitis linked to short-term mortality

Florent Artru, Stephen Atkinson, Francesca Trovato, Luke D. Tyson, Vishal C. Patel, Nikhil Vergis, Noora Kano, Robert Goldin, Alberto Quaglia, Alexandros Pechlivanis, Phil Morgan, Salma Mujib, Anna Cavazza, Ellen Jerome, Marc Zentar, Roosey Sheth, Maura Morrison, Evangelos Triantafyllou, Elaine Holmes, María Gómez-Romero, Mark J McPhail, Mark Thursz

Table of contents

Supplementary methods 2

Supplementary figures 9

Supplementary tables 29

Supplementary references 36

Supplementary methods

Definition

Incident acute kidney injury (AKI) was defined at D7 by an increase in serum creatinine to ≥1.5 times the patients' assumed baseline creatinine; an increase in creatinine to ≥26.5 µmol/L (0.3 mg/dL) above the baseline creatinine; or the initiation of renal replacement therapy. Patients with an AKI at recruitment (D0) were excluded; as we did not have pre-randomisation or historical values for patients' baseline creatinine available, historical baseline creatinine was assumed to be the lowest creatinine recorded during the STOPAH trial follow-up (up to day 365), discarding results obtained during RRT. Incident infection was defined as those that occurred after the start of treatment.

Untargeted lipidomics

Sample preparation

1. Thawing: Specimen were removed from low temperature storage (-80ºC) and allowed to thaw completely at 4°C.
2. Subsampling: Each thawed specimen was split by first brief vortexing of the original sample immediately followed by removal of the required volume of sample to a 2 mL eppendorf tube using an appropriate pipette and individual single-use tips to prevent sample cross contamination.
3. Randomisation: Specimen were randomised and given a new sequential numeric processing code prior to preparation. This is done to avoid batching in subsequent steps.
4. Protein precipitation: Serum samples were prepared for UHPLC-MS lipid analysis by isopropanol (IPA) protein precipitation (1). IPA was placed in the fridge a day prior to precipitation use. Cold IPA (1:3 (v/v) sample:solvent) was added to serum, vortexed and incubated at -20˚C for 2 hours. b) Samples were centrifuged at 4°C for 10 minutes at 130 000 rpm. c) The supernatant of each specimen was removed to a glass insert in a vial using an appropriate pipette. d) The volume removed should equal 75% of the original specimen volume to avoid dislodging of or contamination with pelleted material. e) A "blank sample" was also prepared by using high purity water instead of the serum and following the exact same procedure.
5. Composite quality control (QC) sample generation. a) An equal volume of each sample was removed from each Eppendorf tube using an appropriate micropipette. b) The removed sample volumes were pooled to create a single QC. c) The composite QC sample is mixed thoroughly by inversion and vortexing and distributed among several vials.

Sample analysis by UHPLC-MS

Sample analysis was performed on an Acquity UPLC system (Waters Ltd. Elstree, U.K.) coupled to a Q-TOF Premier mass spectrometer (Waters Ltd., Manchester, U.K.). Mass spectra were acquired in both positive and negative ionisation electrospray modes.

The separation conditions have been previously established (2). Gradient elution was performed using a CSH C18 (1.7µm, 2.1 x 100mm) column (Waters Corporation, Milford, U.S.A.) kept at 55ºC. The injection volume was 5 and 10 µL for the positive and negative ion mode, respectively. The mobile phases consisted of 0.1% formic acid (v/v) and 10 mM ammonium formate in 60:40 (v:v) acetonitrile/water (A) and 0.1% formic acid (v/v) and 10 mM ammonium formate in 90:10 (v:v) IPA/acetonitrile (B) at a flow rate of 0.4 mL/min.

The MS parameters were set as follow: capillary voltage, 3 kV (ESI+) and 2.5 kV (ESI-); sample cone voltage, 30 V (ESI+) and 25 V (ESI-); source temperature 120ºC; desolvation temperature, 400ºC; desolvation gas flow, 800 L/h and cone gas flow, 25 L/h. For mass accuracy, a 0.2 ng/µL leucine enkephalin solution (m/z 556.2771 in positive ion mode, m/z 554.2615 in negative ion mode) at 20 µL/min was used as the lock mass. Data were collected in centroid mode with a scan range of 50-1200 m/z, with lock mass scans collected every 30 s and averaged over 3 scans to perform mass correction.

Masslynx v4.1 software (Waters, Manchester, U.K.) was used for data acquisition and visual inspection. Feature extraction was performed in XCMS (within R) (3). The centWave algorithm was used for peak picking, while the peak grouping step was performed with the density method. Lipid species tentative annotations were based on accurate *m/z* values, isotopic patterns and retention time, by matching the accurate mass of the molecular ion to reference spectra from LIPID MAPS (https://www.lipidmaps.org/) and human metabolome (HMDB; https://hmdb.ca/) publicly available databases. Some of the annotations had already been reported using the same chromatographic method (4).

Lipid mediators targeted analysis [5]

Sample preparation

Analytes were extracted using solid phase extraction (SPE) in 96-well plate format (Oasis MAX µElution plate from Waters (Milford, MA)). Briefly, 20 µL of IS working solution and 30 µL of 2% formic acid in water were added to 100 µL of serum sample. Samples were transferred into the SPE plate after conditioning, and the lipid mediators and PUFA were eluted with four times 25 µL of MeOH with 2% formic acid. The elution fraction was evaporated under nitrogen and the residues reconstituted in 120 µL of methanol/water 1:1 (v/v).

Sample analysis

Sample analysis was performed on an Acquity UPLC system (Waters Ltd. Elstree, U.K.) coupled to a TQ-S mass spectrometer (Waters Ltd., Manchester, U.K.) operated in the negative ionisation electrospray mode. Separation was performed with a Waters HSS T3 UPLC column (100 mm × 1 mm, 1.8 µm) column maintained at 40 °C. The mobile phases consisted of 0.1% formic acid in water (A) and 0.1% formic acid in acetonitrile (B), at a flow rate of 0.14 mL/min. The injection volume was 5 µL and a postcolumn infusion of acetonitrile with 37% formaldehyde 3:1 (v/v) at a flow rate of 5 µL/min was added. The source parameters were as follows: capillary voltage was set at 2.5 kV, cone voltage 10-40 V depending on the analyte, source temperature 150 °C, desolvation temperature 500 °C, desolvation

gas flow 900 L/h and cone gas flow 150 L/h. Analyses were performed in MRM mode, with dwell time, cone voltage and collision energy optimised for each analyte.
Peak detection, integration and quantification were performed using the TargetLynx within Masslynx 4.1 software.

Lipoprotein assays [6,7]

Serum stored at -80°C was thawed at 4°C then centrifuged at 12000 g at 4°C for 5 min, before 350 µL of each sample was transferred to an Eppendorf. This was then mixed with 350 µL of serum buffer (0.075 M NaH2PO4 - 5.32g NaH2PO4 dissolved in 380 mL ultrapure water, with 0.4 g 3-trimethyl-silyl-[2,2,3,3-2H4] propionic acid, sodium salt 98 atom % D, 5 mL of 4% NaN3 aqueous solution, and 100 mL of D2O sequentially added before pH was adjusted to 7.4 with HCl/ NaOH and the solution was made up to 500 mL with water). The mixture of sample and serum buffer was centrifuged at 12000g at 4°C for 5min, then 600 µL was loaded into a SampleJet NMR tube (Bruker, USA). All reagents were NMR grade, Sigma-Aldrich, USA.
Samples were run using the SampleJet loading system on a 600MHz Avance III NMR spectrometer (Bruker Biospin, USA) with a BBI 600 MHz 5-mm Z gradient probe NMR detector and automated tuning and matching unit (Bruker Biospin, USA). The temperature was calibrated so that serum samples were run at exactly 300 K. They were run using an optimised machine protocol described previously. Briefly, 1H NMR spectra were measured using a standardised water suppression pulse sequence, NOESY-presat, as well as by a standardised Carr-Purcell-Meiboom-Gill (CPMG) spin-echo sequence with presaturation, and Bruker J-resolved pulse sequence and diffiusion-filtered sequence 303. The spectra generated by these standardised sequences was then be analysed using Bruker's propriety B.I. Methods Package 2.0 which quantifies a lipoprotein subclass panel with 114 parameters (B.I. LISA). Analytes quantified by B.I. LISA are listed below. These metabolites, molecules and lipoproteins can be analysed directly. The spectra (e.g. CPMG) can be also be analysed, and discriminatory peaks identified if possible.

Abbreviations: VLDL = Very Low Density Lipoprotein; IDL = Intermediate Density Lipoprotein; LDL = Low Density Lipoprotein; HDL = High Density Lipoprotein. Subfractions 1-6 are numbered according to increasing density, as reproduced below.

Densities (in kg/L) of Lipoprotein Main Fractions:

VLDL	IDL	LDL	HDL
0.950 - 1.006	1.006 - 1.019	1.019 - 1.063	1.063 - 1.210

Density of the Very Low Density Lipoprotein Subfractions:

5 subfractions VLDL-1 ... VLDL-5, numbering according to increasing density.
Subfractions properties are specified in [1]

Densities (in kg/L) of Low Density Lipoprotein Subfractions:

LDL-1	LDL-2	LDL-3	LDL-4	LDL-5	LDL-6
1.019 - 1.031	1.031 - 1.034	1.034 - 1.037	1.037 - 1.040	1.040 - 1.044	1.044 - 1.063

Densities (in kg/L) of High Density Lipoprotein Subfractions:

HDL-1	HDL-2	HDL-3	HDL-4
1.063 - 1.100	1.100 - 1.112	1.112 - 1.125	1.125 - 1.210

- Cholesterol
- LDL-cholesterol
- HDL-cholesterol
- Apo-A1

- Apo-A2
- Apo-B100
- LDL-cholesterol/ HDL-cholesterol
- Apo-B100/ Apo-A1
- Total Particle Number
- VLDL Particle Number
- IDL Particle Number
- LDL Particle Number
- LDL-1 Particle Number
- LDL-2 Particle Number
- LDL-3 Particle Number
- LDL-4 Particle Number
- LDL-5 Particle Number
- LDL-6 Particle Number
- VLDL triglycerides
- IDL triglycerides
- LDL triglycerides
- HDL triglycerides
- VLDL cholesterol
- IDL cholesterol
- LDL cholesterol
- HDL cholesterol
- VLDL free cholesterol
- IDL free cholesterol
- LDL free cholesterol
- HDL free cholesterol
- VLDL phospholipids
- IDL phospholipids
- LDL phospholipids
- HDL phospholipids
- HDL Apo-A1
- HDL Apo-A2
- VLDL Apo-B
- IDL Apo-B
- LDL Apo-B
- VLDL-1 triglyceride
- VLDL-2 triglyceride
- VLDL-3 triglyceride
- VLDL-4 triglyceride
- VLDL-5 triglyceride
- VLDL-1 cholesterol
- VLDL-2 cholesterol
- VLDL-3 cholesterol
- VLDL-4 cholesterol
- VLDL-5 cholesterol
- VLDL-1 free cholesterol
- VLDL-2 free cholesterol
- VLDL-3 free cholesterol

- VLDL-4 free cholesterol
- VLDL-5 free cholesterol
- VLDL-1 phospholipids
- VLDL-2 phospholipids
- VLDL-3 phospholipids
- VLDL-4 phospholipids
- VLDL-5 phospholipids
- LDL-1 triglyceride
- LDL-2 triglyceride
- LDL-3 triglyceride
- LDL-4 triglyceride
- LDL-5 triglyceride
- LDL-6 triglyceride
- LDL-1 cholesterol
- LDL-2 cholesterol
- LDL-3 cholesterol
- LDL-4 cholesterol
- LDL-5 cholesterol
- LDL-6 cholesterol
- LDL-1 free cholesterol
- LDL-2 free cholesterol
- LDL-3 free cholesterol
- LDL-4 free cholesterol
- LDL-5 free cholesterol
- LDL-6 free cholesterol
- LDL-1 phospholipids
- LDL-2 phospholipids
- LDL-3 phospholipids
- LDL-4 phospholipids
- LDL-5 phospholipids
- LDL-6 phospholipids
- LDL-1 Apo-B
- LDL-2 Apo-B
- LDL-3 Apo-B
- LDL-4 Apo-B
- LDL-5 Apo-B
- LDL-6 Apo-B
- HDL-1 triglyceride
- HDL-2 triglyceride
- HDL-3 triglyceride
- HDL-4 triglyceride
- HDL-1 cholesterol
- HDL-2 cholesterol
- HDL-3 cholesterol
- HDL-4 cholesterol
- HDL-1 free cholesterol
- HDL-2 free cholesterol
- HDL-3 free cholesterol

- HDL-4 free cholesterol
- HDL-1 phospholipids
- HDL-2 phospholipids
- HDL-3 phospholipids
- HDL-4 phospholipids
- HDL-1 Apo-A1
- HDL-2 Apo-A1
- HDL-3 Apo-A1
- HDL-4 Apo-A1
- HDL-1 Apo-A2
- HDL-2 Apo-A2
- HDL-3 Apo-A2
- HDL-4 Apo-A2

Cytokines, chemokines, immune and renal markers analyses

All reagents used in this method were purchased from MSD, Rockville, USA. Kits were combined in a customised manner. The kits purchased were: R-PLEX Human HGF Antibody Set; R-PLEX Human EGF Antibody Set; R-PLEX Human CD163 Antibody Set; R-PLEX Human IGF-1 Antibody Set; R-PLEX Human LBP Antibody Set; R-PLEX Human NGAL/LCN2 Antibody Set; R-PLEX Human TWEAK/TNFSF12 Antibody Set; R-PLEX Human Cystatin C Antibody Set; U-PLEX Custom Biomarker (hu) Assays (U-PLEX Human IFN-γ, U-PLEX Human IL-1α, U-PLEX Human IL-1β, U-PLEX Human IL-6, U-PLEX Human IL-10, U-PLEX Human IL-22, U-PLEX Human IL-23, U-PLEX Human TNF-α. U-PLEX Human VEGF-A, Open Spots – 1 [to bind R-PLEX antibody]); U-PLEX Custom Immuno-Oncology Grp 1 (hu) Assays (U-PLEX Human IL-1RA; U-PLEX Human IL-8; U-PLEX Human IL-18; U-PLEX Human PD1 (Epitope 2); U-PLEX Human PD-L1 (Epitope 1); Open Spots – 4). Addition 4-spot plates and Diluent 3, 10, 43 and 100 were purchased. Samples were run on 96-well plates, with eight-point calibration curves run in duplicate after serial dilution in 96 well plates using diluent 100. The 10-plex was run with neat sample, the 9-plex with 1:10 and the 3-plex with 1:3000 dilutions.

Statistical analyses

Principal components analysis (PCA) was performed to visualise any inherent clustering and identify outliers (SIMCA v16.0). OPLS-DA was performed to maximise modelling of class differences while minimising variability unrelated to class. The R^2 value was calculated to give a measure of the goodness-of-fit or amount of variability explained by the model. A cross-validated Q2 statistic (leave-one-out algorithm) was calculated as a quantitative measure of the predictability of the model for the Y variable. The cross-validated analysis of-variance (CV-ANOVA) statistic corresponds to a null-hypothesis of equal predictive residuals between the models under investigation ($p<0.05$ suggests the model is superior to one chosen at random). Nine hundred ninety-nine permutation tests in the OPLS-DA were

performed to validate the models. Area under the receiving operating systems (AUROC) were built to evaluate the performance of the model to predict condition. S-plot loadings (displaying correlation versus covariance of spectral variables) and variable importance of the projection (VIP) tests were used to determine the features contributing to class separation. Features with a VIP value ≥ 2 were analysed on raw chromatograms and mass spectra for annotation using cross analyses of LIPID MAPS (https://www.lipidmaps.org/) and human metabolome (HMDB; https://hmdb.ca/) online databases. If the OPLS-DA model was not valid (or a low number of targeted metabolites formed the basis of the method), a volcano plot based on log 2-fold-change (log2[FC]) as X axis and -log10 of P-value of t-test (-log10[P-value]) as Y axis was built. Thresholds of -0.5 and 0.5 of log2(FC) and 2 of -log10(P-value) were set to identify features significantly associated with the condition. These features were analysed on raw chromatograms and mass spectra as performed in those identified from OPLS-DA models. After annotation, univariable and stepwise multivariable logistic regression was performed. Lipids with p ≤0.01 were entered in the multivariable analysis to mitigate the risk of model overfitting based on an appropriate balance between the number of events and the number of variables. Adjustments on MELD score to differentiate between sAH and cirrhosis first, and Lille score between 90-day survivors and non-survivors in the sAH group were performed as an internal control. To obtain a probability score ranging from 0 to 1, the R function obtained by the forward logistic regression function combining lipids with a p-value ≤0.05 was inserted in the following formula: 1/(1 + Exp(−R)). This approach has been previously described for the development of the Lille Model (8). Performance of lipids-based models were compared to reference scores using AUROC curves and the z test (9). Threshold of -1 and -1 of log2(FC) and 1 of -log10(P-value) was set for lipid mediators analyses to increase sensitivity as none reached -log10 (P-value) threshold of 2. Missing data were not inputted. T-test, one-way ANOVA/Kruskal-Wallis, Pearson/Spearman's correlations and AUROC were calculated with NCSS v2022 (NCSS, LLC) or GraphPad Prism v9.0 (GraphPad Software, San Diego, CA).

Supplementary figures

Supplementary figure 1

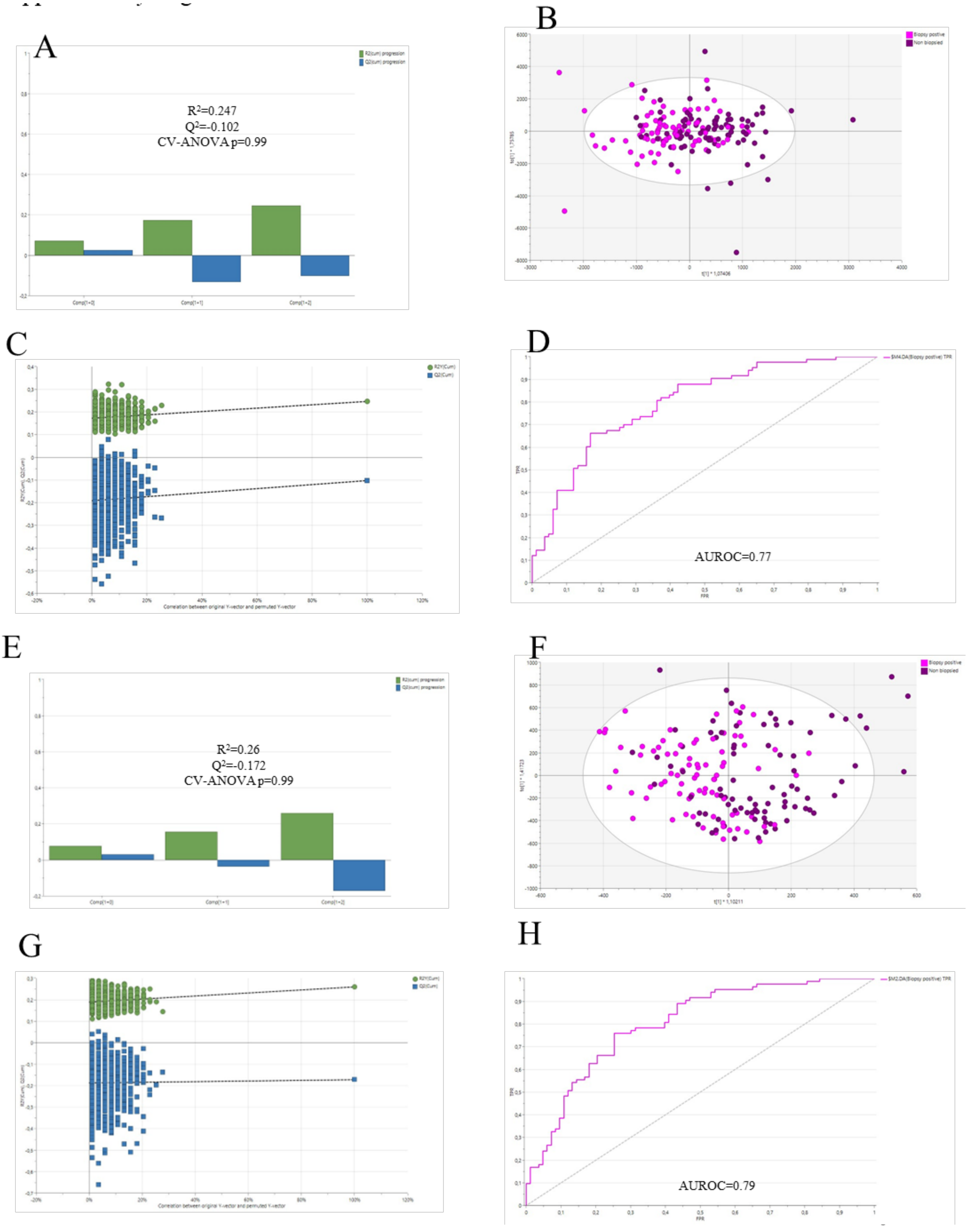

Legend to Fig. S1: Analyses of untargeted lipidomics between patients with sAH with positive features of sAH (n=76) and patients who did not undergo liver biopsy (n=83). A. Summary of fit of the OPLS-DA model in positive ionisation mode with 1+2+0 component: R^2=0.247, Q^2= -0.102, CV-ANOVA p=0.99. B. Plot of the positive ionisation mode model (biopsy positive patients in rose, non-biopsied patients in purple). C. Permutation test demonstrating the non-validity of the positive ionisation mode model. D. AUROC of the positive ionisation mode model. E Summary of fit of the model in negative ionisation mode with 1+2+0 component: R^2=0.26, Q^2= -0.172, CV-ANOVA p=0.99. F. Plot of the negative ionisation mode model (biopsy positive patients in rose, non-biopsied patients in purple). G. Permutation test demonstrating the non-validity of the negative ionisation mode model. H. AUROC of the negative ionisation mode model.

Supplementary figure 2

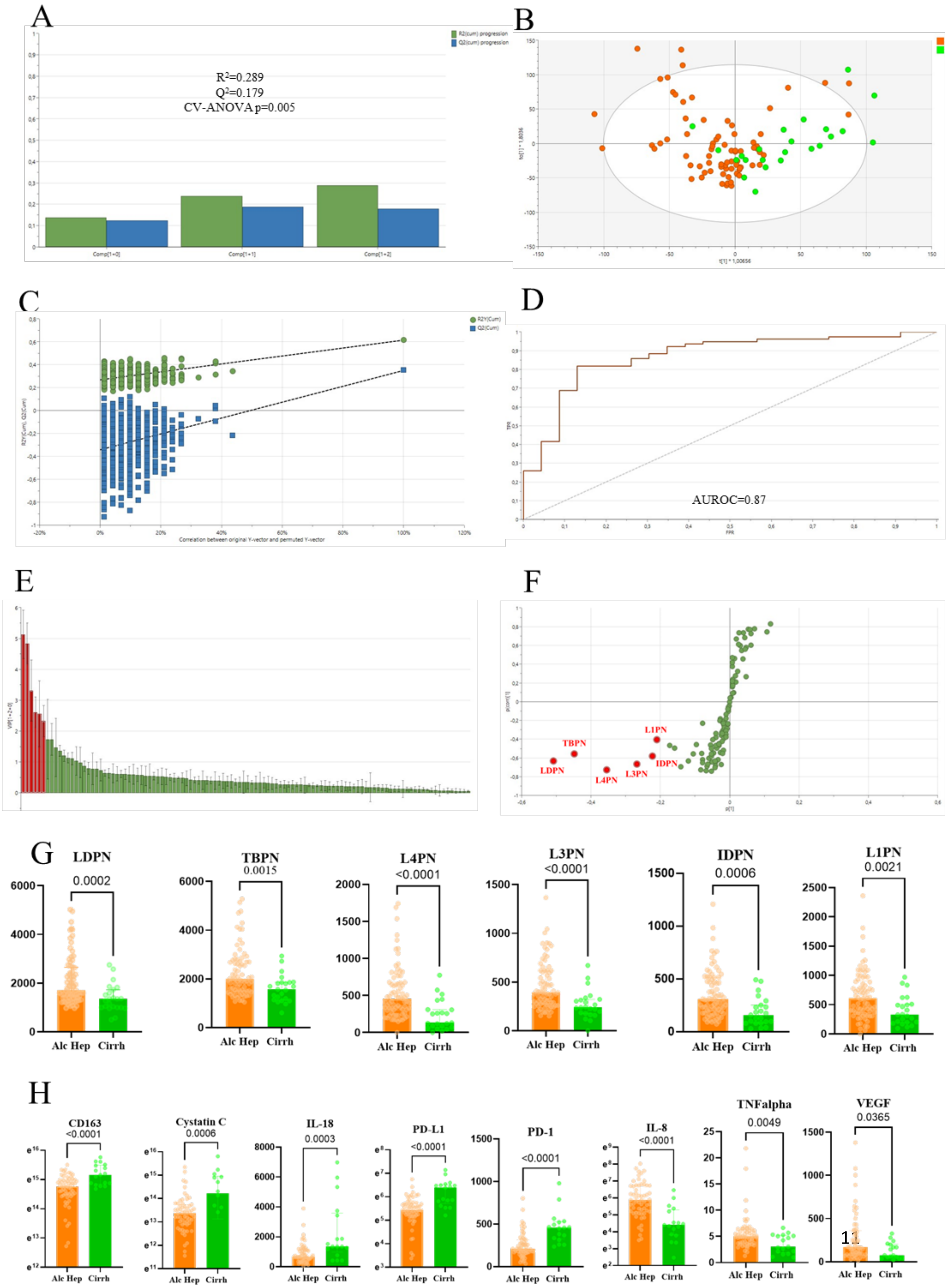

Legend to Fig. S2: Analyses of lipoprotein assay through 1H-NMR spectroscopy in patients with sAH (n=78) matched to patients with cirrhosis (n=23) on bilirubin level A. Summary of fit of the model in positive ionisation mode with 1+2+0 component: R^2=0.289, Q^2= 0.179, CV-ANOVA=0.005. B. Plot of the model with patients with sAH in orange and patients with DC in green. C. Permutation test demonstrating the non-validity of the model. D. AUROC of the model. E VIP plot of the OPLS-DA model: each bar in red represents a variable with VIP value ≥ 2. F. S-plot of the positive ionisation mode model: each variable is plotted; variables in red are those with VIP value ≥ 2. G. Univariate analyses of the six lipoproteins with a VIP value ≥ 2. H. Univariate analyses of the eight cytokines with different concentrations between patients with sAH and patients with DC.

Supplementary figure 3

A

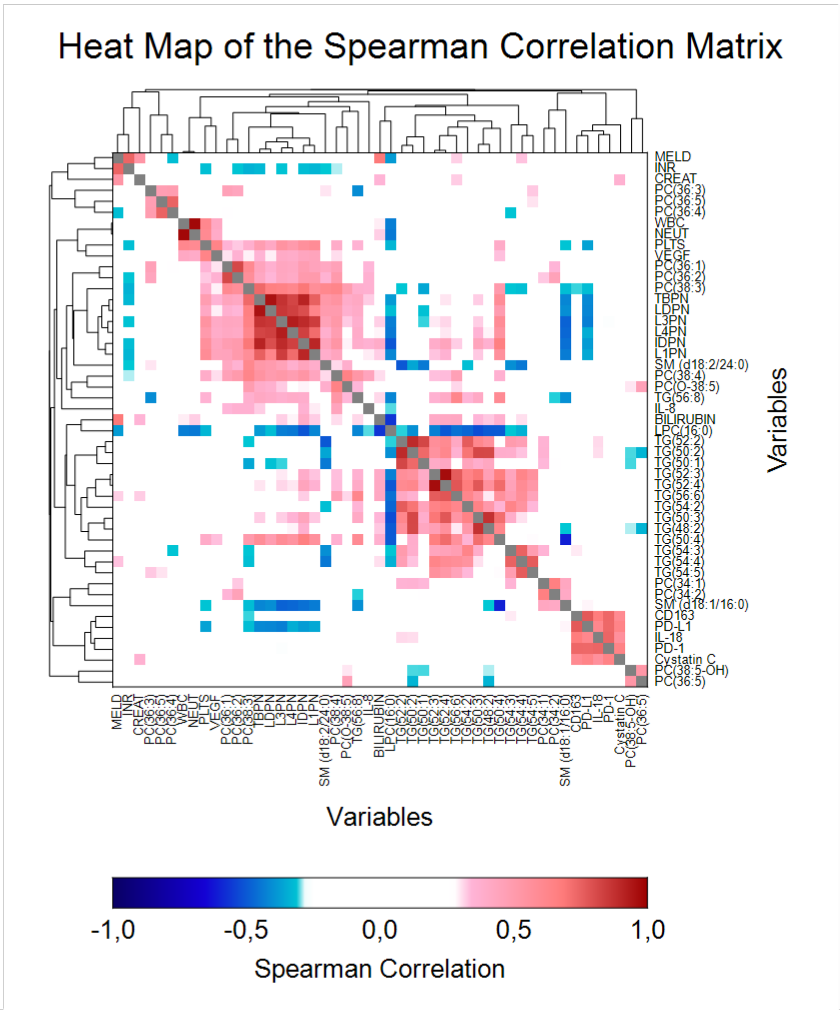

B

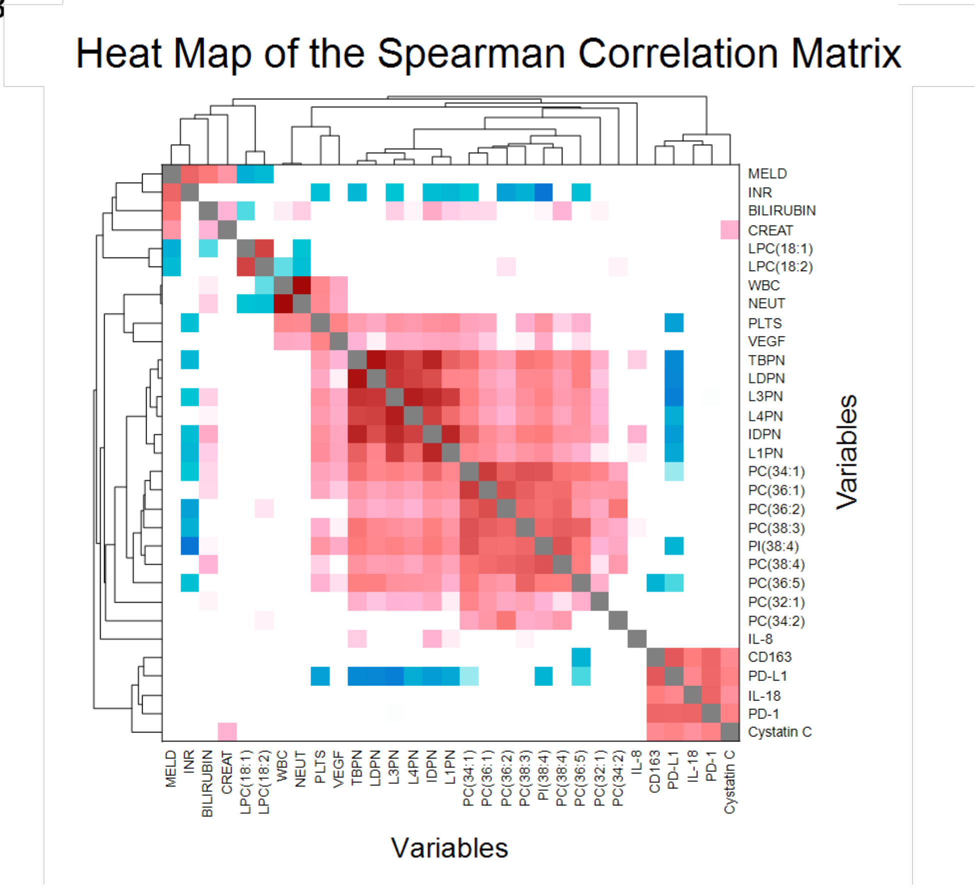

Legend to Fig. S3: Correlation matrix in in patients with sAH (n=78) matched to patients with DC (n=23) on bilirubin level based on Spearman's test between A. clinical, laboratory, lipids identified in positive ionisation mode with a VIP value ≥ 2, lipoproteins and cytokines identified in Fig. S2. B. clinical, laboratory, lipids identified in negative ionisation mode with a VIP value ≥ 2, lipoproteins and cytokines identified in Fig. S2.

Supplementary figure 4

A

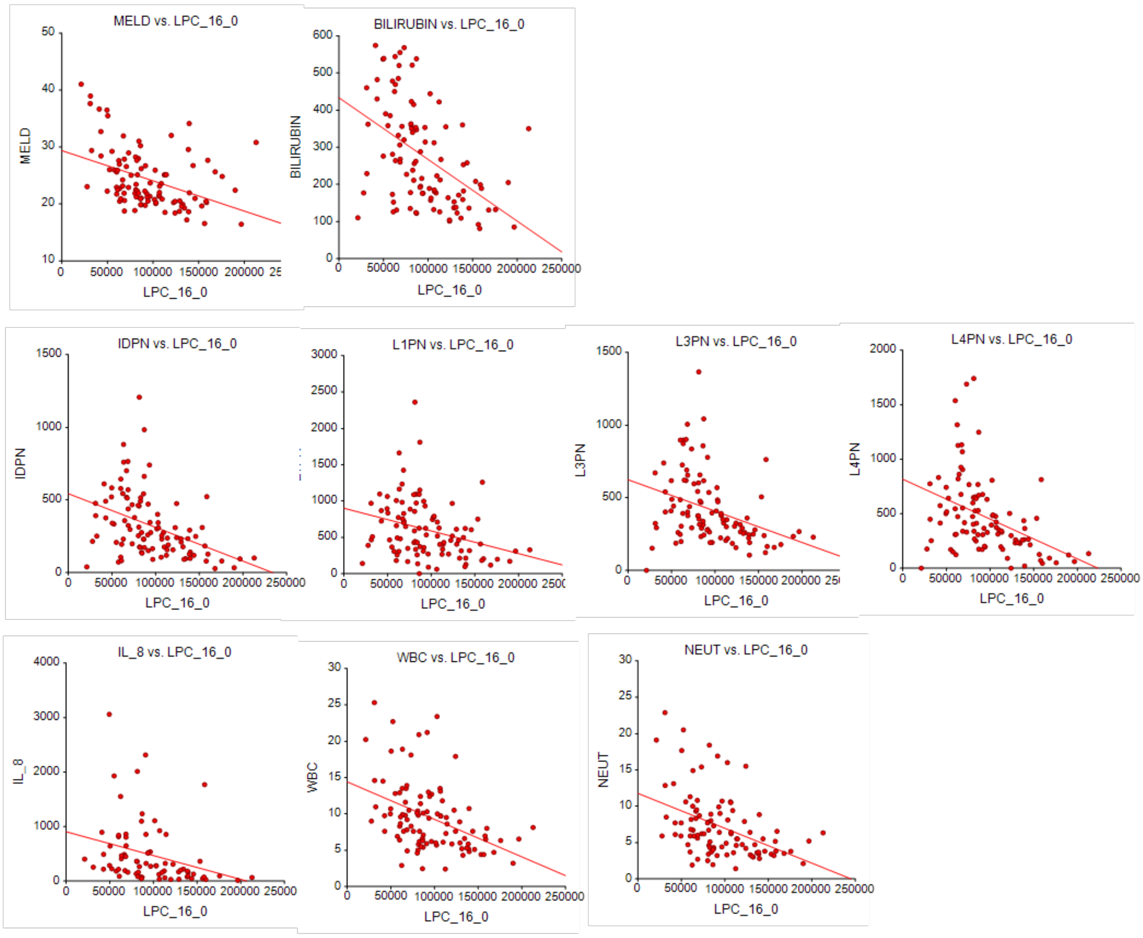

B

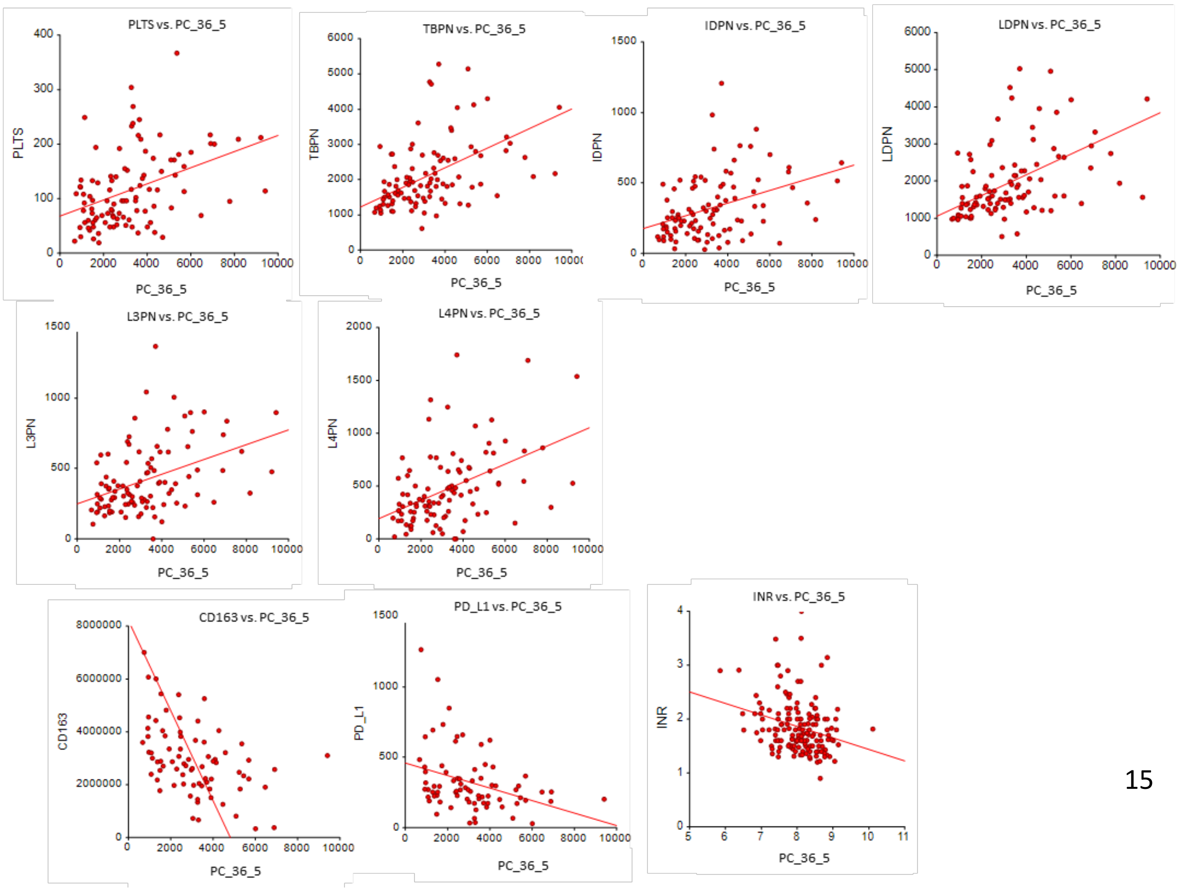

Legend to Fig. S4. A. Correlation plots of LPC(16:0) with MELD, bilirubin level, IDPN, L1PN, L3PN, L4PN, IL-8, WBC count and neutrophils counts (Spearman correlation coefficient and p value) in patients with sAH (n=78) matched to patients with cirrhosis (n=23) on bilirubin level. B. Correlation plots of PC(36:5) with platelet counts, TBPN, IDPN, LDPN, L3PN, L4PN, CD-163, PD-L1 levels and INR (Spearman correlation coefficient and p value) in patients with sAH (n=78) matched to patients with cirrhosis (n=23) on bilirubin level.

Supplementary figure 5

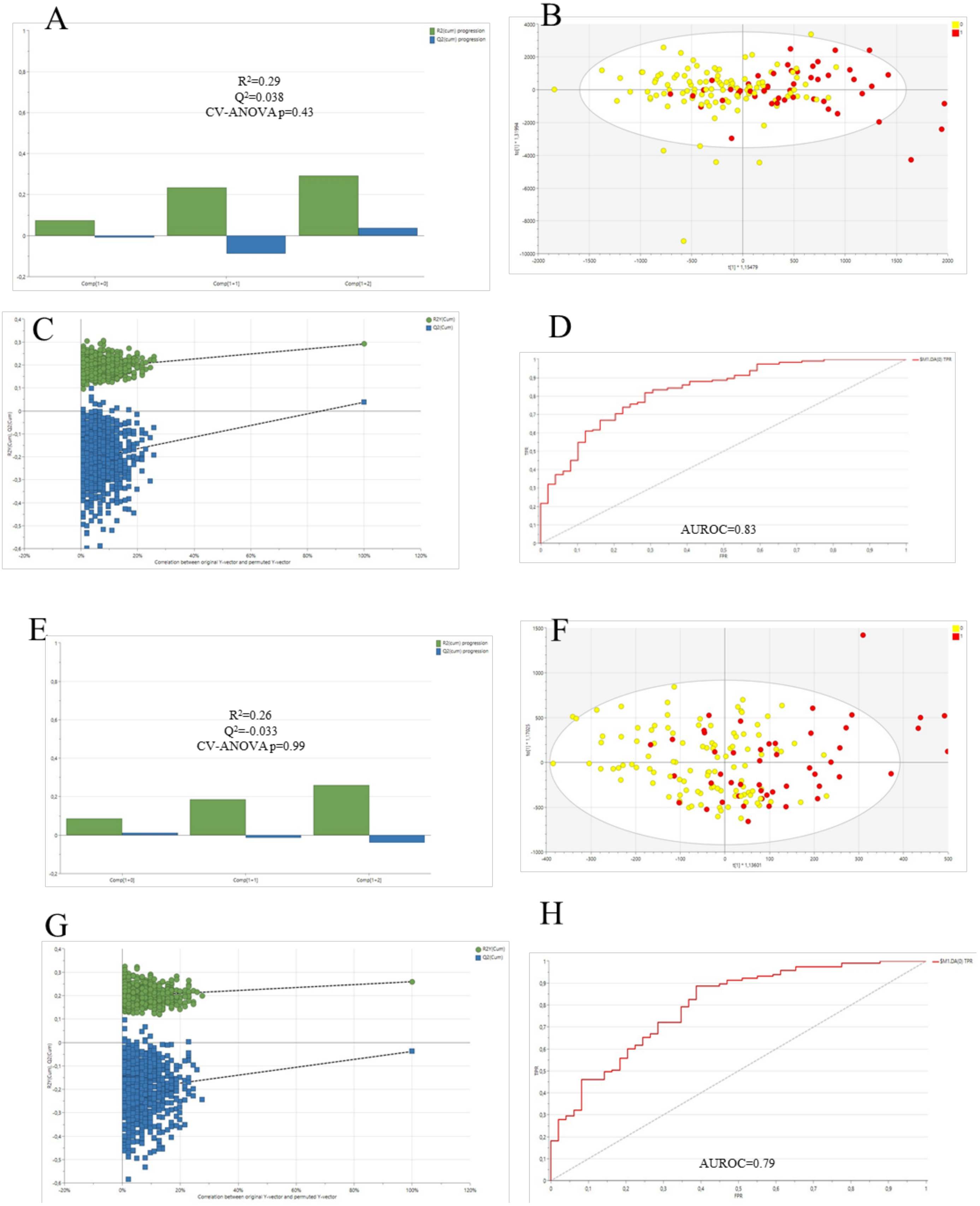

Legend to Fig. S5. A. summary of fit of the three-component model in OPLS-DA to differentiate between D90 survivors and non survivors in positive ionisation mode. B. Scores plot of the model in positive ionisation mode: each dot representing the model in one patient; D90-survivors in yellow, D90 non-survivors in red. C. Permutation test of the model in positive ionisation mode D. AUROC using the discriminant variables of the OPLS-DA model in positive ionisation mode. E. summary of fit of the three-component model in OPLS-DA to differentiate between D90 survivors and non survivors in negative ionisation mode. F Scores plot of the model in negative ionisation mode: each dot representing the model in one patient; D90-survivors in yellow, D90 non-survivors in red. G. Permutation test of the model in negative ionisation mode H. AUROC using the discriminant variables of the OPLS-DA model in negative ionisation mode.

Supplementary figure 6

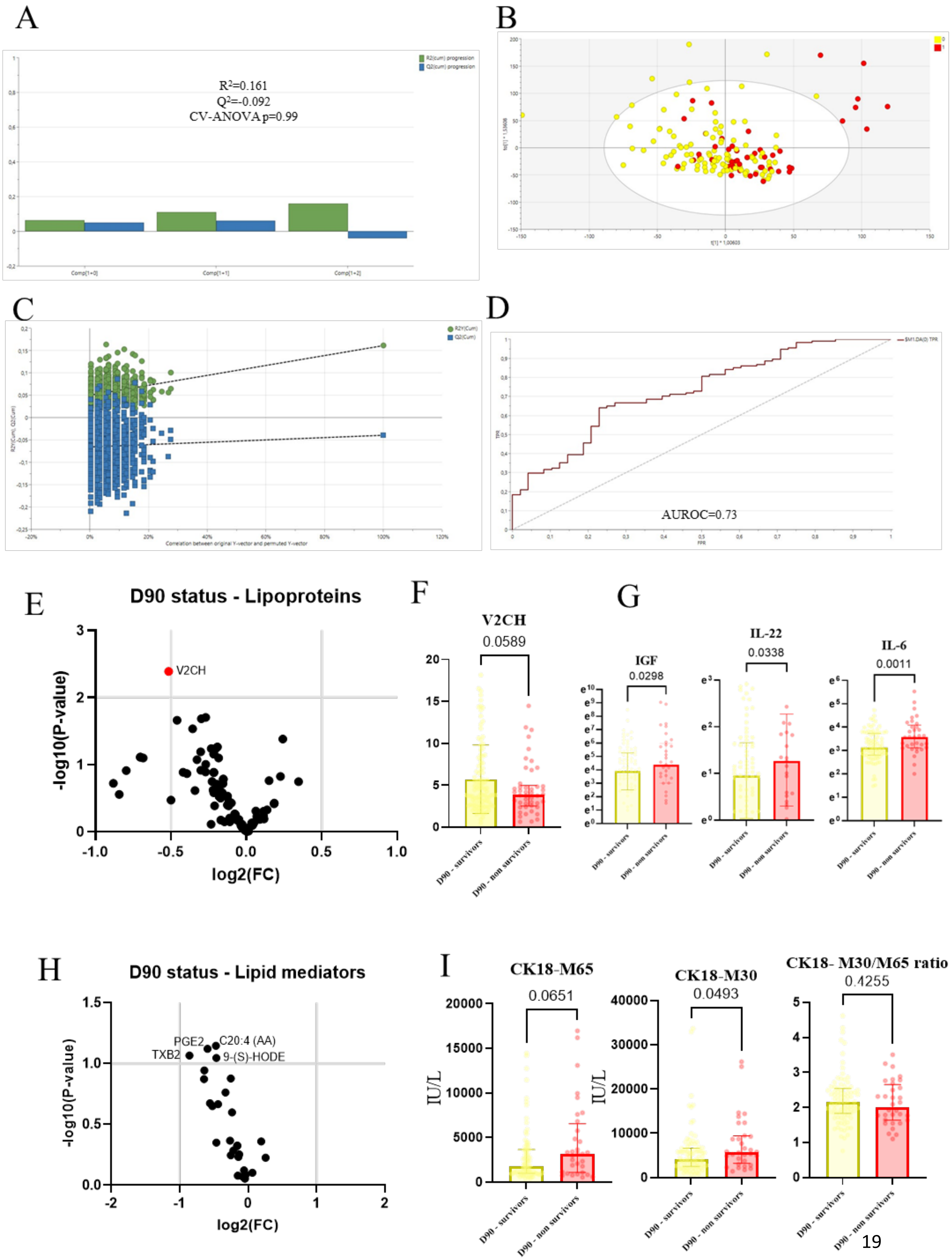

Legend to Fig. S6: Analyses restricted to patients with sAH based on status on day 90 (survivors n=106; non-survivors n=51). A. analyses of targeted lipoproteomics: summary of fit of the OPLS-DA model in positive ionisation mode with 1+2+0 component: R^2=0.161, Q^2=-0.092, CV-ANOVA p=0.99. B. Scores plot: each dot representing one patient; survivors in yellow, non-survivors in red. C. Permutation test demonstrating the non-validity of the model. D. AUROC of the model. E. Volcano plot of each lipoprotein with respect to the status at 90 days (survivors vs. non-survivors). F. Univariable analysis of V2CH levels with respect to the status at 90 days (survivors vs. non-survivors). G. Univariable analyses of cytokines differentiating between survivors vs. non-survivors at 90 days. H. Volcano plot of each lipid mediators with respect to the status at 90 days (survivors vs. non-survivors). I. Univariable analyses of cytokeratin 18 M65 and M30 fragments levels and their ratio with respect to status at 90 days (survivors vs. non-survivors).

Supplementary figure 7

A

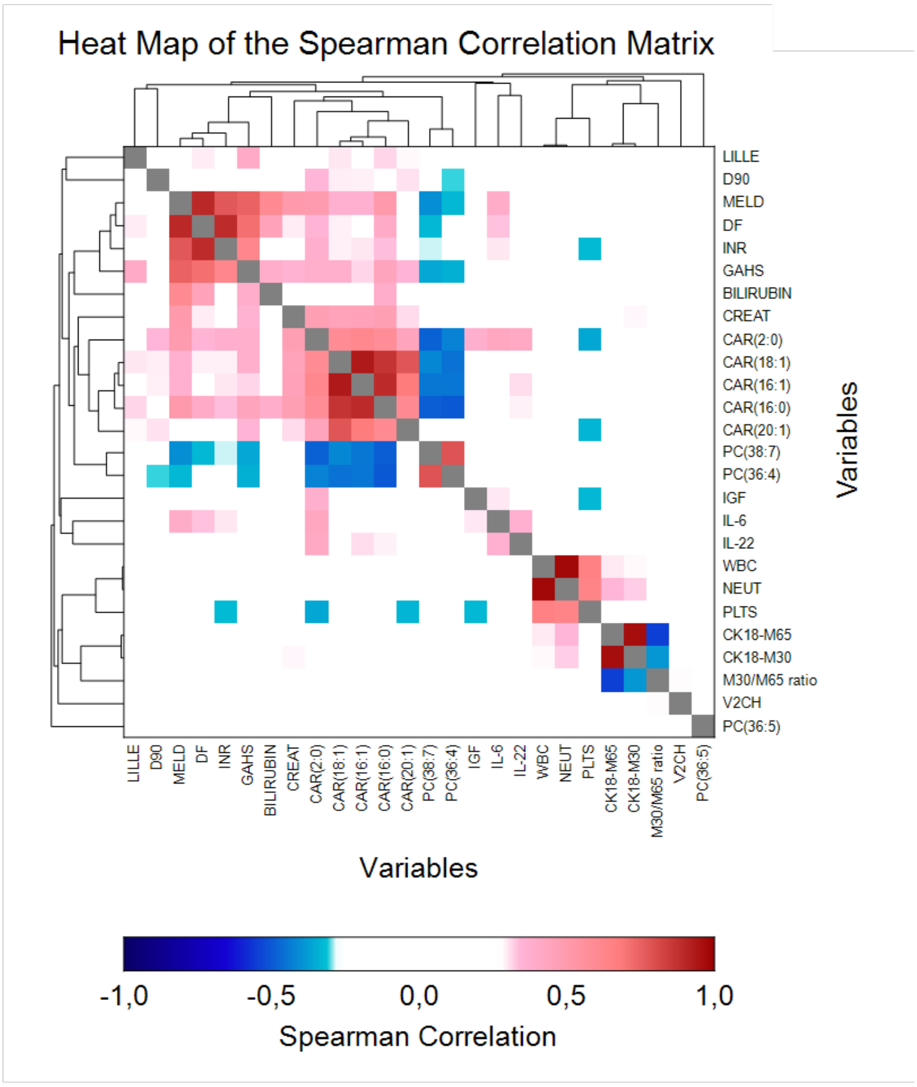

B

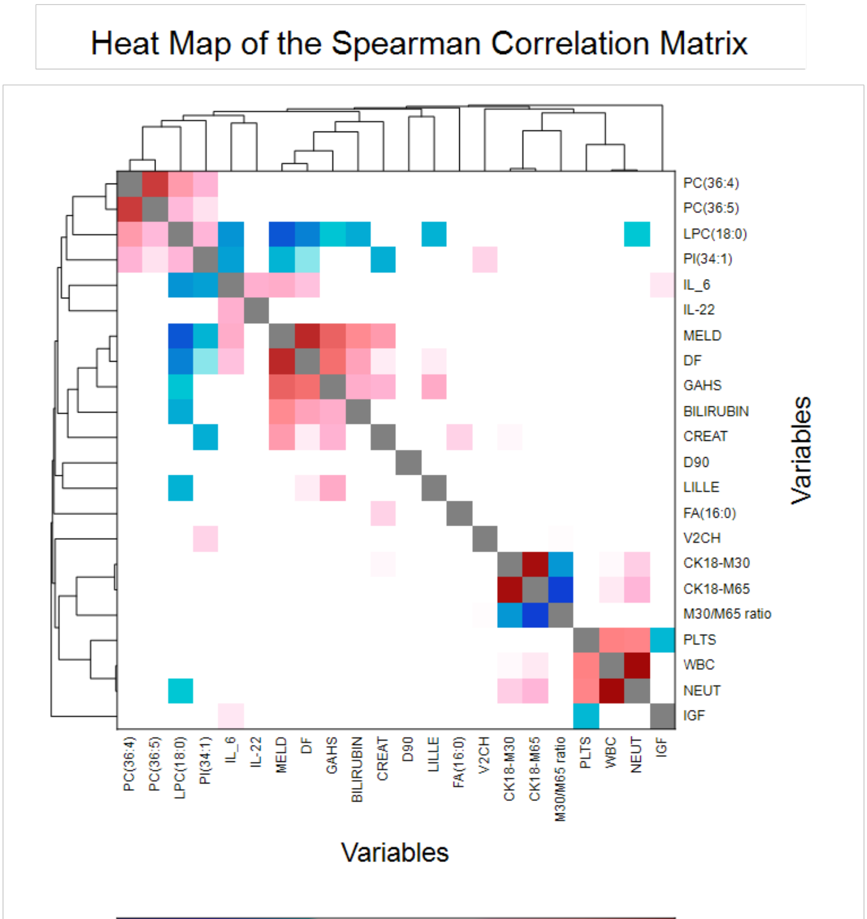

Legend to Fig. S7: Correlation matrix in in patients with sAH included in the final analyses (n=159) based on Spearman's test between A. clinical, laboratory, lipids annotated in positive ionisation mode on volcano plots (Figure 4A), lipoproteins and cytokines identified in Figure 5E-G and CK-18 M65 and M30 fragments and their ratio. B. clinical, laboratory, lipids identified in negative ionisation mode on volcano plots (Figure 4D), lipoproteins and cytokines identified in Figure 5E-G and CK-18 M65 and M30 fragments and their ratio.

Supplementary figure 8

A

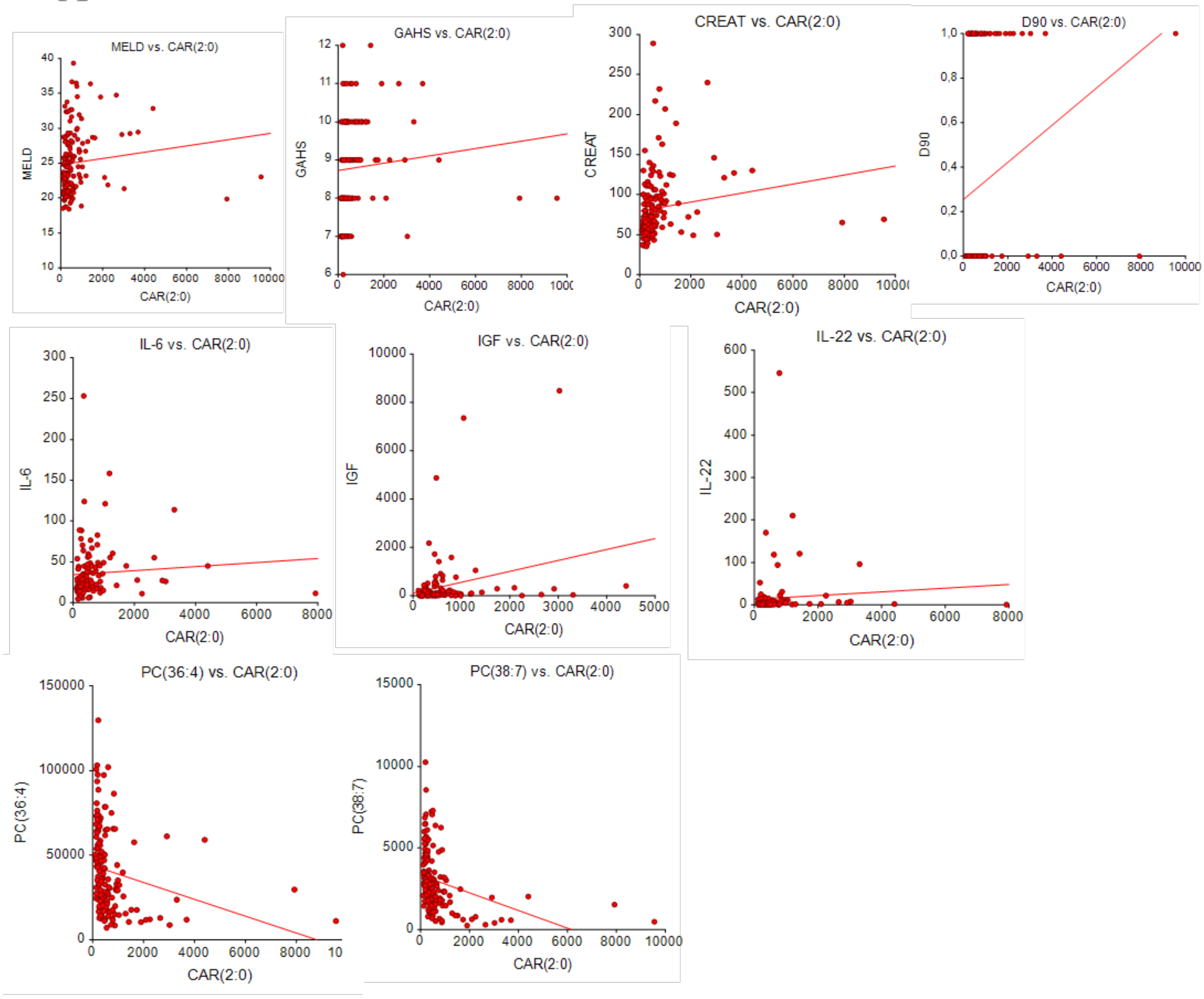

B

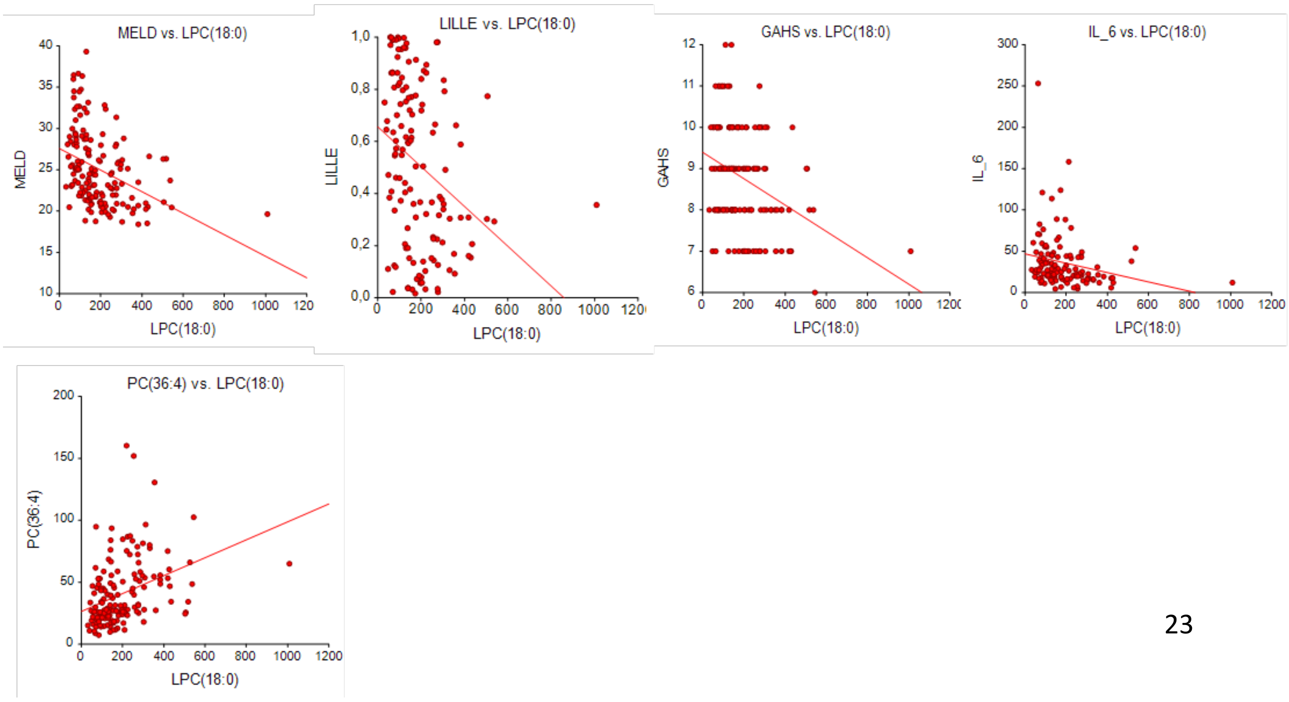

Legend to Fig. S8. Correlation plots of CAR(2:0) with MELD, Glasgow score, creatinine, D90 status, IL-6, IGF, IL-22, PC(36:4), PC(38:7) (Spearman correlation coefficient and p value) in patients with sAH (n=159). B. Correlation plots of LPC(18:0) with MELD, Lille score, Glasgow score, IL-6 and PC(36:4) (Spearman correlation coefficient and p value) in patients with sAH (n=159).

Supplementary Figure 9.

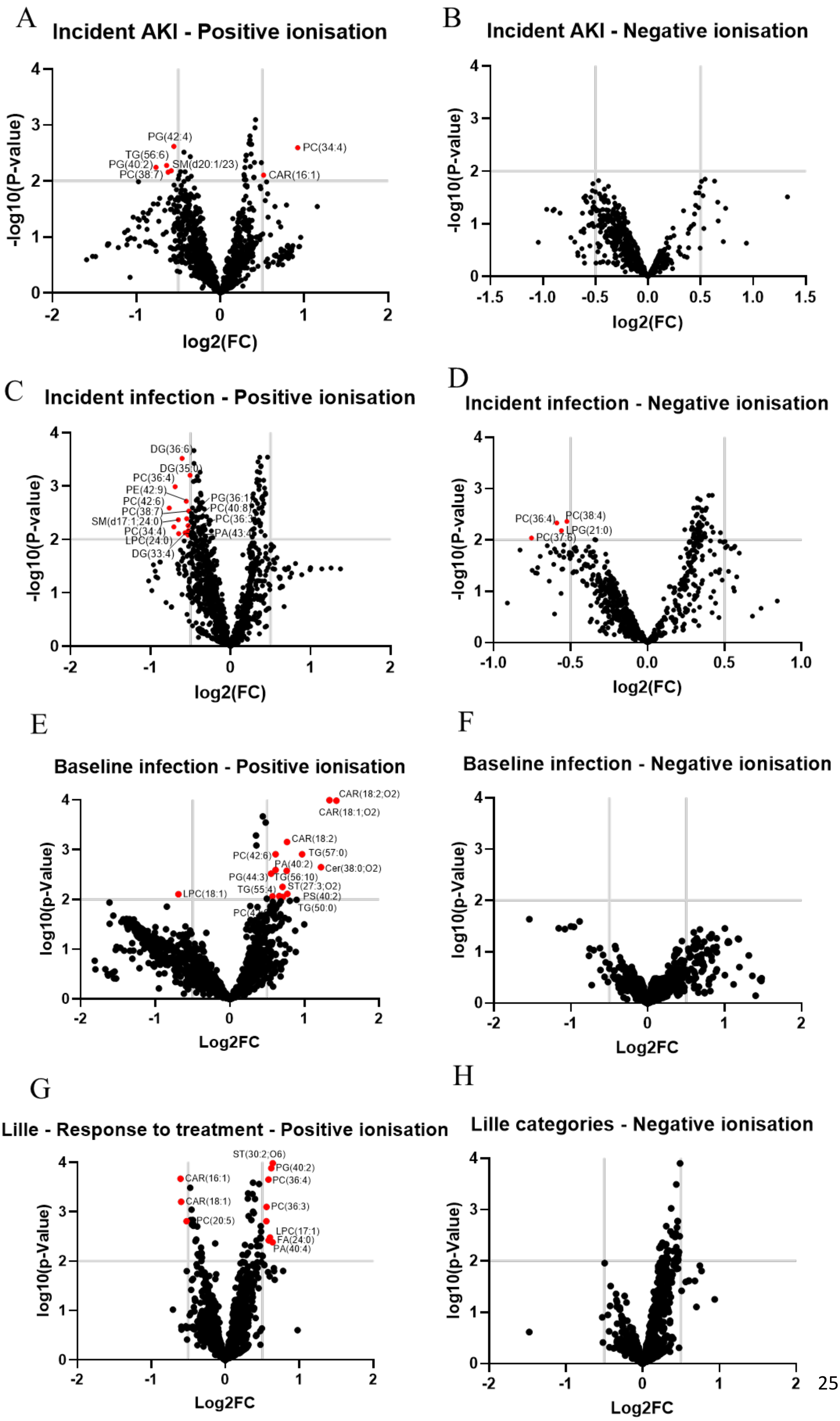

Legend to Fig. S9. A and B. Plots of each peak of positive (A) and negative (B) ionisation modes with respect to incident acute kidney injury (AKI) status: red plot identifying peaks above -0.5;0.5 log2(FC) threshold and 2 -log10(P-value) threshold that were further annotated based on raw chromatograms. C and D. Plots of each peak of positive (A) and negative (B) ionisation modes with respect to incident infection status: red plot identifying peaks above -0.5;0.5 log2(FC) threshold and 2 -log10(P-value) threshold that were further annotated based on raw chromatograms. D and E. Plots of each peak of positive and negative ionisation modes with respect to baseline infection status: red plot identifying peaks above -0.5;0.5 log2(FC) threshold and 2 -log10(P-value) threshold that were further annotated based on raw chromatograms. E and F. Plots of each peak of positive and negative ionisation modes with respect to response to treatment according to Lille model status: red plot identifying peaks above -0.5;0.5 log2(FC) threshold and 2 -log10(P-value) threshold that were further annotated based on raw chromatograms

Supplementary Figure 10.

A

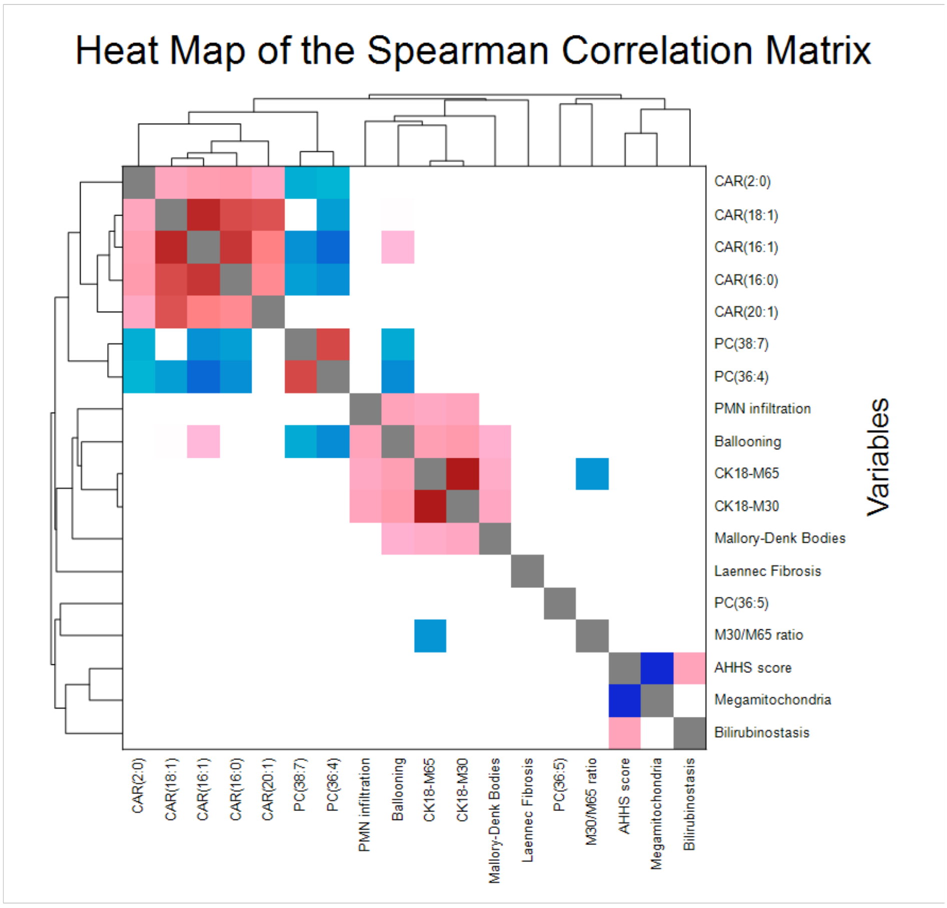

B

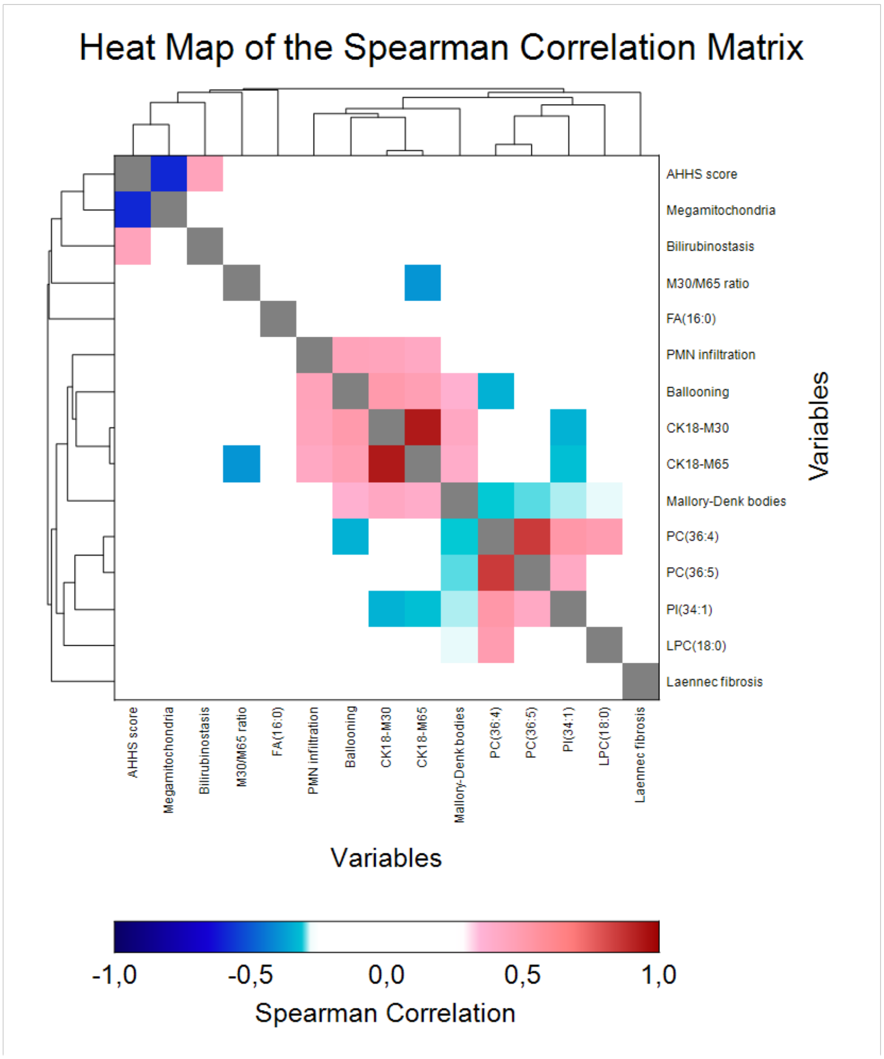

Legend to Fig. S10: Correlation matrix in in patients with sAH with positive biopsy (n=76) based on Spearman's test between A. lipids identified in positive ionisation mode on volcano plots (Figure 4A), CK-18 M65 and M30 fragments and their ratio and AHHSS score and group, fibrosis, neutrophils infiltration, ballooning, Mallory-Denk bodies, bilirubinostasis, and megamitochondria presence. Inflammation and steatosis as defined by AHHSS were not included as constant in the cohort with positive liver biopsy feature. B. clinical, laboratory, lipids identified in negative ionisation on volcano plots (Figure 4C), CK-18 M65 and M30 fragments and their ratio, AHHSS score and group, fibrosis, neutrophils infiltration, ballooning, Mallory-Denk bodies, bilirubinostasis, and megamitochondria presence. Inflammation and steatosis as defined by AHHSS were not included as constant in the cohort with positive liver biopsy feature.

Supplementary tables

Table S1

Characteristics of patients at time of sampling in patients who were biopsied with features of alcohol related hepatitis (n=76) and patients with clinical history of alcohol related hepatitis who did not underwent biopsy (n=83). Continuous and categorical variables expressed respectively in median (interquartile range) and N (percentages). Student's t-test or Mann-Whitney U test for quantitative variables or Chi-Square and Fisher exact tests for categorical variables were used as appropriate.

**Characteristics**			
Age (years)	49.0 (42.8-56.7)	53.0 (43.8-57.8)	0.09
Sex (male)	52 (68.4)	51 (51.5)	0.61
BMI (kg/m^2)	25.4 (22.3-29.8)	26.1 (22.1-31.5)	0.39
Alcohol related liver disease	76 (100)	83(100)	0.99
Biopsy (yes)	76 (100)	-	-
**Laboratory**			
Leukocytes (G/l)	9.2 (6.2-12.3)	10.1 (7.6-13.5)	0.23
Neutrophils (G/l)	6.2 (4.0-9.4)	7.7 (5.5-10.6)	0.22
Hemoglobin (g/l)	108 (99.0-122.0)	111.0 (92.0-121.0)	0.33
Platelets (G/l)	137.0 (86.0-185.0)	113.0 (78.0-189.0)	0.72
INR	1.6 (1.5-2.0)	1.8 (1.6-2.1)	0.11
Bilirubin (mmol/l)	359.0 (260.0-511.0)	325.0 (227.0-433.0)	0.07
AST (IU)	132.0 (96.0-160.0)	136.0 (100.0-179.0)	0.13
ALP (IU)	179.0 (132.0-254.0)	183.0 (134.0-252.0)	0.92
Albumin (g/l)	24.0 (22.0-30.0)	24.0 (21.0-29.0)	0.21
Creatinine (mmol/l)	70.0 (59.0-102.0)	72.0 (52.0-96.0)	0.96
Urea (mmol/l)	3.4 (2.4-5.9)	3.7 (2.3-6.0)	0.46
Sodium (mmol/l)	136.0 (133.0-138.0)	132.0 (127.0-134.0)	<0.0001
**Scores**			
MELD	23.5 (20.9-26.7)	24.6 (21.9-28.1)	0.42
Maddrey DF	54.3 (40.1-74.5)	60.0 (56.8-77.7)	0.28
Lille	0.40 (0.2-0.7)	0.6 (0.3-0.8)	0.06
**Outcome**			
Death at 3 months	13 (17.1)	38 (46.0)	<0.0001

Table S2

Characteristics of patients at time of sampling (overall cohort, N=233; patients with alcohol related hepatitis n=159; patients with cirrhosis n=74). Continuous and categorical variables expressed respectively in median (interquartile range) and N (percentages). Mann-Whitney U test for quantitative variables or Chi-Square and Fisher exact tests for categorical variables were used as appropriate.

**Characteristics**				
Age (years)	51.5 (43.6-58.6)	50.9 (43.1-57.6)	54.0 (45.0-61.8)	0.10
Sex (male)	160 (66.7)	109 (65.7)	51 (68.9)	0.61
BMI (kg/m^2)	25.4 (22.4-30.1)	26.0 (22.4-30.8)	24.0 (22.6-27.7)	0.02
Alcohol related liver disease	224 (93.3)	166 (100)	58 (78.4)	<0.0001
Biopsy (yes)	-	83 (50)	-	-
**Laboratory**				
Leukocytes (G/l)	8.2 (5.8-12.3)	9.5 (6.8-13.0)	6.3 (4.7-8.6)	<0.0001
Neutrophils (G/l)	5.7 (3.6-9.3)	6.6 (4.5-10.1)	3.6 (2.8-5.9)	0.0005
Hemoglobin (g/l)	109.0 (95.8-122.0)	109 (97.0-121.0)	106.0 (90.0-129.0)	0.68
Platelets (G/l)	117.0 (77.0-183.0)	119.0 (79.0-186.0)	109.0 (68.0-176.0)	0.32
INR	1.7 (1.4-2.0)	1.7 (1.5-2.0)	1.4 (1.2-1.9)	0.14
Bilirubin (µmol/l)	268.0 (123.0-422.0)	334.0 (231.0-459.0)	41.0 (21.0-118.0)	<0.0001
AST (IU)	100.0 (54.0-154.0)	134.0 (100.0-170.0)	47.0 (32.0-78.0)	0.0005
ALP (IU)	162.0 (116.0-237.0)	182.0 (133.0-253.0)	125.0 (83.0-166.0)	<0.0001
Albumin (g/l)	27.0 (22.0-33.0)	24.0 (21.0-29.0)	33.0 (25.0-36.0)	<0.0001
Creatinine (µmol/l)	72.0 (58.0-103.0)	71.0 (57.0-98.0)	74.0 (62.0-114.0)	0.05
Urea (mmol/l)	3.7 (2.5-6.6)	3.5 (2.4-5.9)	4.9 (3.0-9.3)	<0.0001
Sodium (mmol/l)	135.0 (131.0-138.0)	134.0 (129.0-137.0)	137.0 (134.0-140.0)	<0.0001
**Scores**				
MELD	22.9 (19.8-27.0)	24.2 (21.5-27.8)	15.0 (10.9-24.6)	<0.0001
Maddrey DF	56.0 (43.4-75.3)	56.0 (43.4-75.3)	-	-
Lille	0.5 (0.2-0.8)	0.5 (0.2-0.8)	-	-
**Outcome**				
Death at 3 months	62 (25.8)	52 (31.3)	10 (13.5)	0.004

Table S3

The 29 lipids putatively identified from the 74 features with a VIP value ≥ 2 in the positive ionisation mode.

PC(34:1)	$[M+H]^+$	2	760.587	7.2	11.8081
PC(34:2)	$[M+H]^+$	2	758.571	5.8	10.6465
SM (d18:1/16:0)	$[M+H]^+$	1	703.577	5.2	6.40203
TG(52:2)	$[M+NH_4]^+$	4	876.805	15.7	6.07929
TG(52:3)	$[M+NH_4]^+$	2	874.79	15.4	5.37885
PC(36:3)	$[M+H]^+$	2	784.588	6.0	5.06594
TG(54:3)	$[M+NH_4]^+$	3	902.821	15.7	4.82478
TG(54:4)	$[M+NH_4]^+$	4	900.806	15.5	4.74365
TG(54:5)	$[M+NH_4]^+$	5	898.79	15.2	4.52367
PC(36:1)	$[M+H]^+$	6	788.62	9.5	4.37981
TG(52:4)	$[M+NH_4]^+$	4	872.774	15.2	4.21215
PC(36:2)	$[M+H]^+$	2	786.602	7.7	3.97121
TG(50:2)	$[M+NH_4]^+$	4	848.774	15.4	3.54993
PC(38:3)	$[M+H]^+$	3	812.62	8.3	3.42408
TG(50:1)	$[M+NH_4]^+$	2	850.79	15.7	3.42084
PC(O-38:5)	$[M+H]^+$	1	794.608	6.7	3.39092
SM (d18:2/24:0)	$[M+H]^+$	4	813.688	11.9	3.39012
TG(54:2)	$[M+NH_4]^+$	3	904.837	16.0	3.369
TG(50:3)	$[M+NH_4]^+$	5	846.758	15.1	3.34927
PC(36:5)	$[M+H]^+$	1	780.557	4.7	3.25165
PC(36:4)	$[M+H]^+$	2	782.572	4.9	2.99232
TG(48:2)	$[M+NH_4]^+$	4	820.743	15.1	2.81332
PC(38:4)	$[M+H]^+$	5	810.604	7.4	2.62413
LPC(16:0)	$[M+H]^+$	1	496.34	1.1	2.61124
PC(38:5-OH)	$[M+H]^+$	3	808.589	6.2	2.52024
TG(50:4)	$[M+NH_4]^+$	4	844.742	14.9	2.29547
TG(56:6)	$[M+NH_4]^+$	6	924.806	15.3	2.2495
TG(56:8)	$[M+NH_4]^+$	4	920.775	14.9	2.1741
PC(36:5)	$[M+H]^+$	1	766.576	6.3	2.0883

Table S4

The 11 lipids putatively identified from the 40 features with a VIP value ≥ 2 in the negative ionisation mode.

PC(34:1)	[2M+FA]$^-$	4	1564.160	7.2	2.01514
PI(38:4)	[M-H]$^-$	8	885.552	5.5	3.37548
PC(38:3)	[M+FA-H]$^-$	11	856.611	8.3	2.91123
PC(38:4)	[M-H]$^-$	4	855.597	7.4	2.65136
PC(36:1)	[M+FA]$^-$	8	832.607	9.6	3.97649
PC(36:2)	[M+FA]$^-$	12	831.598	7.7	3.15473
PC(36:5)	[M+FA]$^-$	7	824.544	4.8	2.26431
PC(34:2)	[M+FA]$^-$	2	802.548	5.9	4.78334
PC(32:1)	[M+FA]$^-$	9	776.544	5.6	3.17116
LPC(18:1)	[M+FA]$^-$	0	566.326	1.2	2.2196
LPC(18:2)	[M+FA]$^-$	8	564.327	1.0	2.16906

Table S5

The eight lipids putatively identified above the log2(FC) and -log10(P-value) thresholds on the volcano plot in positive ionisation mode to differentiate between D90 survivors vs. non survivors.

[redacted]						
CAR(2:0)	$[M+H]^+$	2	204.121	0.5	0.98	2.32
PC(36:5)	$[M+H]^+$	2	780.557	4.1	-0.72	2.41
CAR(18:1)	$[M+H]^+$	2	426.357	1.2	0.57	3.17
PC(38:7)	$[M+H]^+$	2	804.556	4.9	-0.69	4.40
CAR(16:1)	$[M+H]^+$	5	398.325	0.9	0.61	4.67
CAR(16:0)	$[M+H]^+$	5	401.345	1.2	0.52	4.88
CAR(20:1)	$[M+H]^+$	2	454.389	1.6	0.53	4.96
PC(36:4)	$[M+H]^+$	2	782.572	4.9	-0.83	5.24

Table S6

The 13 lipids putatively identified above the log2(FC) and -log10(P-value) thresholds on the volcano plot in negative ionisation mode to differentiate between D90 survivors vs. non survivors.

PC(36:4)	$[M+FA-H]^-$	11	829.569	4.9	-0.62	3.43
FA(16:0)	$[M-H]^-$	11	255.232	2.5	0.57	2.98
LPC(18:0)	$[M+FA-H]^-$	1	508.340	1.6	-0.51	2.41
PI(34:1)	$[M-H]^-$	4	835.534	5.4	-0.50	2.28
PC(36:5)	$[M+FA-H]^-$	3	824.547	4.2	-0.64	2.14

Table S7

Univariable and multivariable logistic regression analyses of lipids species associated with D90 status in negative ionisation mode. Effect size calculated per standard deviation increase.

[redacted]						
PC(36:4)	0.42	0.25-0.69	0.0008	0.38<br>0.48*	0.15-0.89<br>0.27-0.88*	0.03<br>0.02*
FA(16:0)	1.58	1.13-2.21	0.009	1.53<br>1.34	1.08-2.19<br>0.58-3.08*	0.02<br>0.32*
LPC(18:0)	0.70	0.47-1.06	0.09			
PI(34:1)	0.52	0.33-0.84	0.006	0.67	0.41-1.09	0.09
PC(36:5)	0.56	0.36-0.88	0.01	1.09	0.71-1.67	0.69

*after adjustement on Lille score (12.69, 2.93-54.98, p=0.0009)

Supplementary references

[1] Sarafian, M. H.; Gaudin, M.; Lewis, M. R.; Martin, F. P.; Holmes, E.; Nicholson, J. K.; Dumas, M. E. Objective set of criteria for optimization of sample preparation procedures for ultra-high throughput untargeted blood plasma lipid profiling by ultra performance liquid chromatography-mass spectrometry. Anal Chem 2014, 86 (12), 5766-5774. DOI: 10.1021/ac500317c

[2] Isaac, G.; Mc Donald, S.; Astaritra, G. Lipid Separation using UPLC with Charged Surface Hybrid Technology. Waters Application Note 720004107en; 2011

[3] Smith, C. A.; Want, E. J.; O'Maille, G.; Abagyan, R.; Siuzdak, G., XCMS: processing mass spectrometry data for metabolite profiling using nonlinear peak alignment, matching, and identification. Anal Chem 2006, 78 (3), 779-87

[4] Vorkas PA, Shalhoub J, Isaac G, Want EJ, Nicholson JK, Holmes E, Davies AH. Metabolic phenotyping of atherosclerotic plaques reveals latent associations between free cholesterol and ceramide metabolism in atherogenesis. J Proteome Res. 2015 Mar 6;14(3):1389-99. doi: 10.1021/pr5009898. Epub 2015 Feb 23. PMID: 25565173.

[5] Development and Validation of a High-Throughput Ultrahigh-Performance Liquid Chromatography–Mass Spectrometry Approach for Screening of Oxylipins and Their Precursors Arnaud M. Wolfer, Mathieu Gaudin, Simon D. Taylor-Robinson, Elaine Holmes, and Jeremy K. Nicholson Analytical Chemistry 2015 87 (23), 11721-11731 DOI: 10.1021/acs.analchem.5b02794

[6] Dona AC, Jiménez B, Schäfer H, Humpfer E, Spraul M, Lewis MR, Pearce JT, Holmes E, Lindon JC, Nicholson JK. Precision high-throughput proton NMR spectroscopy of human urine, serum, and plasma for large-scale metabolic phenotyping. Anal Chem. 2014 Oct 7;86(19):9887-94. doi: 10.1021/ac5025039. Epub 2014 Sep 16. PMID: 25180432.

[7] Jiménez B, Holmes E, Heude C, Tolson RF, Harvey N, Lodge SL, Chetwynd AJ, Cannet C, Fang F, Pearce JTM, Lewis MR, Viant MR, Lindon JC, Spraul M, Schäfer H, Nicholson JK. Quantitative Lipoprotein Subclass and Low Molecular Weight Metabolite Analysis in Human Serum and Plasma by 1H NMR Spectroscopy in a Multilaboratory Trial. Anal Chem. 2018 Oct 16;90(20):11962-11971. doi: 10.1021/acs.analchem.8b02412. Epub 2018 Sep 27. PMID: 30211542.
